# A Proton-Coupled Electron Transfer Strategy to the Redox-Neutral Photocatalytic CO_2_ Fixation

**DOI:** 10.1021/acs.joc.2c02952

**Published:** 2023-02-10

**Authors:** Pietro Franceschi, Elena Rossin, Giulio Goti, Angelo Scopano, Alberto Vega-Peñaloza, Mirco Natali, Deepak Singh, Andrea Sartorel, Luca Dell’Amico

**Affiliations:** † Department of Chemical Sciences, University of Padova, Via Marzolo 1, 35131 Padova, Italy; ‡ Department of Chemical, Pharmaceutical, and Agricultural Sciences, University of Ferrara, Via L. Borsari 46, 44121 Ferrara, Italy

## Abstract

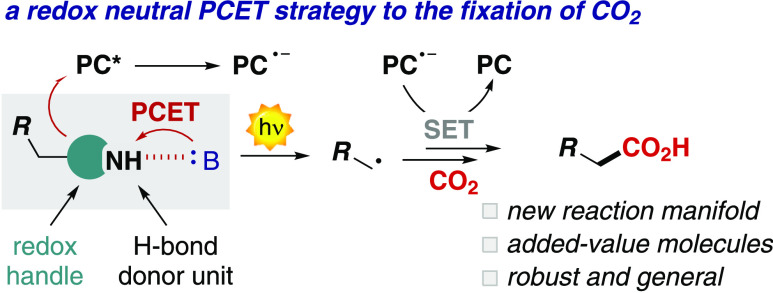

Herein, we report our study on the design and development of a novel photocarboxylation method. We have used an organic photoredox catalyst (PC, 4CzIPN) and differently substituted dihydropyridines (DHPs) in combination with an organic base (1,5,7-triazabicyclodec-5-ene, TBD) to access a proton-coupled electron transfer (PCET) based manifold. In depth mechanistic investigations merging experimental analysis (NMR, IR, cyclic voltammetry) and density-functional theory (DFT) calculations reveal the key activity of a H-bonding complex between the DHP and the base. The thermodynamic and kinetic benefits of the PCET mechanism allowed the implementation of a redox-neutral fixation process leading to synthetically relevant carboxylic acids (18 examples with isolated yields up to 75%) under very mild reaction conditions. Finally, diverse product manipulations were performed to demonstrate the synthetic versatility of the obtained products.

## Introduction

**Scheme 1:**
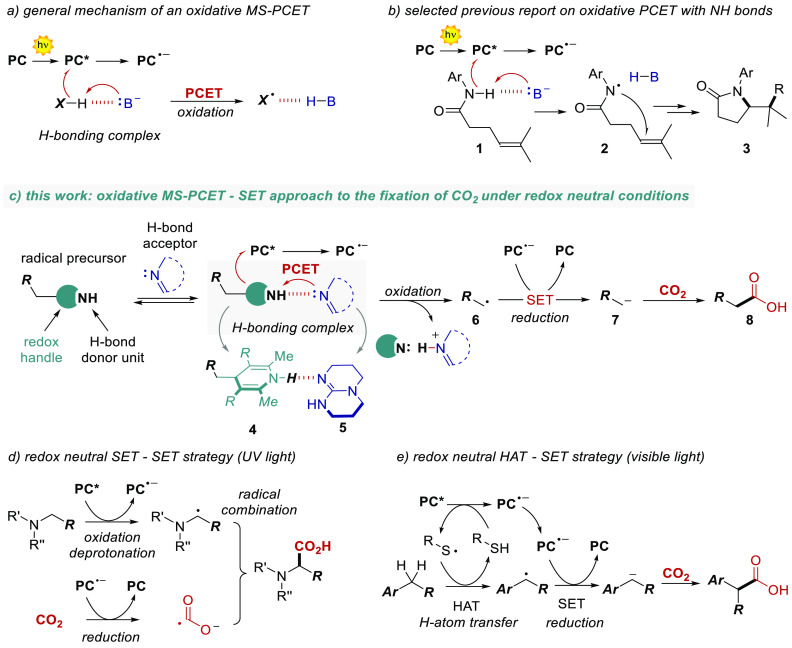
(a) General mechanism for oxidative MS-PCET and (b) selected example of its application to the activation of N–H bonds. (c) Aim of this work: oxidative MS-PCET-SET approach to the fixation of CO_2_ under redox neutral conditions. (d) Previously reported redox neutral SET-SET and (e) HAT-SET strategies for CO_2_ fixation.

In recent years, the tremendous development of synthetic photocatalysis has tackled the deeper comprehension of reaction mechanisms.^1−5^ Recent progresses in this area were guided by the identification of new ways of generating reactive radical species in milder and more controllable conditions. Three main strategies can be distinguished in this context: (i) energy transfer (EnT), (ii) single electron transfer (SET), and (iii) hydrogen atom transfer (HAT). Furthermore, proton-coupled electron transfer (noted as MS-PCET when a proton and an electron move to—or from—different reagents) has been also explored as a convenient approach in the design of new radical-based mechanistic pathways.^6−8^ For example, MS-PCET have been largely investigated in artificial photosynthesis, in particular for the oxygen/hydrogen evolution reactions, and for carbon dioxide reduction.^8−11^ More recently, its importance has been demonstrated also in photosynthetic schemes for the assembly and transformation of organic commodities. In fact, this strategy has been used for the activation of C–H or X–H (X = N, S, O) bonds with the generation of C^•^ or X^•^ radicals.^6,7,12−15^ The oxidative photoinduced MS-PCET (Scheme 1a) is formally a hydrogen equivalent of an hydrogen atom abstraction, while involving the transfer of an electron and a proton to different chemical entities, such as photogenerated oxidant (PC*) and a Brønsted base. A MS-PCET manifold has several benefits: (i) it displays a wider thermodynamic range of action with respect to HAT; (ii) in a MS-PCET, the potential of the oxidant and the strength of the base can be tuned independently, both contributing to an effective bond dissociation free energy (BDFE) of the oxidant/base couple (*vide infra*);^7,8^ (iii) when the transfer of the electron and of the proton from X–H is concerted, the formation of high-energy charged intermediates is avoided, thus lowering the activation barrier of the process.^9^ Given the lower mobility of the proton with respect to the electron, a prerequisite for a concerted process is the preassociation of the X–H group with the base, within a hydrogen bonding network.^7−9^ Examples of photochemical generation of a radical X^•^ upon activation of a X–H bond through an oxidative MS-PCET have been successfully reported for N–H (Scheme 1b),^16^ O–H (alcohols and phenols),^9,17^ and S–H (thiols). Also C–H bonds have been activated for the formation of C^•^ with this strategy, although this approach has never been used for the generation of nucleophilic intermediates.^7,12^ A potential obstacle in promoting oxidative MS-PCET of C–H bonds is their relative reluctance in being preorganized in hydrogen bonding with the base; in previous examples, this condition was achieved by exploiting an intramolecular design properly orienting the C–H and base partners.^12^

We thus sought to use the thermodynamic benefits of a MS-PCET to the generation of charged nucleophilic intermediates such as carbanions, with the final aim of developing a redox-neutral photochemical carboxylation process (Scheme 1c).^18^ Because of its synthetic relevance and the high interest of the whole scientific community toward the fixation of CO_2_, we tested our hypothesis in the reaction between benzylic carbanions and CO_2_.^18−21^ In particular, we envisioned an oxidative PCET with dihydropyridines (DHPs **4**, in Scheme 1c) and an organic base (TBD **5**) as the source of radicals,^22^ followed by a reductive step of the radical **6**, resulting in the generation of the carbanion **7**, able to intercept CO_2_. The oxidative and reductive steps are promoted in the photochemical cycle by the excited state and the reduced form of an organic PC,^23,24^ respectively. The preorganization of the DHP substrate **4** in a hydrogen bonding complex with the base **5** is pivotal to drive the oxidative PCET step.

The previously successful approaches in this area have involved a UV-light mediated SET-SET processes (Scheme 1d),^25^ a HAT-SET approach (Scheme 1e),^26^ or redox-unbalanced SET-SET mechanisms (not shown).^27^ To the best of our knowledge, a redox-neutral and PCET-based strategy has never been reported, despite the high generality that a PCET manifold can offer.

## Results and Discussion

We initiated our study by selecting 4BnDHP **4a** as the redox-active radical source that embodies a NH moiety. We tested by cyclic voltammetry the effect of organic bases on the oxidation of **4a** in dimethylformamide (DMF) as the solvent (Figure S7). Under anodic scan, **4a** (10^–3^ M in DMF) shows an irreversible wave peaking at *E*_pa_ = +0.59 V vs Fc^+^/Fc (*E*_pa_ = anodic peak potential, Fc = ferrocene), and ascribable to one electron oxidation of **4a** and subsequent C–C homolytic cleavage.^28,29^ In the presence of a base (1.5 equiv), the anodic process shifts to lower potentials, with the higher shift observed for TBD and 1,1,3,3-tetramethylguanidine (TMG) bases (*E*_pa_ = +0.37 and +0.35 V vs Fc^+^/Fc for TBD and TMG, respectively), suggesting a more favorable oxidation of **4a** in the presence of a base.

**Figure 1:**
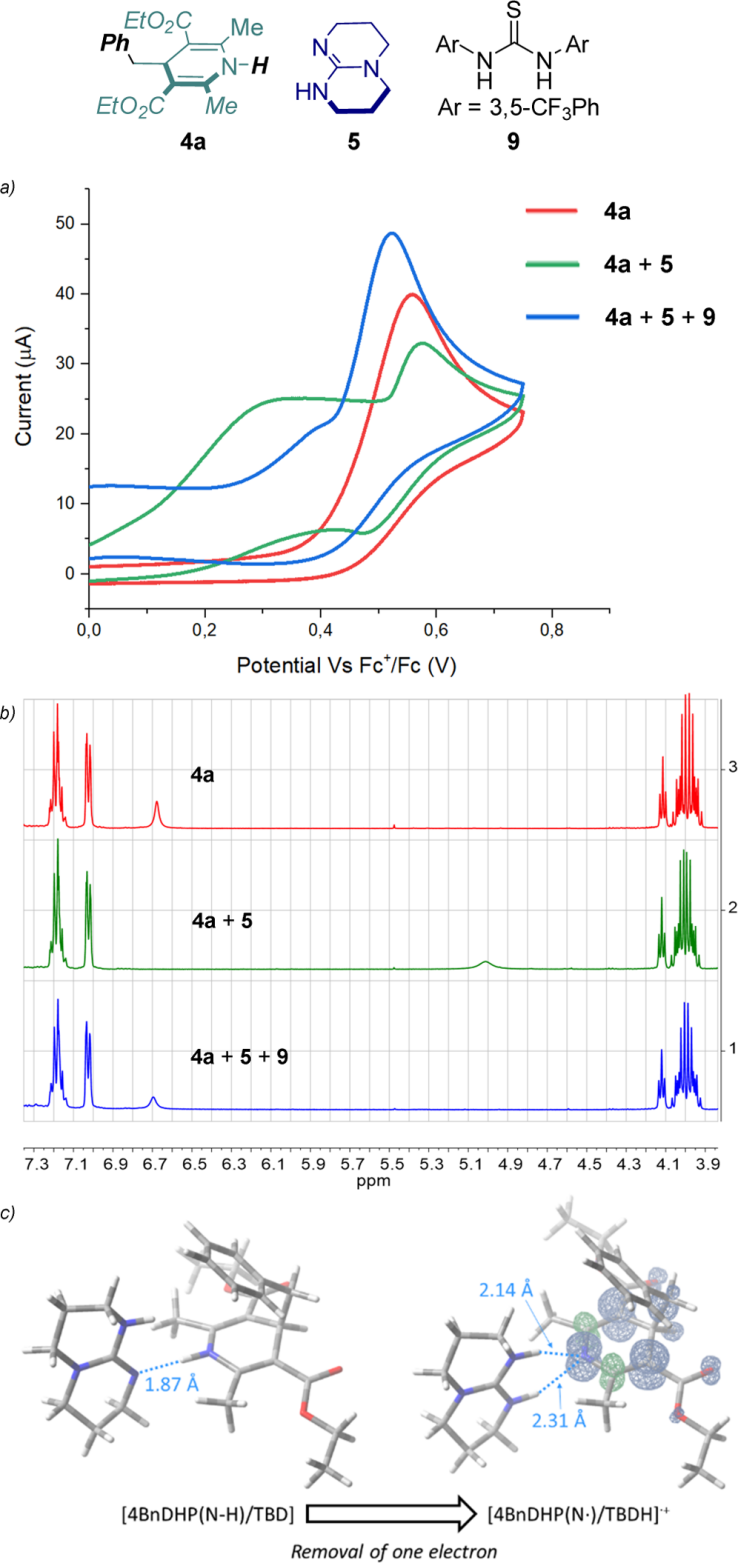
(a) Cyclic voltammograms and (b) ^1^H NMR spectra of **4a** (red), **4a** + TBD **5** (green), **4a** + TBD **5** + Schreiner’s thiourea **9** (blue). (c) Optimized geometries of 4BnDHP/TBD at the ground (left) and [4BnDHP/TBD]^•+^ oxidized (right) states, at the B3LYP/6-311+g(d,p) level of theory, including a polarizable continuum model of acetonitrile solvent. The spin density in [4BnDHP/TBD]^•+^ is also represented and is entirely localized in the DHP moiety (right).

The effect is observed also in acetonitrile (MeCN) as the solvent, with a representative case with TBD reported in Figure 1a. In MeCN, **4a** shows an *E*_pa_ = +0.51 V vs Fc^+^/Fc, while in the presence of TBD the wave is decreased in intensity and a new process appears at *E*_pa_ = +0.31 V vs Fc^+^/Fc (in the same potential range, the TBD alone gives two anodic processes peaking at *E*_pa_ = +0.49 and +0.92 V vs Fc^+^/Fc). Interestingly, the addition of the Schreiner’s thiourea (a well-established H-bond donor) restores the initial wave typical of **4a**.

These results indicate that the role of TBD is to induce a MS-PCET within a 4BnDHP/TBD hydrogen-bonded adduct. With the aim of further confirming these findings, we conducted additional analysis by using ^1^H NMR, FT-IR, UV–vis, and DFT calculations.

By ^1^H NMR analysis, the N–H signal of **4a** shows a shift from 6.7 to 5.0 ppm after the addition of TBD, accompanied by a broadening of the peak (from 8 to 32 Hz; blue and green traces in Figure 1b). Conversely, the other signals of **4a** undergo negligible changes, thus ruling out a deprotonation of **4a** by TBD.^30^ As expected, the addition of a competitive H-bond donor such as Schreiner’s thiourea restores the original signal at 6.5 ppm of the N–H (red trace in the NMR in Figure 1b).

The absence of major deprotonation of 4BnDHP by TBD was indeed supported by FT-IR in MeCN solution, where the stretching of the N–H bond of **4a** at 3360 cm^–1^ persists also in the presence of 1.5 equiv of TBD (Figure S8).

Consistently, the UV–vis analysis of **4a** (10^–5^ M in MeCN) in the presence of a large excess of TBD (up to 5 × 10^–2^ M) allowed us to estimate a p*K*_a_ value of 30.5 ± 0.3 for the N–H group in **4a** (Figure S9 and see details in Supporting Information), 4.5 times higher than the one associated with the TBDH^+^/TBD couple, p*K*_a_ = 26.^31^

To get insights into the impact of the H-bonding network in the one electron oxidation of the 4BnDHP/TBD adduct, we performed DFT calculations. The one-electron oxidation of the **4a** within the 4BnDHP/TBD was modeled at the B3LYP/6-311+g(d,p) level of theory, with a polarizable continuum model of MeCN solvent.^32,33^Figure 1c reports the optimized geometry of the 4BnDHP/TBD adduct (optimized as a neutral, singlet species), revealing the N–H···N hydrogen bond with N–H and H···N distances of 1.04 and 1.87 Å, respectively, and a NĤN angle of 178.8°.^34,35^ A second geometry with different relative orientations of **4a** and TBD was considered, showing a similar energy and similar distances and angles within the H-bonding network, see Figure S14 in the Supporting Information. In agreement with MS-PCET manifold, when optimizing the 4BnDHP/TBD adduct upon one electron oxidation (positively charged species with doublet multiplicity), the proton from the N–H group in **4a** migrates to the nitrogen of TBD. In the optimized geometry of the resulting state [4BnDHP(N^•^)/TBDH]^•+^, an H bonding is still present and involves the N–H moieties of TBDH^+^ and the N atom of the dihydropyridine, with distances of 2.14 and 2.31 Å. The spin density is entirely localized on the DHP scaffold (Figure 1c). The DFT analysis thus confirms a MS-PCET process in the oxidation of the 4BnDHP/TBD adduct to [4BnDHP(N^•^)/TBDH]^•+^, where the electron is removed from the **4a** with the concomitant proton transfer to the TBD.

With this information in hand, we next evaluated the feasibility of a PCET-based photocarboxylation process. Based on previous reports on light-driven carboxylation methods, we initially evaluated the reaction with the 4CzIPN **11** in DMF under a CO_2_ atmosphere (1 atm) and 435 nm irradiation (Table 1, entry 1). In the absence of a base, no carboxylation product was detected.

**Table 1:**
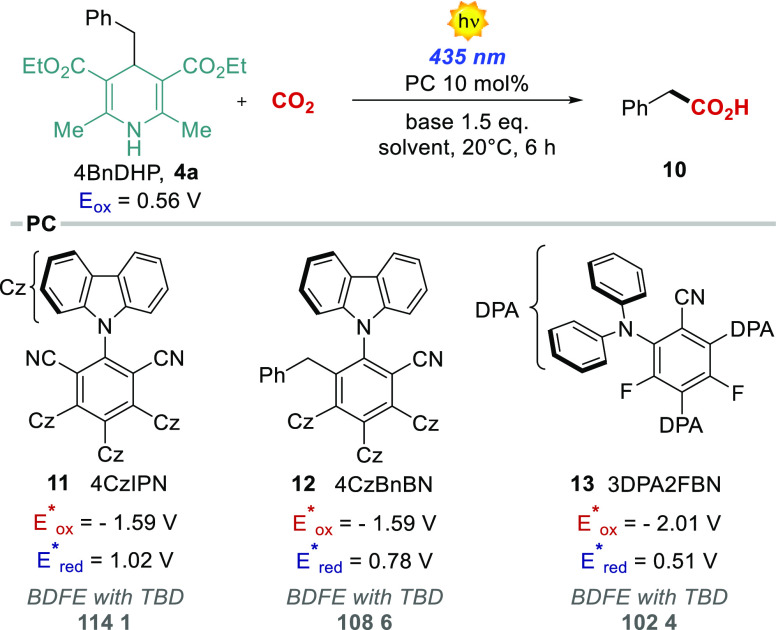
Reaction Scheme and Photocatalysts Employed^a^

entry	base	solvent	concentration (M)	PC	yield^b^ (%)
**1**	–	DMF	0.1	**11**	trace
**2**	NEt_3_	DMF	0.1	**11**	49
**3**	TMG	DMF	0.1	**11**	51
**4**	Me-TBD	DMF	0.1	**11**	58
**5**	TBD	DMF	0.1	**11**	71
**6**	TBD	MeCN	0.1	**11**	68
**7**	TBD	MeCN	0.05	**11**	73
**8**^c^	***TBD***	***MeCN***	***0.05***	**11**	***76***
**9**	TBD	MeCN	0.05	**–**	trace
**10**^c^^,^^d^	TBD	MeCN	0.05	**11**	–
**11**^c^	TBD	MeCN	0.05	**12**	73
**12**^c^	TBD	MeCN	0.05	**13**	71

a E*_ox_ and E*_red_ refer to the potentials of the PC^•+^/PC* and PC*/PC^•–^ couples, respectively;^24^ potentials are reported vs Fc^+^/Fc (for comparison with literature data: *E* vs Fc^+^/Fc = *E* vs SCE – 0.37 V). The BDFE values are calculated according to eq 1 (*vide infra*). Optimization of reaction parameters: General conditions are 0.1 mmol of **4a**, 1 atm. of CO_2_, 1 mL of solvent.

b Yields are given by ^1^H NMR analysis with dibromomethane as internal standard.

c 2 mol % of **11** was used.

d No light irradiation.

As for the CV experiments, we performed a screening of bases (Table 1, entries 2–5). The best performance was obtained with TBD, with 71% yield (Table 1, entry 5). Solvent screening, concentration, and the catalyst loading were later evaluated (Table 1, entries 6–8) to identify the best reaction conditions (Table 1, entry 8, 76% yield). As expected the reactivity was completely suppressed in the absence of the PC or in the dark (Table 1, entries 9–10).

Recently, König et al. reported the evolution of **11** in the presence of benzylic radical precursors into a benzylated derivative 4CzBnBN (**12**), which was shown to be the real active photocatalyst.^36^ Indeed, **12** was verified to form also in our conditions, and consistently the reaction starting from the isolated **12** provides a similar 73% carboxylation yield (Table 1, entry 11). A good yield was obtained also with a less oxidizing photocatalyst 3DPA2FBN **13** (Table 1, entry 12, 71%). Indeed, the excited state of the photocatalyst drives the oxidative MS-PCET within the 4BnDHP/TBD adduct. The energetics of the oxidative MS-PCET process can be discussed on the basis of the effective BDFE formalism (*vide supra*),^7,8^ by combining the potential of the oxidant (in this case the excited photocatalyst) and the p*K*_a_ of the acid/base couple (in this case the TBDH^+^/TBD, p*K*_a_ = 26 in acetonitrile):
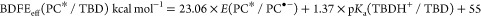
1In particular, the BDFE_eff_ is 114.1, 108.6, and 102.4 kcal mol^–1^ for photocatalysts **11**, **12**, and **13**, respectively, thus being suitable to promote formal hydrogen abstraction from the **4a** substrate (BDFE of the N–H bond ca. 90 kcal mol^–1^).^37^ As a selected case, the reactivity of 12* toward the 4BnDHP/TBD adduct was confirmed through emission experiments. Under deaerated or CO_2_ saturated acetonitrile, the 12* is characterized by a lifetime of 29 ns and of 4.8 μs for the singlet and triplet excited states, respectively (Figure S11; under similar conditions the parent 11* shows a lifetime of 20 ns for the singlet and of 5.1 μs for the triplet,^24,38,39^Figure S12). Upon addition of 4BnDHP/TBD, a progressive decay of both the singlet and the triplet lifetimes is observed, indicative of a dynamic quenching; a Stern–Volmer plot of the triplet lifetime versus the concentration of 4BnDHP/TBD provides a second order rate constant for the quenching of 1.5 × 10^9^ M^–1^ s^–1^, approaching the diffusion limit (Figure S13).

A further investigation on the nature and on the time evolution of the formed species by transient absorption spectroscopy was hampered by the available instrumental setup, which allows for laser excitation at 355 nm, where the substrate **4a** gives competitive absorption.

**Scheme 2:**
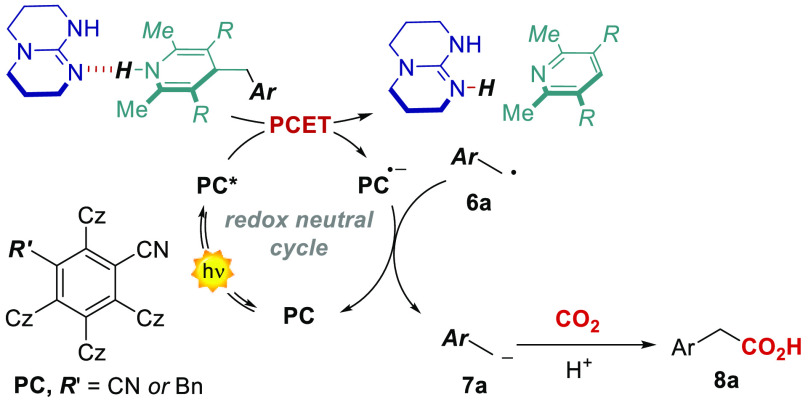
Proposed Reaction Mechanism for the Redox Neutral Photocarboxylation Method Herein Developed

Upon oxidative MS-PCET and fragmentation, the pathway most likely involves a subsequent reduction step of the benzyl radical **6a** by the reduced photocatalyst **12**^•–^ (*E* = −2.17 V vs Fc^+^/Fc for the **12**/**12**^•–^ couple) resulting in the generation of a benzylic carbanion **7a** (estimated *E* ca. −2.4 V vs Fc^+^/Fc for the **6a**/**7a** couple),^40^ through a radical-polar crossover manifold. This mechanism has been recently established as a general and reliable strategy for the generation of reactive nucleophilic intermediates.^26,41^ In this case the presence of an aryl group is key to the stabilization of the carbanion. The benzyl carbanion **7a**(33) is finally capable of reacting with CO_2_ (Scheme 2).^42^

**Figure 2:**
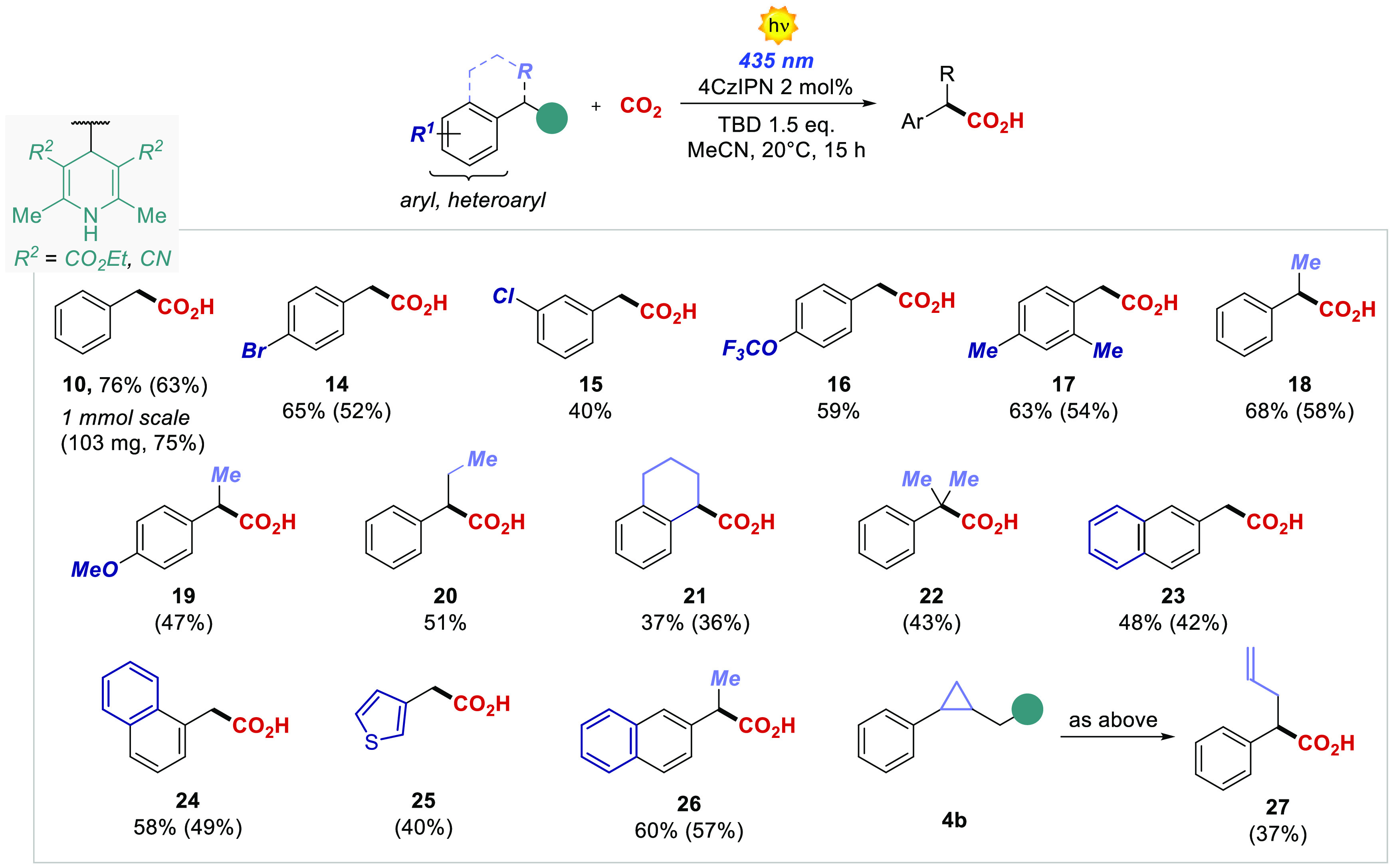
Scope of photochemical carboxylation with DHPs. Yields are given by ^1^H NMR analysis with dibromomethane as internal standard. Isolated yields in parentheses.

After having investigated the mechanism of the reaction, we explored the generality of the carboxylation process by testing different dihydropyridines (Figure 2). Primary aryl radicals were generated efficiently both from electron-rich and electron-deficient substrates, resulting in the corresponding carboxylated products **10**–**17** with isolated yields spanning from 52% to 63%. Remarkably, when scaling up the model reaction to a 1 mmol scale, we obtained the desired phenylacetic acid **10** with an increased 75% isolated yield, 103 mg. Secondary and tertiary precursors were also competent substrates furnishing the carboxylated products **18**–**22** from 36% to 58% yield. We also investigated heteroaromatic DHPs **23**–**26**, obtaining good isolated yields up to 57%.

In order to confirm the radical nature of process, a new cyclopropyl DHP **4b** was synthesized and subjected to the optimized reaction conditions. The carboxylation in this case took place at the more stabilized benzylic position, resulting in the synthesis of a valuable 2-allyl benzoic acid **27**. It is worth mentioning that all the products were isolated without the need of column chromatography.

**Scheme 3:**
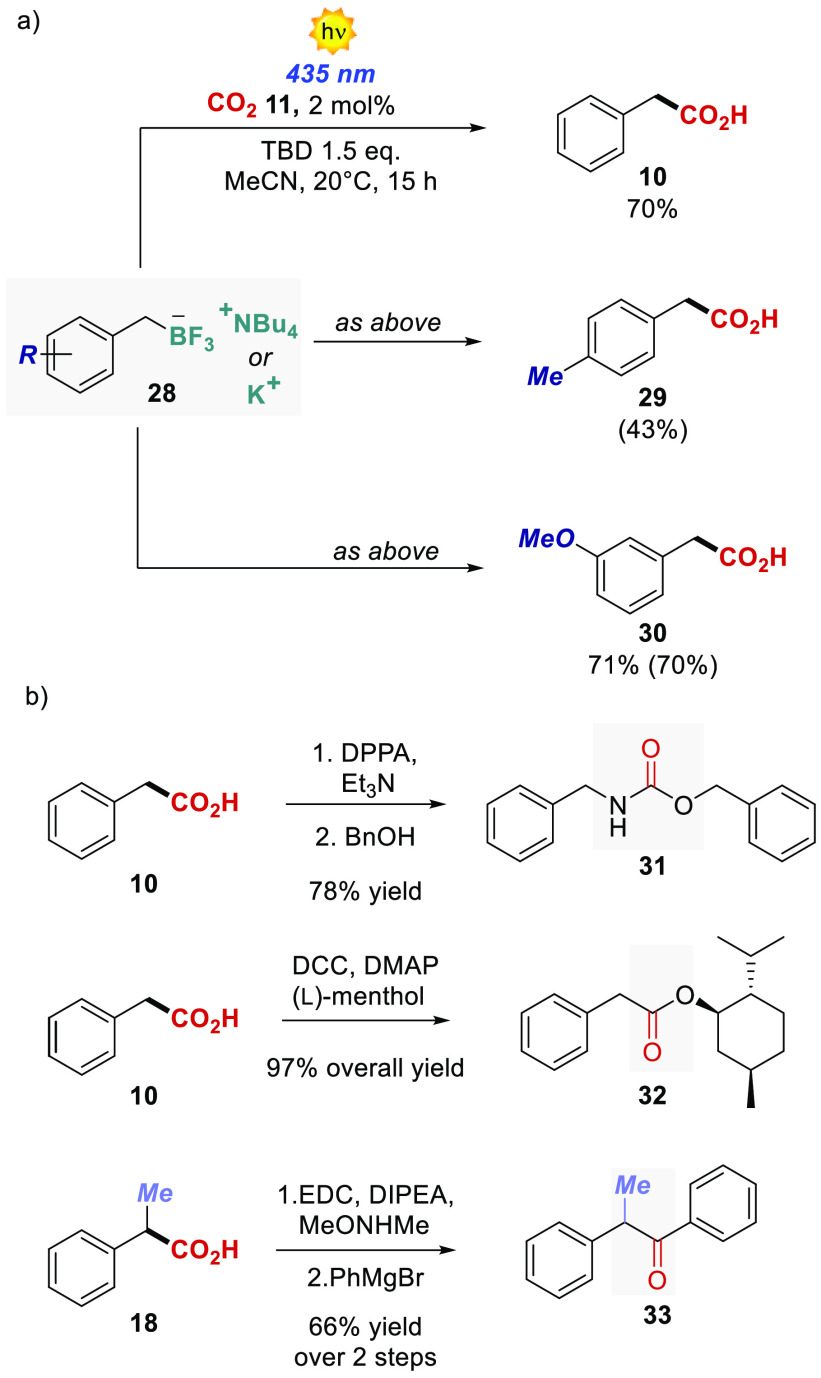
(a) Reaction performed with BF_3_K and BF_3_NBu_4_ salts. (b) Products manipulations. DPPA: diphenylphosphoryl azide; DCC: *N*,*N*′-dicyclohexylcarbodiimide; DMAP: 4-(dimethylamino)pyridine; EDC: *N*-(3-dimethylaminopropyl)-*N*′-ethylcarbodiimide hydrochloride; DIPEA: *N*,*N*-diisopropylethylamine.

Other radical precursors were also investigated (Scheme 3a). Different benzylic BF_3_K or BF_3_NBu_4_ salts afforded the respective products up to 70% isolated yield. In this case, the only operative mechanism is the classical SET, and in agreement with this the less oxidizing PC **13** was not able to promote the carboxylation process (see SI), further confirming our mechanistic findings.

We next demonstrated the synthetic usefulness of the obtained carboxylated products (Scheme 3b). As shown in Scheme 3, **10** was transformed trough a Curtius rearrangement procedure into the carbamate derivative **31** in 78% overall yield. Using l-menthol, the ester **32** was obtained in 97% yield starting from **10**. Finally, product **18** was converted into the corresponding Weinreb amide, and subsequently treated with phenylmagnesium bromide to yield the aryl ketone **33** in 66% yield over two steps.

## Conclusions

In conclusion, a novel photocarboxylation method using CO_2_ was developed. The process works through an innovative redox-neutral PCET-SET manifold that was investigated by experimental analysis (NMR, CV, IR and UV–vis) and supported by DFT calculations. The developed method allows access to a series of diverse benzylic radical intermediates, which were readily converted into the corresponding carbanions capable of engaging in the nucleophilic addition to CO_2_ (isolated yields up to 75%). Finally, we have demonstrated the versatility of the carboxylated products with a series of simple transformations.

## Experimental Section

### General Information

Chromatographic purification of products was accomplished using flash chromatography on silica gel (SiO_2_, 0.04–0.063 mm) purchased from Machery–Nagel, with the indicated solvent system according to the standard techniques. Thin-layer chromatography (TLC) analysis was performed on precoated Merck TLC plates (silica gel 60 GF254, 0.25 mm). Visualization of the developed chromatography was performed by checking UV absorbance (254 and 365 nm) as well as with phosphomolybdic acid and potassium permanganate solutions. Organic solutions were concentrated under reduced pressure on a Büchi rotary evaporator. NMR spectra were recorded on a Bruker Avance 300 spectrometer equipped with a BBO-z grad probehead, a Bruker 400 AVANCE III HD equipped with a BBI-z grad probehead, and a Bruker AVANCE Neo 600 equipped with a TCI Prodigy cryoprobe. The chemical shifts (δ) for ^1^H and ^13^C are given in ppm relative to residual signals of the solvents (CHCl_3_ @ 7.26 ppm ^1^H NMR, 77.2 ppm ^13^C NMR; acetone @ 2.05 ppm ^1^H NMR, 29.84 ppm ^13^C NMR). Coupling constants are given in Hz. The following abbreviations are used to indicate the multiplicity: s, singlet; d, doublet; t, triplet; q, quartet; m, multiplet; qd, quartet of doublets; brs, broad singlet; brd, broad doublet; brt, broad triplet. NMR yields were calculated by using dibromomethane (4.95 ppm, s, 2H) as internal standard. High-resolution mass spectra (HRMS) were obtained using Waters GCT gas chromatograph coupled with a time-of-flight mass spectrometer (GC/MS-TOF) with electron ionization (EI). Steady-state absorption spectroscopy studies have been performed at room temperature on a Varian Cary 50 UV–vis double beam spectrophotometer; 10 mm path length Hellma Analytics 100 QS quartz cuvettes have been used. Nanosecond transient absorption measurements were performed with an Applied Photophysics laser flash photolysis apparatus, using a frequency-doubled (532 nm, 330 mJ) or tripled (355 nm, 160 mJ) Surelite Continuum II Nd/YAG laser (half-width 6–8 ns) as excitation source. Transient detection was obtained using a photomultiplier-oscilloscope combination (Hamamatsu R928, LeCroy 9360). IR measurements were carried out at room temperature on a JASCO FT/IR-4100 spectrophotometer; 1 mm path length Hellma Analytics 100 QX quartz cuvettes have been used. The electrochemical characterizations were carried out at room temperature, on a BASi EC Epsilon potentiostat-galvanostat. A typical three-electrode cell was employed, which was composed of glassy carbon (GC) working electrode (3 mm diameter), a platinum wire as counter electrode, and a silver/silver chloride electrode (Ag/AgCl (NaCl 3 M)) as reference electrode. The reference electrode is a silver wire that is coated with a thin layer of silver chloride; the electrode body contains sodium chloride (NaCl 3 M). The GC electrode was polished before any measurement with diamond paste and ultrasonically rinsed with deionized water for 15 min.

### General Procedures A and B for the Synthesis of 4BnDHP

**A**: 4-Substituted-2,6-dimethyl-1,4-dihydropyridine-3,5-dicarboxylates **4a**–**i** and **4l-o** were prepared according to the literature.^43^

**B**: 4-Substituted-1,4-dihydropyridine-3,5-dicarbonitriles **4j** and **4k** were prepared according to the literature.^44^

#### Diethyl 4-benzyl-2,6-dimethyl-1,4-dihydropyridine-3,5-dicarboxylate (4a)

Synthesized following general procedure **A** using 1.16 mL (10 mmol, 1.0 equiv) of phenylacetaldehyde in 6 h. Pure **4a** was obtained using flash chromatography on silica gel (hexane:ethyl acetate 8:2 to 7:3) in 34% yield (1.17 g, 3.4 mmol) as a pale-yellow powder. ^1^H NMR (400 MHz, CDCl_3_) δ 7.21–7.09 (m, 3H), 7.07–6.96 (m, 2H), 5.36 (s, 1H), 4.19 (t, *J* = 5.5 Hz, 1H), 4.12–3.97 (m, 4H), 2.58 (d, *J* = 5.5 Hz, 2H), 2.17 (s, 6H), 1.23 (t, *J* = 7.1 Hz, 6H) ppm. ^13^C{^1^H} NMR (101 MHz, CDCl_3_) δ 167.9, 145.4, 139.4, 130.2, 127.4, 125.7, 102.0, 59.7, 42.4, 35.6, 19.3, 14.5 ppm. *These data matched with those previously reported in the literature.*(45)

#### Diethyl 2,6-dimethyl-4-((2-phenylcyclopropyl)methyl)-1,4-dihydropyridine-3,5-dicarboxylate (4b)

Synthesized following general procedure **A** using 420 mg (2.6 mmol, 1.0 equiv) of 2-(2-phenylcyclopropyl)acetaldehyde (**S12**) in 4 h. Pure **4b** was obtained using flash chromatography on silica gel (hexane:ethyl acetate 9:1) in 33% yield (329 mg, 0.86 mmol) as a pale-yellow powder. ^1^H NMR (400 MHz, CDCl_3_) δ 7.20 (t, *J* = 7.7 Hz, 2H), 7.08 (t, *J* = 7.3 Hz, 1H), 6.93 (d, *J* = 6.8 Hz, 2H), 5.33 (s, 1H), 4.16–4.01 (m, 5H), 2.23 (s, 3H), 2.09 (s, 3H), 1.59 (dt, *J* = 13.9, 5.4 Hz, 1H), 1.49 (dt, *J* = 8.9, 4.7 Hz, 1H), 1.31 (dd, *J* = 8.3, 5.5 Hz, 1H), 1.28–1.21 (m, 6H), 1.10–0.97 (m, 1H), 0.78–0.64 (m, 2H) ppm. ^13^C{^1^H} NMR (101 MHz, CDCl_3_) δ 168.1, 168.0, 145.2, 145.0, 144.6, 128.2, 125.4, 125.1, 103.2, 102.8, 59.7, 40.9, 33.6, 23.9, 20.7, 19.64, 19.60, 16.5, 14.6 ppm. HRMS (ESI-MS) calculated for C_23_H_28_NO_4_^–^ [M–H]^−^ 382.2024, found 382.2046.

#### Diethyl 4-(4-bromobenzyl)-2,6-dimethyl-1,4-dihydropyridine-3,5-dicarboxylate (4c)

Synthesized following general procedure **A** using 468 mg (2.4 mmol, 1.0 equiv) of 2-(4-bromophenyl)acetaldehyde (**S1**) in 6 h. Pure **4c** was obtained using flash chromatography on silica gel (hexane:ethyl acetate 9:1 to 8:2) in 27% yield (277 mg, 0.66 mmol) as a pale-yellow powder. ^1^H NMR (400 MHz, CDCl_3_) δ 7.29 (d, *J* = 8.1 Hz, 2H), 6.89 (d, *J* = 8.1 Hz, 2H), 5.59 (s, 1H), 4.17 (t, *J* = 5.2 Hz, 1H), 4.15–4.00 (m, 4H), 2.53 (d, *J* = 5.2 Hz, 2H), 2.17 (s, 6H), 1.25 (t, *J* = 7.1 Hz, 6H) ppm. ^13^C{^1^H} NMR (101 MHz, CDCl_3_) δ 167.8, 145.8, 138.4, 131.9, 130.3, 119.7, 101.5, 59.8, 41.7, 35.4, 19.3, 14.5 ppm. *These data matched with those previously reported in the literature.*(43)

#### Diethyl 4-(3-chlorobenzyl)-2,6-dimethyl-1,4-dihydropyridine-3,5-dicarboxylate (4d)

Synthesized general procedure **A** using 375 mg (2.4 mmol, 1.0 equiv) of 2-(3-chlorophenyl)acetaldehyde (**S2**) in 6 h. Pure **4d** was obtained using flash chromatography on silica gel (hexane:ethyl acetate 9:1 to 8:2) in 37% yield (337 mg, 0.89 mmol) as a pale-yellow powder. ^1^H NMR (400 MHz, CDCl_3_) δ 7.15–7.01 (m, 3H), 6.87 (dt, *J* = 7.0, 1.6 Hz, 1H), 5.41 (s, 1H), 4.19 (t, *J* = 5.4 Hz, 1H), 4.15–4.00 (m, 4H), 2.55 (d, *J* = 5.4 Hz, 2H), 2.18 (s, 6H), 1.25 (t, *J* = 7.1 Hz, 6H) ppm. ^13^C{^1^H} NMR (101 MHz, CDCl_3_) δ 167.8, 145.8, 141.6, 133.2, 130.3, 128.6, 128.5, 125.9, 101.7, 59.9, 42.1, 35.6, 19.4, 14.5 ppm. HRMS (ESI-MS) calculated for C_20_H_23_ClNO_4_^–^ [M–H]^−^ 376,1321, found 372.1322.

#### Diethyl 2,6-dimethyl-4-(4-(trifluoromethoxy)benzyl)-1,4-dihydropyridine-3,5-dicarboxylate (4e)

Synthesized following general procedure **A** using 204 mg (2.0 mmol, 1.0 equiv) of 2-(4-(trifluoromethoxy)phenyl)acetaldehyde (**S3**) in 6 h. Pure **4e** was obtained using flash chromatography on silica gel (hexane:ethyl acetate 9:1 to 85:15) in 39% yield (335 mg, 0.78 mmol) as a pale-yellow powder. ^1^H NMR (600 MHz, CDCl_3_) δ 7.03 (s, 4H), 5.29 (s, 1H), 4.18 (t, *J* = 5.6 Hz, 1H), 4.14–3.95 (m, 4H), 2.58 (d, *J* = 5.6 Hz, 2H), 2.18 (s, 5H), 1.23 (t, *J* = 7.1 Hz, 6H) ppm. ^13^C{^1^H} NMR (151 MHz, CDCl_3_) δ 167.6, 147.6 (q, *J* = 1.6 Hz), 145.4, 138.3, 131.2, 120.5 (q, *J* = 254.9 Hz), 119.9, 101.8, 59.7, 41.6, 35.5, 19.3, 14.3 ppm. ^19^F NMR (188 MHz, CDCl_3_) δ −58.38 (s, 3F) ppm. HRMS (ESI-MS) calculated for C_21_H_23_F_3_NO_5_^–^ [M–H]^−^ 426.1534, found 426.1563.

#### Diethyl 4-(2,4-dimethylbenzyl)-2,6-dimethyl-1,4-dihydropyridine-3,5-dicarboxylate (4f)

Synthesized following general procedure **A** using 1 g (6.7 mmol, 1.0 equiv) of 2-(2,4-dimethylphenyl)acetaldehyde in 5 h. Pure **4f** was obtained using flash chromatography on silica gel (hexane:ethyl acetate 8:2) in 34% yield (842 mg, 2.3 mmol) as a pale-yellow powder. ^1^H NMR (400 MHz, CDCl_3_) δ 6.89 (s, 1H), 6.84–6.74 (m, 3H), 5.80 (s, 1H), 4.20 (t, *J* = 7.0 Hz, 1H), 4.03–3.79 (m, 4H), 2.54 (d, *J* = 7.0 Hz, 2H), 2.34 (s, 3H), 2.29 (s, 6H), 2.24 (s, 3H), 1.17 (t, *J* = 7.1 Hz, 3H) ppm. ^13^C{^1^H} NMR (101 MHz, CDCl_3_) δ 168.0, 145.2, 137.1, 135.3, 134.1, 131.2, 130.6, 125.8, 103.0, 59.7, 39.3, 33.7, 21.0, 19.5, 19.4, 14.3 ppm. HRMS (ESI-MS) calculated for C_22_H_28_NO_4_^–^ [M–H]^−^ 370.2024, found 370.2011.

#### Diethyl 2,6-dimethyl-4-(1-phenylethyl)-1,4-dihydropyridine-3,5-dicarboxylate (4g)

Synthesized following general procedure **A** using 1.33 mL (10 mmol, 1.0 equiv) of 2-phenylpropanal in 4 h. Pure **4g** was obtained using flash chromatography on silica gel (hexane:ethyl acetate 9:1 to 7:3) in 35% yield (1.25 g, 3.5 mmol) as a pale-yellow powder. ^1^H NMR (400 MHz, CDCl_3_) δ δ 7.19–6.95 (m, 5H), 5.28 (s, 1H), 4.18 (d, *J* = 5.1 Hz, 1H), 4.03–3.88 (m, 3H), 3.79 (m, 1H), 2.66 (qd, *J* = 7.2, 4.9 Hz, 1H), 2.10 (s, 6H), 1.18 (t, *J* = 7.1 Hz, 4H), 1.13–1.02 (m, 6H) ppm. ^13^C{^1^H} NMR (101 MHz, CDCl_3_) δ 168.74, 168.71, 145.5, 145.2, 144.5, 128.8, 127.5, 126.1, 101.4, 101.3, 59.92, 59.89, 46.3, 19.6, 19.5, 15.8, 14.7, 14.6 ppm. *These data matched with those previously reported in the literature*.^45^

#### Diethyl 4-(1-(4-methoxyphenyl)ethyl)-2,6-dimethyl-1,4-dihydropyridine-3,5-dicarboxylate (4h)

Synthesized following general procedure **A** using 1.4 g (8.6 mmol, 1.0 equiv) of 2-(4-methoxyphenyl)propanal (**S8**) in 4 h. Pure **4h** was obtained using flash chromatography on silica gel (hexane:ethyl acetate 8:2) in 35% yield (1.16 g, 3.0 mmol) as a pale-yellow powder. ^1^H NMR (400 MHz, CDCl_3_) δ 6.99 (d, *J* = 8.6 Hz, 2H), 6.73 (d, *J* = 8.6 Hz, 2H), 5.48 (s, 1H), 4.22 (d, *J* = 5.0 Hz, 1H), 4.11–3.88 (m, 4H), 3.75 (s, 3H), 2.69 (qd, *J* = 7.2, 4.8 Hz, 1H), 2.19–2.16 (m, 6H), 1.27 (t, *J* = 7.1 Hz, 3H), 1.20 (t, J = 7.1 Hz, 3H), 1.13 (d, *J* = 7.3 Hz, 3H) ppm. ^13^C{^1^H} NMR (101 MHz, CDCl_3_) δ 168.6, 168.5, 158.0, 145.3, 145.0, 136.4, 129.5, 112.7, 101.2, 101.0, 59.73, 59.68, 55.4, 45.3, 40.3, 19.4, 19.2, 15.9, 14.5, 14.4 ppm. HRMS (ESI-MS) calculated for C_22_H_28_NO_5_^–^ [M–H]^−^ 386.1973, found 386.1976.

#### Diethyl 2,6-dimethyl-4-(1-phenylpropyl)-1,4-dihydropyridine-3,5-dicarboxylate (4i)

Synthesized following general procedure **A** using 352 mg (2.4 mmol, 1.0 equiv) of 2-phenylbutanal (**S4**) in 6 h. Pure **4i** was obtained using flash chromatography on silica gel (hexane:ethyl acetate 9:1) in 52% yield (462 mg, 1.2 mmol) as a pale-yellow powder. ^1^H NMR (400 MHz, CDCl_3_) δ 7.19–7.08 (m, 3H), 7.03–6.96 (m, 2H), 5.13 (s, 1H), 4.33 (d, *J* = 4.6 Hz, 1H), 4.18–4.00 (m, 4H), 2.42 (dt, *J* = 10.1, 5.2 Hz, 1H), 2.13 (s, 3H), 2.10 (s, 3H), 1.75–1.53 (m, 2H), 1.27 (td, *J* = 7.1, 4.8 Hz, 6H), 0.76 (t, *J* = 7.3 Hz, 3H) ppm. ^13^C{^1^H} NMR (101 MHz, CDCl_3_) δ 169.0, 168.5, 145.6, 145.5, 142.5, 129.6, 127.2, 126.1, 101.6, 100.9, 59.92, 59.88, 55.0, 39.0, 23.3, 19.5, 14.69, 14.66, 12.9 ppm. *These data matched with those previously reported in the literature*.^43^

#### 2,6-Dimethyl-4-(1,2,3,4-tetrahydronaphthalen-1-yl)-1,4-dihydropyridine-3,5-dicarbonitrile (4j)

Synthesized following general procedure **B** using 625 mg (3.9 mmol, 1.0 equiv) of 1,2,3,4-tetrahydronaphthalene-1-carbaldehyde (**S9**) in 4 h. Pure **4j** was obtained using flash chromatography on silica gel (hexane:ethyl acetate 6:4 to 4:6) in 12% yield (137 mg, 0.47 mmol) as a pale-yellow powder. ^1^H NMR (400 MHz, CDCl_3_) δ 7.25–7.20 (m, 1H), 7.20–7.12 (m, 2H), 7.12–7.06 (m, 1H), 5.91 (s, 1H), 3.86 (d, J = 3.7 Hz, 1H), 3.17 (q, J = 7.1, 6.5 Hz, 1H), 2.82–2.68 (m, 2H), 2.11 (s, 3H), 2.05 (s, 3H), 1.99–1.89 (m, 2H), 1.82–1.64 (m, 1H) ppm. ^13^C{^1^H} NMR (101 MHz, CDCl_3_) δ 147.5, 146.9, 139.0, 135.1, 129.3, 128.8, 126.5, 125.9, 118.7, 117.8, 84.4, 82.7, 43.5, 42.1, 30.2, 24.9, 21.4, 18.72, 18.66 ppm. HRMS (ESI-MS) calculated for C_19_H_18_N_3_^–^ [M–H]^−^ 288.1506, found 288.1513.

#### 2,6-Dimethyl-4-(2-phenylpropan-2-yl)-1,4-dihydropyridine-3,5-dicarbonitrile (4k)

Synthesized following general procedure **B** using 1.08 g (7.3 mmol, 1.0 equiv) of 2-methyl-2-phenylpropanal (**S10**) in 4 h. Pure **4k** was obtained using flash chromatography on silica gel (hexane:ethyl acetate 6:4 to 4:6) in 45% yield (910 mg, 3.3 mmol) as a pale-yellow powder. ^1^H NMR (400 MHz, CDCl_3_) δ 7.26–7.17 (m, 5H), 6.23 (s, 1H), 3.22 (s, 1H), 1.95 (s, 6H), 1.35 (s, 6H) ppm. ^13^C{^1^H} NMR (101 MHz, CDCl_3_) δ 148.6, 144.2, 127.8, 127.0, 126.7, 119.7, 81.0, 47.6, 46.6, 24.6, 18.3 ppm. *These data matched with those previously reported in the literature*.^44^

#### Diethyl 2,6-dimethyl-4-(naphthalen-2-ylmethyl)-1,4-dihydropyridine-3,5-dicarboxylate (4l)

Synthesized following general procedure **A** using 391 mg (2.3 mmol, 1.0 equiv) of 2-(naphthalen-2-yl)acetaldehyde (**S5**) in 5 h. Pure **4l** was obtained using flash chromatography on silica gel (hexane:ethyl acetate 75:25) in 34% yield (307 mg, 0.78 mmol) as a pale-yellow powder. ^1^H NMR (400 MHz, CDCl_3_) δ 7.80–7.70 (m, 2H), 7.65 (d, *J* = 8.4 Hz, 1H), 7.47–7.34 (m, 3H), 7.20 (dd, *J* = 8.4, 1.7 Hz, 1H), 5.19 (s, 1H), 4.28 (t, *J* = 5.3 Hz, 1H), 4.12–3.91 (m, 4H), 2.75 (d, *J* = 5.3 Hz, 2H), 2.12 (s, 6H), 1.19 (t, *J* = 7.1 Hz, 6H) ppm. ^13^C{^1^H} NMR (101 MHz, CDCl_3_) δ 168.0, 145.6, 137.1, 133.4, 132.1, 129.3, 128.4, 127.62, 127.60, 126.5, 125.7, 125.1, 101.9, 59.7, 42.6, 35.8, 19.3, 14.5 ppm. HRMS (ESI-MS) calculated for C_24_H_26_NO_4_^–^ [M–H]^−^ 392.1867, found 392.1870.

#### Diethyl 2,6-dimethyl-4-(naphthalen-1-ylmethyl)-1,4-dihydropyridine-3,5-dicarboxylate (4m)

Synthesized following general procedure **A** using 306 mg (1.8 mmol, 1.0 equiv) of 2-(naphthalen-1-yl)acetaldehyde (**S6**) in 5 h. Pure **4m** was obtained using flash chromatography on silica gel (hexane:ethyl acetate 75:25) in 29% yield (205 mg, 0.52 mmol) as a pale-yellow powder. ^1^H NMR (400 MHz, CDCl_3_) δ 8.38 (d, 1H, *J* = 8.4 Hz), 7.80 (d, 1H, *J* = 7.6 Hz), 7.65 (d, 1H, *J* = 7.6 Hz), 7.51–7.44 (m, 2H), 7.30–7.26 (m, 2H), 7.04 (d, 1H, *J* = 6.8 Hz), 5.80 (s, 1H), 4.43 (t, 1H, *J* = 6.7 Hz), 3.93–3.85 (m, 2H), 3.65–3.57 (m, 2H), 3.02 (d, 2H, *J* = 6.7 Hz), 2.24 (s, 6H), 0.93 (t, 3H, *J* = 7.2 Hz) ppm. ^13^C{^1^H} NMR (101 MHz, CDCl_3_) δ 167.9, 145.4, 135.2, 133.7, 133.2, 128.3, 128.1, 126.6, 125.4, 125.3, 125.1, 124.9, 102.8, 59.6, 39.8, 34.2, 19.4, 13.8 ppm. *These data matched with those previously reported in the literature*.^46^

#### Diethyl 2,6-dimethyl-4-(thiophen-3-ylmethyl)-1,4-dihydropyridine-3,5-dicarboxylate (4n)

Synthesized following general procedure **A** using 306 mg (2.4 mmol, 1.0 equiv) of 2-(thiophen-3-yl)acetaldehyde (**S7**) in 6 h. Pure **4n** was obtained using flash chromatography on silica gel (hexane:ethyl acetate 75:25) in 34% yield (289 mg, 0.83 mmol) as a pale-yellow powder. ^1^H NMR (600 MHz, CDCl_3_) δ 7.10 (dd, *J* = 4.9, 3.0 Hz, 1H), 6.81 (dd, *J* = 4.9, 1.2 Hz, 1H), 6.76 (d, *J* = 2.8 Hz, 1H), 5.34 (s, 1H), 4.16 (t, *J* = 5.4 Hz, 1H), 4.14–4.05 (m, 4H), 2.61 (d, *J* = 5.4 Hz, 2H), 2.18 (s, 6H), 1.26 (t, *J* = 7.1 Hz, 6H) ppm. ^13^C{^1^H} NMR (151 MHz, CDCl_3_) δ 168.0, 145.4, 139.7, 130.1, 123.7, 122.0, 102.1, 59.8, 36.6, 35.2, 19.5, 14.6 ppm. HRMS (ESI-MS) calculated for C_18_H_22_NO_4_S^–^ [M–H]^−^ 348.1275, found 348.1310.

#### Diethyl 2,6-dimethyl-4-(1-(naphthalen-2-yl)ethyl)-1,4-dihydropyridine-3,5-dicarboxylate (4o)

Synthesized following general procedure **A** using 563 mg (3.1 mmol, 1.0 equiv) of 2-(naphthalen-2-yl)propanal (**S11**) in 5 h. Pure **4o** was obtained using flash chromatography on silica gel (hexane:ethyl acetate 75:25) in 37% yield (465 mg, 1.1 mmol) as a pale-yellow powder. ^1^H NMR (400 MHz, CDCl_3_) δ 7.75 (m, 2H), 7.67 (d, *J* = 8.5 Hz, 1H), 7.48 (as, 1H), 7.43–7.35 (m, 2H), 7.31 (dd, *J* = 8.5, 1.8 Hz, 1H), 5.24 (s, 1H), 4.37 (d, *J* = 4.7 Hz, 1H), 4.03 (q, *J* = 7.1 Hz, 2H), 3.92 (dq, *J* = 10.8, 7.1 Hz, 1H), 3.72 (dq, *J* = 10.8, 7.1 Hz, 1H), 2.94 (m, 1H), 2.16 (s, 3H), 2.14 (s, 3H), 1.29–1.20 (m, 6H), 1.08 (t, *J* = 7.1 Hz, 3H) ppm. ^13^C{^1^H} NMR (101 MHz, CDCl_3_) δ 168.7, 168.6, 145.7, 145.3, 142.0, 133.4, 132.5, 128.0, 127.9, 127.7, 126.9, 126.6, 125.8, 125.3, 101.3, 101.1, 59.9, 59.9, 46.5, 40.5, 19.6, 19.4, 15.8, 14.6, 14.4 ppm. *These data matched with those previously reported in the literature.*(43)

### General Procedure C for the Synthesis of BF_3_K and BF_3_NBu_4_ Salts

BF_3_K salts were synthesized following a reported procedure.^47^ BF_3_NBu_4_ salts were prepared from the corresponding potassium salts by ion-exchange according to the literature procedures.^48^ The yield of this step was quantitative.

#### Tetrabutyl ammonium benzyltrifluoroborate (28a)

Synthesized following general procedure **C** using 357 μL (3.0 mmol, 1.0 equiv) of benzyl bromide. Pure **28a** was obtained in 72% yield (883 mg, 2.2 mmol) as a white solid. ^1^H NMR (400 MHz, Acetone-*d*_6_) δ 7.12–7.10 (m, 2H), 7.06–7.02 (m, 2H), 6.88 (1H, br t, *J* = 7.3 Hz), 1.65 (s, 2H) ppm. ^13^C{^1^H} NMR (101 MHz, Acetone-*d*_6_) δ 148.0, 129.7, 127.8, 122.9, 30.4, 30.0 ppm. *These data matched with those previously reported in the literature*.^49^

#### Tetrabutyl ammonium 4-methylbenzyltrifluoroborate (28b)

Synthesized following general procedure **C** using 555 mg (3.0 mmol, 1.0 equiv) 4-methylbenzyl bromide. Pure **28b** was obtained in 70% yield (872 mg, 2.1 mmol) as a white solid. ^1^H NMR (400 MHz, Acetone-*d*_6_) δ 6.98 (d, *J* = 7.6 Hz, 2H), 6.85 (d, *J* = 7.5 Hz, 2H), 2.19 (s, 3H), 1.58 (bs, 2H) ppm. ^13^C{^1^H} NMR (101 MHz, Acetone-*d*_6_) δ 144.7, 131.5, 129.7, 128.6, 21.0 ppm. *These data matched with those previously reported in the literature*.^50^

#### Potassium 2-methoxybenzyltrifluoroborate (28c)

Synthesized following general procedure **C** using 420 μL (3.0 mmol, 1.0 equiv) of 3-methoxylbenzyl bromide. Pure **28c** was obtained in 70% yield (479 mg, 2.1 mmol) as a white solid. ^1^H NMR (400 MHz, Acetone-*d*_6_) δ 6.93 (t, *J* = 7.8 Hz, 1H), 6.70–6.60 (m, 2H), 6.45 (dd, *J* = 8.0, 2.6 Hz, 1H), 3.69 (s, 3H), 1.61 (s, 2H) ppm. ^13^C{^1^H} NMR (101 MHz, Acetone-*d*_6_) δ 160.0, 149.7, 128.5, 122.4, 115.4, 108.5, 55.0 ppm. *These data matched with those previously reported in the literature*.^50^

### General Procedure D for Photochemical Carboxylation Reaction

In a 4 mL vial, DHPs **4a**–**4o** or trifluoroborate salt **28a**–**c** (0.1 mmol, 1.0 equiv), photocatalyst (0.002 mmol, 2 mol %), and TBD (0.15 mmol, 2 equiv) were added, and then the vial was closed with a PTFE/silicone septum cap and degassed with CO_2_. The reagents were dissolved in CO_2_-degassed acetonitrile (2 mL, 0.05 M) and the reaction mixture was bubbled with CO_2_ for 30 s. Then, the vial was placed in the photochemical reactor shown in section **A.2** of the Supporting Information and irradiated for 15 h at 20 °C. NMR yield was measured using 14 μL of CH_2_Br_2_ and 25 μL of acetic acid. The solvent was removed, the product was moved in a separating funnel using 10 mL of hexane:DCM 9:1, and 10 mL of a NaOH 1 M aqueous solution were added. The two phases were separated, and then the aqueous phase was washed 2 more times with hexane:DCM 9:1. The reunited aqueous phase was acidified adding a HCl 2 M aqueous solution dropwise until pH 2 is reached. The acidified aqueous phase was extracted with ethyl acetate (5 × 15 mL). The organic phases were collected, dried over anhydrous MgSO_4_, filtered, and concentrated under reduced pressure, yielding the pure product.

#### 2-Phenylacetic acid (10)

Synthesized following general procedure **D** starting from 34.3 mg (0.1 mmol, 1.0 equiv) of **4a**, using **11** as photocatalyst. Pure **10** was obtained in 76% NMR and 63% isolated yield (8.6 mg, 0.063 mmol) as a white solid. ^1^H NMR (400 MHz, CDCl_3_) δ 7.37–7.15 (m, 5H), 3.61 (s, 2H) ppm. ^13^C{^1^H} NMR (101 MHz, CDCl_3_) δ 178.1, 133.2, 129.3, 128.6, 127.3, 41.1 ppm. *These data matched with those previously reported in the literature*.^51^

### Scale-up Synthesis of 2-Phenylacetic Acid (**10**) in 1 mmol Scale

In a 50 mL Schlenk tube, 343 mg (1.0 mmol, 1.0 equiv) of **4a**, 4CzIPN **11** (15.8 mg, 0.02 mmol, 2 mol %), and TBD (209 mg, 1.5 mmol, 2 equiv) were added. The Schlenk tube was subjected to 3 vacuum/CO_2_ cycles, and then the reagents were dissolved in CO_2_-degassed acetonitrile (20 mL, 0.05 M), and the reaction mixture was bubbled with CO_2_ for 30 s. Then, the Schlenk tube was wrapped with an LED strip and placed in the photochemical reactor shown in section **A.2** of the Supporting Information and irradiated for 15 h at 20 °C. The solvent was removed under reduced pressure, the product was moved in a separating funnel using 80 mL of ethyl hexane:DCM 9:1, and 120 mL of a NaOH 1 M aqueous solution were added. The two phases were separated, and then the aqueous phase was further washed with hexane:DCM 9:1 (2 × 80 mL). The reunited aqueous phase was acidified adding a HCl 2 M aqueous solution (100 mL). The acidified aqueous phase was extracted with ethyl acetate (5 × 50 mL). The organic phases were collected, dried over anhydrous MgSO_4_, filtered, and concentrated under reduced pressure, giving the pure product **10** in 75% yield (103 mg, 0.75 mmol) as a pale orange solid.

#### 2-(4-Bromophenyl)acetic acid (14)

Synthesized following general procedure **D** starting from 42.2 mg (0.1 mmol, 1.0 equiv) of **4c**, using **11** as photocatalyst. Pure **14** was obtained in 65% NMR and 52% isolated yield (11.2 mg, 0.052 mmol) as a white solid. ^1^H NMR (400 MHz, CDCl_3_) δ 7.46 (d, *J* = 8.0 Hz, 2H), 7.16 (d, *J* = 8.0 Hz, 2H), 3.61 (s, 2H) ppm. ^13^C{^1^H} NMR (101 MHz, CDCl_3_) δ 176.9, 132.1, 131.8, 131.1, 121.5, 40.3 ppm. *These data matched with those previously reported in the literature*.^52,53^

#### 2-(2-Chlorophenyl)acetic acid (15)

Synthesized following general procedure **D** starting from 37.7 mg (0.1 mmol, 1.0 equiv) of **4d**, using **11** as photocatalyst. **15** was obtained in 40% ^1^H NMR yield, as judged by integration of the diagnostic benzylic peak at 3.47 ppm.^54^

#### 2-(4-(Trifluoromethoxy)phenyl)acetic acid (16)

Synthesized following general procedure **D** starting from 42.7 mg (0.1 mmol, 1.0 equiv) of **4e**, using **11** as photocatalyst. **16** was obtained in 59% ^1^H NMR yield, as judged by integration of the diagnostic benzylic peak at 3.50 ppm.^55^

#### 2-(2,4-Dimethylphenyl)acetic acid (17)

Synthesized following general procedure **D** starting from 37.1 mg (0.1 mmol, 1.0 equiv) of **4f**, using **11** as photocatalyst. Pure **17** was obtained in 63% NMR and 54% isolated yield (8.9 mg, 0.054 mmol) as a white solid. ^1^H NMR (600 MHz, CDCl_3_) δ 7.08 (d, *J* = 7.7 Hz, 1H), 7.01 (s, 1H), 6.98 (d, *J* = 7.6 Hz, 1H), 3.63 (s, 2H), 2.30 (s, 3H), 2.28 (s, 3H) ppm. ^13^C{^1^H} NMR (151 MHz, CDCl_3_) δ 177.5, 137.5, 136.9, 131.5, 130.4, 129.2, 127.1, 38.6, 21.2, 19.7 ppm. HRMS (ESI-MS) calculated for C_10_H_11_O_2_^–^ [M–H]^−^ 163.0765, found 163.0748.

#### 2-Phenylpropanoic acid (18)

Synthesized following general procedure **D** starting from 35.7 mg (0.1 mmol, 1.0 equiv) of **4g**, using **11** as photocatalyst. Pure **18** was obtained in 68% NMR and 58% isolated yield (8.7 mg, 0.058 mmol) as a colorless oil. ^1^H NMR (400 MHz, CDCl_3_) δ 7.41–7.29 (m, 5H), 3.75 (q, *J* = 7.2 Hz, 1H), 1.52 (d, *J* = 7.2 Hz, 3H) ppm. ^13^C{^1^H} NMR (101 MHz, CDCl_3_) δ 181.0, 139.9, 128.8, 127.7, 127.5, 45.5, 18.2 ppm. *These data matched with those previously reported in the literature*.^26^

#### 2-(4-Methoxyphenyl)propanoic acid (19)

Synthesized following general procedure **D** starting from 38.7 mg (0.1 mmol, 1.0 equiv) of **4h**, using **13** as photocatalyst. Pure **19** was obtained in 47% isolated yield (8.4 mg, 0.047 mmol) as a white solid. ^1^H NMR (400 MHz, CDCl_3_) δ 7.22 (d, *J* = 8.9 Hz, 2H), 6.85 (d, *J* = 8.7 Hz, 2H), 3.77 (s, 3H), 3.67 (q, *J* = 7.2 Hz, 1H), 1.47 (d, *J* = 7.2 Hz, 3H) ppm. ^13^C{^1^H} NMR (101 MHz, CDCl_3_) δ 180.8, 159.0, 132.0, 128.8, 114.2, 55.4, 44.6, 18.3 ppm. *These data matched with those previously reported in the literature*.^26^

#### 2-Phenylbutanoic acid (20)

Synthesized following general procedure **D** starting from 37.1 mg (0.1 mmol, 1.0 equiv) of **4i**, using **11** as photocatalyst. **20** was obtained in 51% ^1^H NMR yield, as judged by integration of the diagnostic benzylic peak at 3.34 ppm.^54^

#### 1,2,3,4-Tetrahydronaphthalene-1-carboxylic acid (21)

Synthesized following general procedure **D** starting from 28.9 mg (0.1 mmol, 1.0 equiv) of **4j**, using **11** as photocatalyst. Pure **21** was obtained in 37% NMR and 36% isolated yield (6.3 mg, 0.036 mmol) as a pale-yellow oil. ^1^H NMR (400 MHz, CDCl_3_) δ 7.28–7.08 (m, 4H), 3.86 (t, *J* = 5.7 Hz, 1H), 2.91–2.71 (m, 2H), 2.28–2.15 (m, 1H), 2.11–1.91 (m, 2H), 1.87–1.74 (m, 1H) ppm. ^13^C{^1^H} NMR (101 MHz, CDCl_3_) δ 181.3, 137.3, 132.6, 129.6, 129.5, 127.1, 125.8, 44.5, 29.1, 26.5, 20.4 ppm. *These data matched with those previously reported in the literature*.^56,57^

#### 2-Methyl-2-phenylpropanoic acid (22)

Synthesized following general procedure **D** starting from 27.7 mg (0.1 mmol, 1.0 equiv) of **4k**, using **13** as photocatalyst. Pure **22** was obtained in 43% isolated yield (7.1 mg, 0.043 mmol) as a white solid. ^1^H NMR (400 MHz, CDCl_3_) δ 7.44–7.37 (m, 2H), 7.39–7.30 (m, 2H), 7.30–7.22 (m, 1H), 1.61 (s, 6H) ppm. ^13^C{^1^H} NMR (101 MHz, CDCl_3_) δ 181.9, 142.9, 127.4, 125.9, 124.8, 45.3, 26.2 ppm. *These data matched with those previously reported in the literature.*(58)

#### 2-(Naphthalen-2-yl)acetic acid (23)

Synthesized following general procedure **D** starting from 39.3 mg (0.1 mmol, 1.0 equiv) of **4l**, using **11** as photocatalyst. Pure **23** was obtained in 48% NMR and 42% isolated yield (7.8 mg, 0.042 mmol) as a yellow solid. ^1^H NMR (400 MHz, CDCl_3_) δ 7.91–7.77 (m, 3H), 7.74 (s, 1H), 7.54–7.36 (m, 3H), 3.82 (s, 2H) ppm. ^13^C{^1^H} NMR (101 MHz, CDCl_3_) δ 177.4, 133.4, 132.6, 130.7, 128.3, 128.2, 127.73, 127.68, 127.3, 126.2, 125.9, 41.1 ppm. *These data matched with those previously reported in the literature*.^51^

#### 2-(Naphthalen-1-yl)acetic acid (24)

Synthesized following general procedure **D** starting from 39.3 mg (0.1 mmol, 1.0 equiv) of **4m**, using **11** as photocatalyst. Pure **24** was obtained in 58% NMR and 49% isolated yield (9.1 mg, 0.049 mmol) as a yellow solid. ^1^H NMR (400 MHz, CDCl_3_) δ 7.97 (brd, *J* = 7.5 Hz, 1H), 7.87 (dd, *J* = 7.2, 2.4 Hz, 1H), 7.81 (dd, *J* = 6.9, 2.6 Hz, 1H), 7.58–7.38 (m, 4H), 4.09 (s, 2H) ppm. ^13^C{^1^H} NMR (101 MHz, CDCl_3_) δ 178.2, 133.9, 132.1, 129.9, 128.9, 128.5, 128.3, 126.6, 126.0, 125.6, 123.8, 38.9 ppm. *These data matched with those previously reported in the literature*.^59^

#### 2-(Thiophen-3-yl)acetic acid (25)

Synthesized following general procedure **D** starting from 34.9 mg (0.1 mmol, 1.0 equiv) of **4n**, using **13** as photocatalyst. Pure **25** was obtained in 40% isolated yield (5.7 mg, 0.040 mmol) as a pale-yellow solid. ^1^H NMR (400 MHz, CDCl_3_) δ 7.31 (dd, *J* = 5.0, 3.0 Hz, 1H), 7.18 (d, *J* = 1.6 Hz, 1H), 7.05 (dd, *J* = 4.9, 1.3 Hz, 1H), 3.71 (s, 2H) ppm. ^13^C{^1^H} NMR (101 MHz, CDCl_3_) δ 176.9, 132.9, 128.6, 126.1, 123.5, 35.6 ppm. HRMS (ESI-MS) calculated for C_6_H_5_O_2_S^–^ [M–H]^−^ 141.0016, found 141.0018.

#### 2-(Naphthalen-2-yl)propanoic acid (26)

Synthesized following general procedure **D** starting from 40.8 mg (0.1 mmol, 1.0 equiv) of **4o**, using **11** as photocatalyst. Pure **26** was obtained in 60% NMR and 57% isolated yield (11.4 mg, 0.057 mmol) as a yellow solid. ^1^H NMR (400 MHz, CDCl_3_) δ 7.90–7.69 (m, 4H), 7.55–7.37 (m, 3H), 3.92 (q, *J* = 7.1 Hz, 1H), 1.61 (d, *J* = 7.1 Hz, 3H) ppm. ^13^C{^1^H} NMR (101 MHz, CDCl_3_) δ 180.5, 137.4, 133.6, 132.9, 128.6, 128.0, 127.8, 126.6, 126.4, 126.1, 125.9, 45.6, 18.3 ppm. *These data matched with those previously reported in the literature*.^60^

#### 2-Phenylpent-4-enoic acid (27)

Synthesized following general procedure **D** starting from 38.3 mg (0.1 mmol, 1.0 equiv) of **4b**, using **11** as photocatalyst. Pure **27** was obtained in 37% isolated yield (6.5 mg, 0.037 mmol) as a yellow oil. ^1^H NMR (400 MHz, CDCl_3_) δ 7.37–7.23 (m, 5H), 5.79–5.65 (m, 1H), 5.12–5.06 (m, 2H), 5.02 (d, *J* = 10.2 Hz, 1H), 3.66 (dd, *J* = 8.4, 7.1 Hz, 1H), 2.83 (ddd, *J* = 14.1, 8.4, 7.2 Hz, 1H), 2.53 (dt, *J* = 13.9, 6.9 Hz, 1H) ppm. ^13^C{^1^H} NMR (101 MHz, CDCl_3_) δ 179.7, 137.9, 134.9, 128.7, 128.2, 127.6, 117.3, 51.4, 37.1 ppm. *These data matched with those previously reported in the literature*.^61^

#### 2-(*p*-Tolyl)acetic acid (29)

Synthesized following general procedure **D** starting from 41.5 mg (0.1 mmol, 1.0 equiv) of **28b**, using **11** as photocatalyst. After workup the crude was further purified by flash column chromatography (petroleum ether:EtOAc, 7:3 + 1% AcOH) to get pure **29** in 43% isolated yield (6.5 mg, 0.043 mmol) as a white solid. ^1^H NMR (400 MHz, CDCl_3_) δ 7.21–7.10 (m, 4H), 3.61 (s, 2H), 2.33 (s, 3H) ppm. ^13^C{^1^H} NMR (101 MHz, CDCl_3_) δ 178.5, 137.1, 130.3, 129.5, 129.4, 40.8, 21.2 ppm. *These data matched with those previously reported in the literature*.^59^

#### 2-(3-Methoxyphenyl)acetic acid (30)

Synthesized following general procedure **D** starting from 22.8 mg (0.1 mmol, 1.0 equiv) of **28c**, using **11** as photocatalyst. Pure **30** was obtained in 71% NMR and 70% isolated yield (11.6 mg, 0.070 mmol) as a yellow solid. ^1^H NMR (400 MHz, CDCl_3_) δ 7.28–7.21 (m, 1H), 6.90–6.80 (m, 3H), 3.80 (s, 3H), 3.63 (s, 2H) ppm. ^13^C{^1^H} NMR (101 MHz, CDCl_3_) δ 177.7, 159.7, 134.7, 129.6, 121.7, 115.1, 112.9, 55.2, 41.1 ppm. *These data matched with those previously reported in the literature*.^62^

### Procedures and Characterizations for Product Manipulation

#### Benzyl benzylcarbamate (31)

The procedure was adapted from a report in literature.^63^ 68.1 mg of **10** (0.5 mmol, 1 equiv) were dissolved in 2 mL of toluene, 119 μL of diphenylphosphoryl azide (0.55 mmol, 1.1 equiv), and 77 μL triethylamine (0.55 mmol, 1.1 equiv) were added. The mixture was refluxed, using an oil bath, for 2 h under N_2_. Gas release was observed. The reaction mixture was cooled to 60 °C, and 62 μL of benzyl alcohol (0.6 mmol, 1.2 equiv) were added in one portion. The mixture was heated to 80 °C, using an oil bath, for 2 days. After cooling to room temperature, 40 mL of water were added, and the mixture was extracted with ethyl acetate (3 × 30 mL). The combined organic phases were washed with water (2 × 30 mL) and once with brine, dried over anhydrous MgSO_4_, filtered, and concentrated under reduced pressure. The residual oil was purified by column chromatography on silica gel using toluene:ethyl acetate 98:2 to 95:5 as eluent mixture, giving **31** in 78% yield (93.5 mg, 0.39 mmol) as a white solid. ^1^H NMR (400 MHz, CDCl_3_) δ 7.42–7.26 (m, 10H), 5.14 (s, 2H), 5.07 (s, 1H), 4.39 (d, *J* = 5.9 Hz, 2H) ppm. ^13^C{^1^H} NMR (101 MHz, CDCl_3_) δ 156.6, 138.5, 136.6, 128.8, 128.7, 128.3, 127.7, 67.0, 45.3 ppm. *These data matched with those previously reported in the literature.*(64)

#### (1*R*,2*S*,5*R*)-2-Isopropyl-5-methylcyclohexyl 2-phenylacetate (32)

Synthesized following a reported procedure,^65^ starting from 13.6 mg of **10** (0.1 mmol, 1.0 equiv) Pure **32** was obtained using flash chromatography (hexane:ethyl acetate 8:2 to 7:3) in 97% yield (26.6 mg, 0.097 mmol) as a pale-yellow oil. ^1^H NMR (400 MHz, CDCl_3_) δ 7.37–7.26 (m, 5H), 4.71 (td, *J* = 11.2, 4.0 Hz, 1H), 3.63 (s, 2H), 2.03–1.94 (m, 1H), 1.80–1.74 (m, 1H), 1.73–1.65 (m, 2H), 1.54–1.33 (m, 2H), 1.11–1.01 (m, 1H), 1.00–0.89 (m, 2H), 0.94–0.86 (m, *J* = 6.8 Hz, (6H), 0.72 (d, *J* = 6.8 Hz, 3H). ppm. ^13^C{^1^H} NMR (101 MHz, CDCl_3_) δ 171.2, 134.4, 129.2, 128.5, 126.9, 74.7, 47.1, 41.9, 40.8, 34.3, 31.4, 26.1, 23.4, 22.0, 20.7, 16.3 ppm. *These data matched with those previously reported in the literature.*(65)

#### 1,2-Diphenylpropan-1-one (33)

Synthesized following a procedure from a literature report,^66^ starting from 26.7 mg of 18 (0.18 mmol, 1.0 equiv) Pure **33** was obtained using flash chromatography (hexane:ethyl acetate 95:5) as a colorless oil in 91% yield (24.5 mg, 0.12 mmol, 66% over 2 steps). ^1^H NMR (400 MHz, CDCl_3_) δ 7.25 (t, *J* = 7.4 Hz, 2H), 7.19–7.13 (m, 3H), 3.68 (q, *J* = 7.0 Hz, 1H), 1.95 (s, 3H), 1.32 (d, *J* = 7.1 Hz, 3H) ppm. ^13^C{^1^H} NMR (101 MHz, CDCl_3_) δ 208.3, 140.7, 128.9, 127.8, 127.1, 53.6, 28.2, 17.2 ppm. *These data matched with those previously reported in the literature.*(66)

## Data Availability

The data underlying this study are available in the published article and its Supporting Information.

## Supporting Information Available

Details on the materials, instrumentation, synthetic and characterization procedures (PDF)
