# Supplementary material for: A Proton-Coupled Electron Transfer Strategy to the Redox-Neutral Photocatalytic CO2 Fixation
Source: J Org Chem. 2023 Feb 10;88(10):6454–64. doi: 10.1021/acs.joc.2c02952 (PMC10204093; doi:10.1021/acs.joc.2c02952)

Supporting Information

A Proton-Coupled Electron Transfer Strategy to the Redox-Neutral Photocatalytic CO_2 Fixation

Pietro Franceschi, Elena Rossin, Giulio Goti, Angelo Scopano, Alberto Vega-Peñaloza, Mirco Natali, Deepak Singh, Andrea Sartorel,* and Luca Dell'Amico*

Department of Chemical Sciences, University of Padova, Via Marzolo 1, 35131 Padova, Italy

Department of chemical, pharmaceutical, and agricultural sciences, University of Ferrara, Via L. Borsari 46, 44121 – Ferrara, Italy

E-mail: andrea.sartorel@unipd.it

luca.dellamico@unipd.it

TABLE OF CONTENTS

A. GENERAL INFORMATION 4

A.1. LIGHT SOURCES EMISSION SPECTRA 7

A.2. PHOTOREACTOR SETUP 9

B. REACTION OPTIMIZATION 10

B.1. PCS SCREENING 10

B.2. BASES SCREENING 12

B.3. SOLVENT AND CONCENTRATION SCREENING 13

B.4. PC LOADING SCREENING 14

B.5. LIGHT SOURCE SCREENING 15

B.6. REACTION TIME SCREENING 16

B.7. TEMPERATURE SCREENING 17

B. 8. CONTROL EXPERIMENTS 18

B.9. UNREACTIVE SUBSTRATES 19

C. GENERAL PROCEDURES FOR THE SYNTHESIS OF THE STARTING MATERIALS 20

C.1. PREPARATION OF ALDEHYDES S1-S7 AS SYNTHETIC PRECURSORS 20

C.2. PREPARATION OF ALDEHYDE S8 AS SYNTHETIC PRECURSOR 23

C.3. PREPARATION OF ALDEHYDE S9 AS SYNTHETIC PRECURSOR 24

C.4. PREPARATION OF ALDEHYDE S10 AS SYNTHETIC PRECURSOR 25

C.5. PREPARATION OF ALDEHYDE S11 AS SYNTHETIC PRECURSOR 26

C.6. PREPARATION OF ALDEHYDE S12 AS SYNTHETIC PRECURSOR 27

C.7. PREPARATION OF 3-AMINOBUT-2-ENENITRILE AS SYNTHETIC PRECURSOR 30

D. ELECTROCHEMICAL MEASUREMENTS 31

E. SPECTROSCOPIC DATA 33

F. DFT CALCULATIONS **37**

G. ^{1}H-NMR ANALYSIS **38**

H. REFERENCES **40**

I. NMR SPECTRA **42**

A. General Information

435 nm LEDs were purchased on Amazon (https://www.amazon.it/Striscia-Tesfish-Larghezza-Strisce-Decorazione/dp/B09439PFYH/ref=sr_1_3?__mk_it_IT=%C3%85M%C3%85%C5%BD%C3%95%C3%91&crid=1FX05NAFDZC41&keywords=blue%2Bled%2B12%2BV&qid=1667899334&sprefix=blue%2Bled%2B12%2Caps%2C478&sr=8-3&th=1), as well as the waterproof silicone tubing used for submerging the LEDs under water (https://www.amazon.it/Silicone-Guaine-Flessibile-BobinaStriscia/dp/B00TOM6NWO/ref=sr_1_4?__mk_it_IT=%C3%85M%C3%85%C5%BD%C3%95%C3%91&dchild=1&keywords=tubo+silicone+led&qid=1622812658&s=lighting&sr=1-4).

Chromatographic purification of products was accomplished using flash chromatography on silica gel (SiO_2, 0.04-0.063 mm) purchased from Machery-Nagel, with the indicated solvent system according to the standard techniques. Thin-layer chromatography (TLC) analysis was performed on pre-coated Merck TLC plates (silica gel 60 GF254, 0.25 mm). Visualization of the developed chromatography was performed by checking UV absorbance (254 nm and 365 nm) as well as with phosphomolybdic acid and potassium permanganate solutions. Organic solutions were concentrated under reduced pressure on a Büchi rotary evaporator.

NMR spectra were recorded on a Bruker Avance 300 spectrometer equipped with a BBO-z grad probehead, a Bruker 400 AVANCE III HD equipped with a BBI-z grad probehead, and a Bruker AVANCE Neo 600 equipped with a TCI Prodigy cryoprobe. The chemical shifts (δ) for ^{1}H and ^{13}C are given in ppm relative to residual signals of the solvents ($CHCl_3$ @ 7.26 ppm ^{1}H NMR, 77.2 ppm ^{13}C NMR; acetone @ 2.05 ppm ^{1}H NMR, 29.84 ppm ^{13}C NMR). Coupling constants are given in Hz. The following abbreviations are used to indicate the multiplicity: s, singlet; d, doublet; t, triplet; q, quartet; m, multiplet; bs, broad signal; qd, quartet of doublets; brs, broad singlet; brd, broad doublet; brt, broad triplet. NMR yields were calculated by using dibromomethane (4.95 ppm, s, 2H) as internal standard.

High-Resolution Mass Spectra (HRMS) were obtained using Waters GCT gas chromatograph coupled with a time-of-flight mass spectrometer (GC/MS-TOF) with electron ionization (EI).

Steady-state absorption spectroscopy studies have been performed at room temperature on a Varian Cary 50 UV-Vis double beam spectrophotometer; 10 mm path length Hellma Analytics 100 QS quartz cuvettes have been used.

Nanosecond time-resolved emission measurements were performed with an Applied Photophysics laser flash photolysis apparatus, using a frequency-doubled (532 nm, 330 mJ) or tripled (355 nm, 160 mJ) Surelite Continuum II Nd/YAG laser (half-width 6-8 ns) as

excitation source. Detection was obtained using a photomultiplier-oscilloscope combination (Hamamatsu R928, LeCroy 9360).

IR measurements were carried out at room temperature on a JASCO FT/IR-4100 spectrophotometer; 1 mm path length Hellma Analytics 100 QX quartz cuvettes have been used.

The electrochemical characterizations were carried out at room temperature, on a BASi EC Epsilon potentiostat-galvanostat. A typical three-electrode cell was employed, which was composed of glassy carbon (GC) working electrode (3 mm diameter), a platinum wire as counter electrode and a silver/silver chloride electrode (Ag/AgCl (NaCl 3 M)) as reference electrode. The reference electrode is a silver wire that is coated with a thin layer of silver chloride; the electrode body contains sodium chloride (NaCl 3 M). The GC electrode was polished before any measurement with diamond paste and ultrasonically rinsed with deionized water for 15 minutes.

The authors are grateful to the research support area at Department of Chemical Sciences (DiSC) of the University of Padova.

Materials: Commercial grade reagents and solvents were purchased at the highest commercial quality from Sigma Aldrich or FluoroChem and used as received, unless otherwise stated. Carbon dioxide 99.5% cylinder was purchased from ForHome (https://www.forhome.it/Bombola-Co2-da-4Kg-Acciaio-Ricarcabile-Nuova-Con-Valvola-Residuale-Certificata-per-sistemi-di-gasatura-acqua) and 99.99% cylinder from SAPIO (https://www.sapio.it/). Cs_2CO_3 and ethyl 3-aminobut-2-enoate were purchased from FluoroChem. All the remaining bases used in Table S.2., and all the remaining reagent used in the synthesis of substrates **4a**-**4o, 28a**-**c** and synthetic precursors **S1**-**S12** were purchased from Sigma-Aldrich.

The photocatalysts used in Table S.1 were prepared following literature procedures.[1]

A.1. LIGHT SOURCES EMISSION SPECTRA

The following spectra were recorded by an AvaSpec ULS3648 high-resolution fiber-optic spectrometer which was placed at a fixed distance of 0.5 cm from the light source.

(more info at: https://www.avantes.com/products/spectrometers/starline/item/209-avaspec-uls3648-high-resolution-spectrometer).

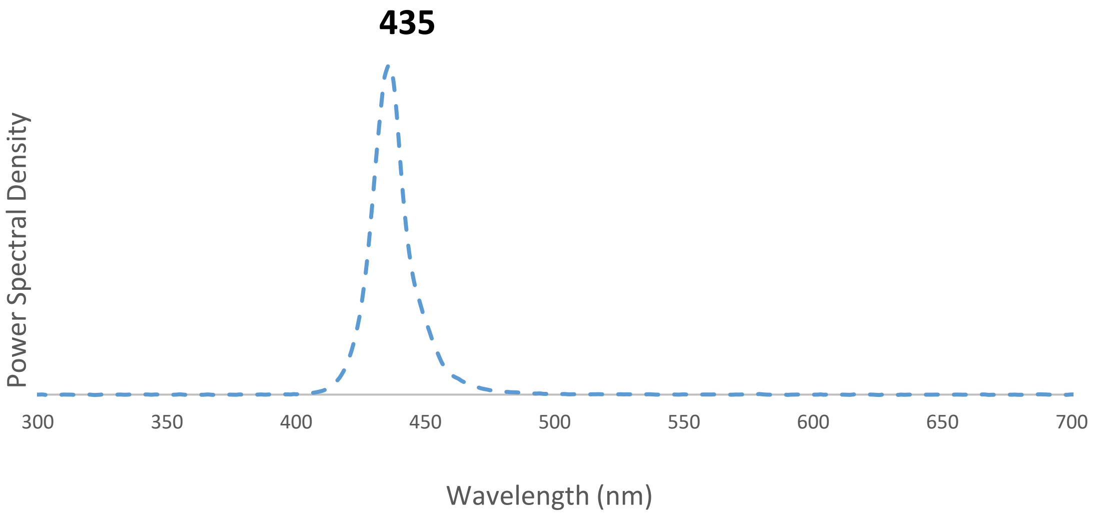

Figure S1. Emission spectra of the 435nm LED strip used in this work.

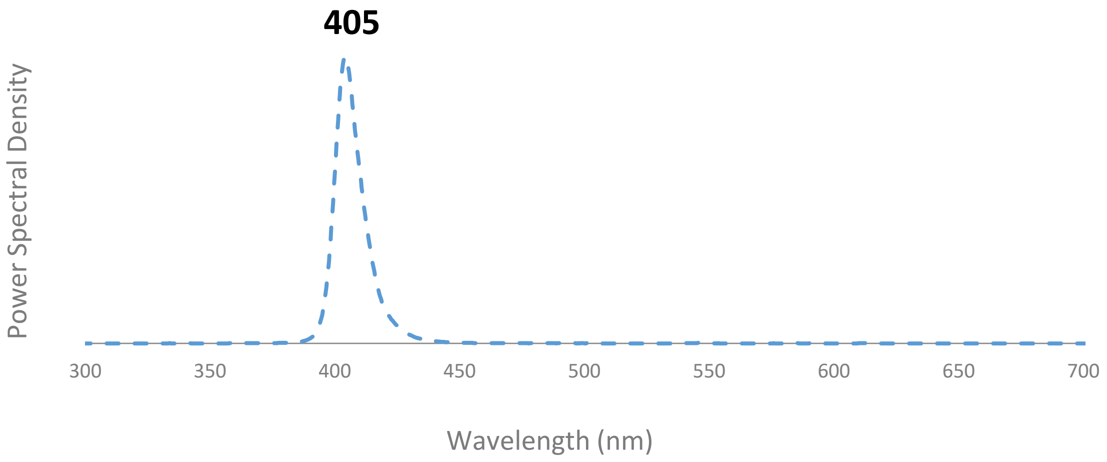

Figure S2. Emission spectra of the 20 W 405 nm LED lamp used in this work.

The following spectrum is reported in the Kessil website (http://www.kessil.com/science/PR160L.php).

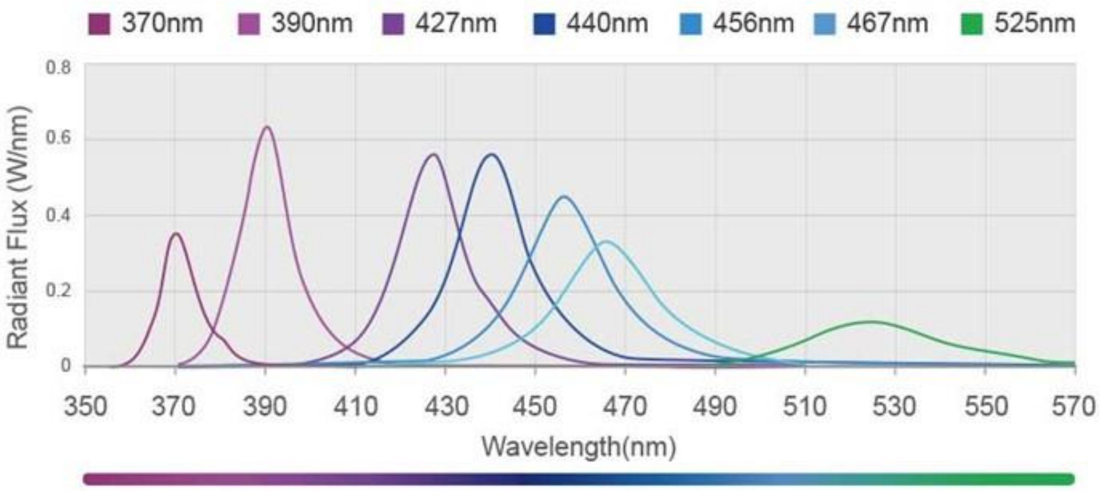

Figure S3. Emission spectra of the 427nm, and other Kessil lamps used in this work.

A.2. PHOTOREACTOR SETUP

The experiments have been carried out in 4 mL vials equipped with PTFE/silicone septum caps, both purchased from Sigma Aldrich, or in a 50 mL Schlenk tube. Whether three vials, or the Shlenk tube are wrapped together using LED strip covered with a rubber waterproof case, as illustrated in Figure S4, and immersed in a thermostat water-bath to maintain a stable reaction temperature (20±2°C) as depicted in Figure S5. For safety and contamination reason, during the experiments, the photoreactor was enveloped with Aluminum sheets. The reactions were stirred vigorously using a stirring plate.

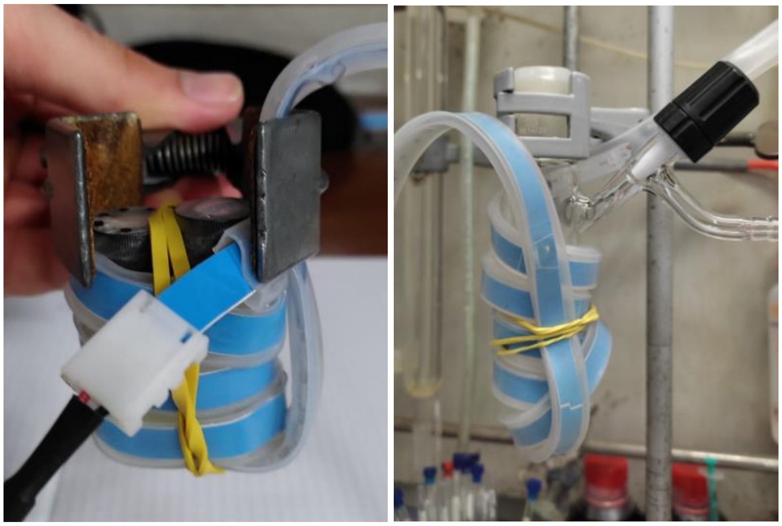

Figure S4. Core of the photoreactor.

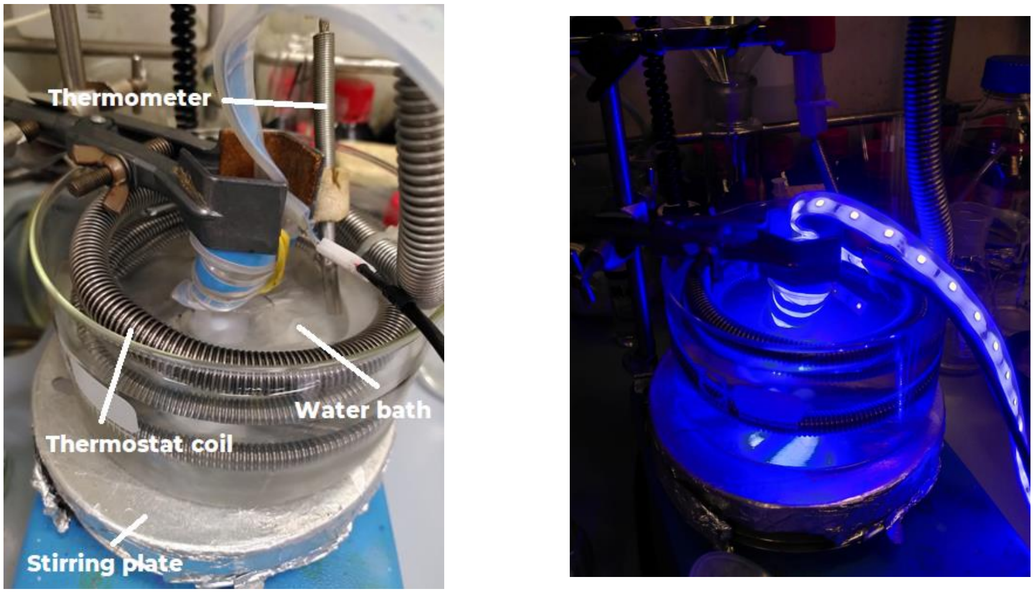

Figure S5. Photoreactor setup.

B. REACTION OPTIMIZATION

B.1. PCS SCREENING

Table S1. General reaction conditions: 0.1 mmol of substrate, DMF 1 mL, Cs_2CO_3 1.1 equiv., PC 10 mol%, CO_2 1 atm, irradiation with 435nm LEDs at 20°C for 6 h. Yields were calculated by NMR analysis with dibromomethane as internal standard. a. 405 nm LED lamp was used.

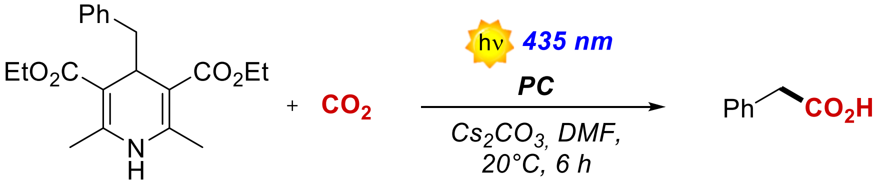

entry	PC	yield
1	4CzIPN	50%
2	4CzBnBN	52%
3[a]	NC-3OH	2% (20% conversion)
4[a]	phenoxazine	16%
5	3DPAFIPN	49%
6	3DPA2FBN	46%

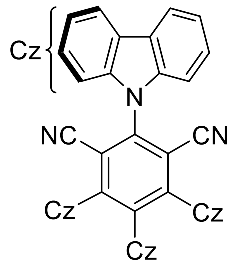

4CzIPN:
2,4,5,6-Tetrakis(9H-carbazol-9-yl) isophthalonitrile

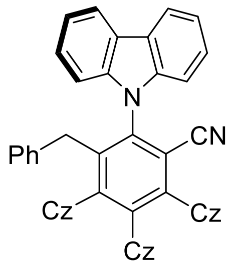

4CzBnBN:
(2,3,4,6)-3-benzyl-2,4,5,6-tetra (9H-carbazol-9-yl)benzonitrile

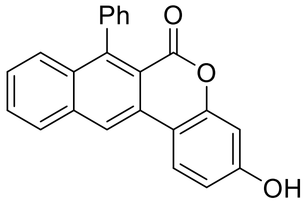

NC-3OH:
3-hydroxy-7-phenyl-6*H*-naphtho[2,3-*c*]chromen-6-one

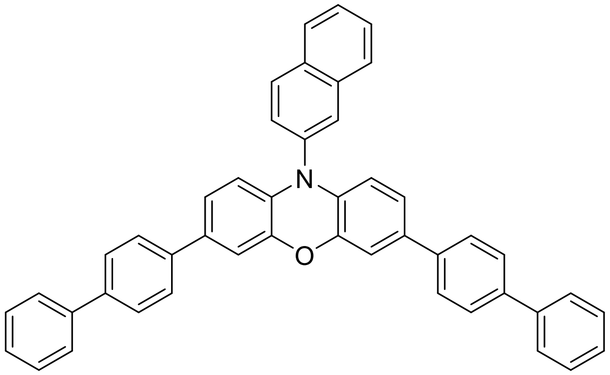

phenoxazine:
3,7-di([1,1'-biphenyl]-4-yl)-10-(naphthalen-2-yl)-10*H*-phenoxazine

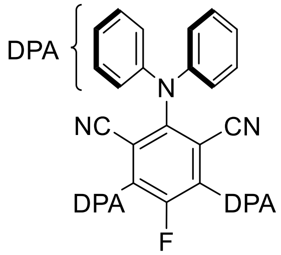

3DPAFIPN:
2,4,6-Tris(diphenylamino)-5-fluoroisophthalonitrile

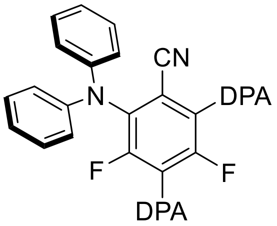

3DPA2FBN:
2,4,6-Tris(diphenylamino)-3,5-difluorobenzonitrile

B.2. Bases Screening

Table S2. General reaction conditions: 0.1 mmol of substrate, DMF 1 mL, base 1.5 equiv., 4CzIPN 10 mol%, CO_2 1 atm, irradiation with 435nm LEDs at 20°C for 6 h. Yields were calculated by NMR analysis with dibromomethane as internal standard. a. 1.1 equiv. of base was used. b. 73% conversion. c. PC 2 mol%, MeCN 0.05M.

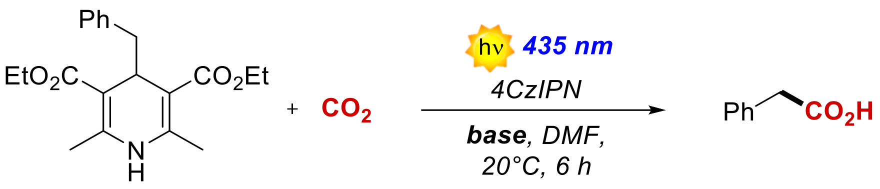

entry	base	yield
1[a]	Cs_2CO_3	50%
2	Cs_2CO_3	54%
3	K_2CO_3	50%
4[b]	NEt_3	49%
5	DBU	68%
6	TMG	51%
7	TBD	71%
8	Me-TBD	58%
9	DBN	54%
10[c]	DMAP	33%

B.3. SOLVENT AND CONCENTRATION SCREENING

Table S3. General reaction conditions: 0.05-0.1 mmol of substrate, TBD 1.5 equiv., 4CzIPN 10 mol%, CO_2 1 atm, irradiation with 435nm LEDs at 20°C for 6 h. Yields were calculated by NMR analysis with dibromomethane as internal standard.

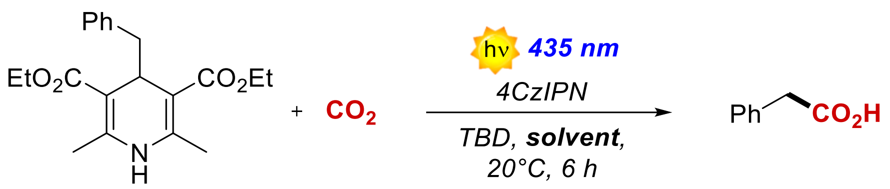

entry	solvent	concentration of substrate	yield
1	DMF	0.1 M	71%
2	MeCN	0.1 M	68%
3	DMSO	0.1 M	54%
4	DMAc	0.1 M	72%
5	DMAc	0.05 M	76%
6	MeCN	0.05 M	73%

MeCN was selected over DMAc because it is easier to evaporate and remove.

B.4. PC Loading Screening

Table S4. General reaction conditions: 0.1 mmol of substrate, MeCN 2 mL, TBD 1.5 equiv.,4CzIPN, CO_2 1 atm, irradiation with 435nm LEDs at 20°C for 6 h. Yields were calculated by NMR analysis with dibromomethane as internal standard. a. 76% conversion.

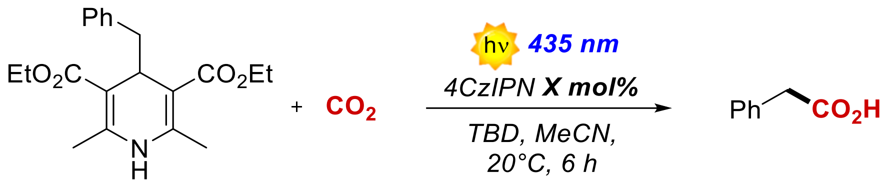

entry	PC loading	yield
1	10 mol%	73%
2	5 mol%	76%
3	2 mol%	76%
4[a]	1 mol%	48%

B.5. LIGHT SOURCE SCREENING

Table S5. General reaction conditions: 0.1 mmol of substrate, MeCN 2 mL, TBD 1.5 equiv.,4CzIPN 2 mol%, CO_2 1 atm, irradiation with 435nm LEDs or Kessil lamps at 25% power at 20°C for 3 h. Yields were calculated by NMR analysis with dibromomethane as internal standard. a. 49% conversion.

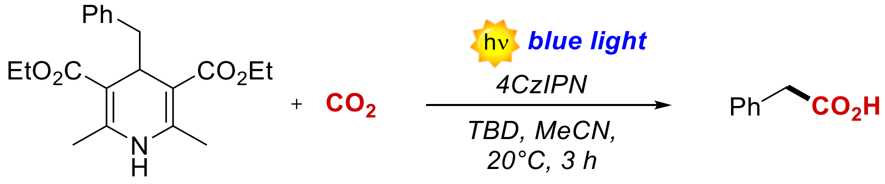

entry	light source	yield
1[a]	Kessil 456 nm	33%
2	Kessil 427 nm	76%
3	LED 435nm	76%

LED strips were selected as a cheaper and available light source.

B.6. REACTION TIME SCREENING

Table S6. General reaction conditions: 0.1 mmol of substrate, MeCN 2 mL, TBD 1.5 equiv.,4CzIPN 2 mol%, CO_2 1 atm, irradiation with 435nm LEDs at 20°C. Yields were calculated by NMR analysis with dibromomethane as internal standard. a. 60% conversion.

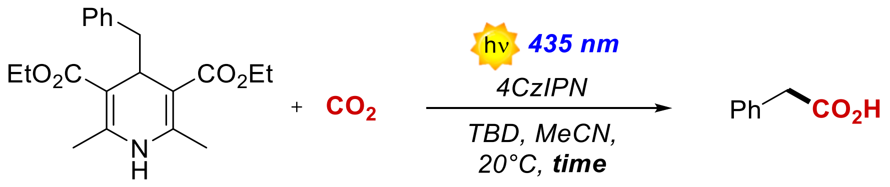

entry	reaction time	yield
1[a]	30 min	43%
2	1 h	73%
3	3 h	76%
4	6 h	76%
5	15 h	76%

Longer reaction time of 15 h was selected in order to observe full conversion of the starting material with all the other substrates, as no decrease in the yield was observed in the optimization.

B.7. TEMPERATURE SCREENING

Table S7. General reaction conditions: 0.1 mmol of substrate, MeCN 2 mL, TBD 1.5 equiv.,4CzIPN 2 mol%, CO_2 1 atm, irradiation with 435nm LEDs for 3 h. Yields were calculated by NMR analysis with dibromomethane as internal standard.

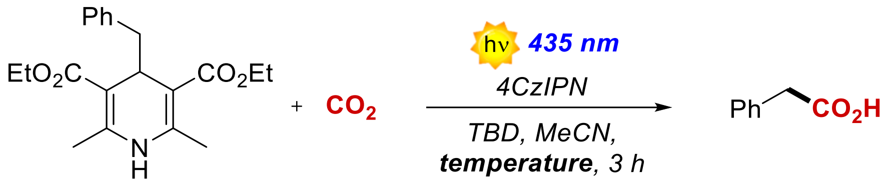

entry	temperature	yield
1	20°C	76%
2	50°C	55%

B. 8. CONTROL EXPERIMENTS

Table S8. General reaction conditions: 0.1 mmol of substrate, MeCN 2 mL, TBD 1.5 equiv., 4CzIPN 2 mol%, CO_2 1 atm, irradiation with 435nm LEDs at 20°C for 24 h. Yields were calculated by NMR analysis with dibromomethane as internal standard. a. Reaction time was 6 h.

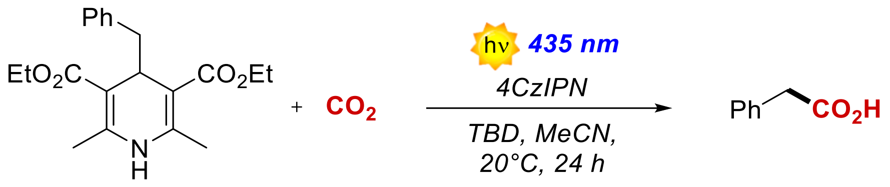

entry	change	yield	conversion
1	No CO_2	0%	100%
2	No light	0%	6%
3	No PC	1%	51%
4	No TBD	traces	100%
5[a]	0.3 equiv. TBD	43%	100%
6[a]	1 equiv. TBD	70%	100%

B.9. Unreactive substrates

The following list of substrates gave no product at all or gave poor reactivity.

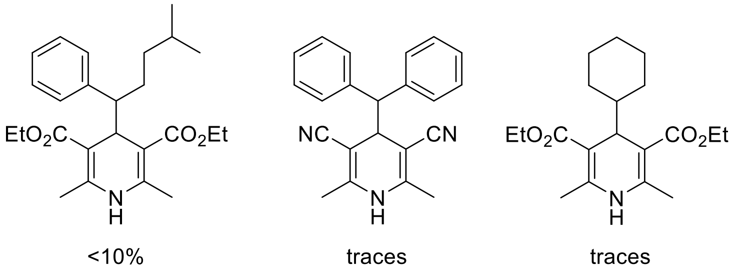

Figure S6. List of unreacted substrates tested in this work.

C. General Procedures For The Synthesis Of The Starting Materials

C.1. Preparation of aldehydes S1-S7 as synthetic precursors

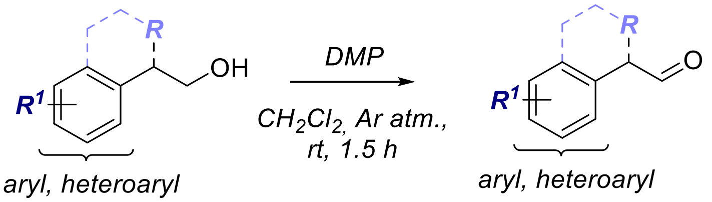

S1-S7

The corresponding alcohol (2.5 mmol, 1.0 equiv.) was added under Argon atmosphere to 20 mL of anhydrous dichloromethane in a flame-dried 100 mL round-bottom flask and left stirring at room temperature. Separately, 1.27 g of Dess-Martin periodinane (3 mmol, 1.2 equiv.) were added to 25 mL of anhydrous dichloromethane under Argon atmosphere in a flame-dried 50 mL round-bottom flask. The resulting mixture was stirred until a homogenous solution has been obtained, then it was added dropwisely to the solution of the alcohol. The final mixture was stirred for 1.5 hours at room temperature; precipitate formation was observed. The reaction was quenched by slow addition of saturated $NaHCO_3$ (aq), then the organic phase was collected and washed with a 6% (m/V) aqueous solution of NaS_2O_4 (3 x 50 mL). The resulting organic phase, obtained discarding the solid residuals, was dried over $MgSO_4$, filtered and concentrated under reduced pressure.

Crude aldehydes **S1-S7** were used in further synthetic steps without further purification.

2-(4-bromophenyl)acetaldehyde (S1)

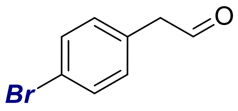

Synthesized from 0.35 mL (2.5 mmol, 1.0 equiv.) of 2-(4-bromophenyl)ethan-1-ol following the described procedure, yielding pure **S1** in 94% yield (468 mg, 2.4 mmol) as a colourless oil.

^{1}H NMR (400 MHz, CDCl$_3$): δ 9.74 (t, *J* = 2.1 Hz, 1H), 7.54 – 7.44 (m, 2H), 7.13 – 7.05 (m, 2H), 3.67 (d, *J* = 2.1 Hz, 2H) ppm. **^{13}C{^{1}H} NMR (101 MHz, CDCl$_3$):** δ 198., 132.1, 131.3, 130.8, 121.6, 49.9 ppm.

These data matched with the previously reported in literature.[2]

2-(3-chlorophenyl)acetaldehyde (S2)

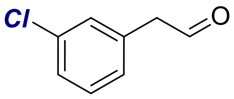

Synthesized from 0.33 mL (2.5 mmol, 1.0 equiv.) of 2-(3-chlorophenyl)ethan-1-ol following the described procedure, yielding pure **S2** in 97% yield (375 mg, 2.4 mmol) as a colourless oil.

^{1}H NMR (400 MHz, $CDCl_3$): δ 9.75 (t, *J* = 2.1 Hz, 1H), 7.32 – 7.28 (m, 2H), 7.22 (s, 1H), 7.13 – 7.08 (m, 1H), 3.69 (d, *J* = 2.1 Hz, 2H) ppm. **^{13}C{^{1}H} NMR (101 MHz, $CDCl_3$):** δ 198.4, 134.8, 133.7, 130.2, 129.7, 127.76, 127.7, 49.9 ppm.

These data matched with the previously reported in literature.[3]

2-(4-(trifluoromethoxy)phenyl)acetaldehyde (S3)

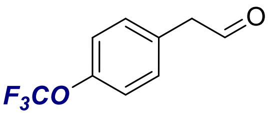

Synthesized from 0.40 mL (2.5 mmol, 1.0 equiv.) of 2-(4-(trifluoromethoxy)phenyl)ethan-1-ol following the described procedure, yielding pure **S3** in 79% yield (204 mg, 2.0 mmol) as a colourless oil.

^{1}H NMR (400 MHz, $CDCl_3$): δ 9.74 (d, *J* = 2.1 Hz, 1H), 7.16 – 7.24 (m, 4H), 3.69 (d, *J* = 2.1 Hz, 2H) ppm. **^{13}C{^{1}H} NMR (101 MHz, $CDCl_3$):** δ 198.6, 148.6, 131.0, 130.5, 121.5, 119.1, 49.7 ppm.

These data matched with the previously reported in literature.[4]

2-phenylbutanal (S4)

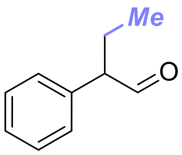

Synthesized from 0.39 mL (2.5 mmol, 1.0 equiv.) of 2-phenylbuthan-1-ol following the described procedure, yielding pure **S4** in 95% yield (363 mg, 2.4 mmol) as a colourless oil.

^{1}H NMR (400 MHz, $CDCl_3$): δ 9.68 (d, *J* = 2.1 Hz, 1H), 7.39 – 7.18 (m, 5H), 3.48 – 3.39 (m, 1H), 2.17 – 2.06 (m, 1H), 1.82 – 1.71 (m, 1H), 0.91 (t, *J* = 7.4 Hz, 3H) ppm. **^{13}C{^{1}H} NMR (101 MHz, $CDCl_3$):** δ 201.1, 136.4, 129.1, 128.9, 217.6, 61.0, 23.0, 11.8, ppm.

These data matched with the previously reported in literature.[5]

2-(naphthalen-2-yl)acetaldehyde (S5)

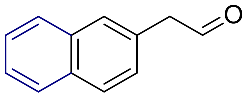

Synthesized from 430 mg (2.5 mmol, 1.0 equiv.) of 2-(naphthalen-2-yl)ethan-1-ol following the described procedure, yielding pure **S5** in 92% yield (391 mg, 2.3 mmol) as a pale-yellow oil.

^{1}H NMR (400 MHz, $CDCl_3$): δ 9.83 (t, *J* = 2.4 Hz, 1H), 7.87 – 7.81 (m, 3H), 7.70 (d, *J* = 1.8 Hz, 1H), 7.52 – 7.47 (m, 2H), 7,33 (dd, *J* = 8.4 , 1.8 Hz, 1H), 3.86 (d, *J* = 2.4 Hz, 2H) ppm. **^{13}C{^{1}H} NMR (101 MHz, $CDCl_3$):** δ 199.3, 133.6, 132.5, 129.3, 128.7, 128.5, 127.7, 127.6, 127.4, 126.4 126.0, 50.7 ppm.

These data matched with the previously reported in literature.[6]

2-(naphthalen-1-yl)acetaldehyde (S6)

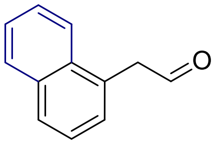

Synthesized from 430 mg (2.5 mmol, 1.0 equiv.) of 2-(naphthalen-1-yl)ethan-1-ol following the described procedure, yielding pure **S6** in 72% yield (306 mg, 1.8 mmol) as a pale-yellow oil.

^{1}H NMR (400 MHz, $CDCl_3$): δ 9.80 (t, *J* = 2.4 Hz, 1H), 7.95 − 7.83 (m, 3H), 7.61 − 7.41 (m, 4H), 4.12 (d, *J* = 2.4 Hz, 2H) ppm. **^{13}C{^{1}H} NMR (101 MHz, $CDCl_3$):** δ 199.5, 134.0, 133.4, 132.3, 128.9, 128.5, 128.4, 126.7, 126.1, 125.6, 123.5, 48.4 ppm.

These data matched with the previously reported in literature.[4]

2-(thiophen-3-yl)acetaldehyde (S7)

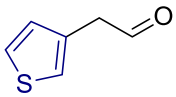

Synthesized from 0.28 mL (2.5 mmol, 1.0 equiv.) of 2-(thiophen-3-yl)ethan-1-ol following the described procedure, yielding pure **S7** in 97% yield (306 mg, 2.4 mmol) as a pale-yellow oil.

^{1}H NMR (400 MHz, $CDCl_3$): δ 9.74 (d, *J* = 2.3 Hz, 1H), 7.37 – 7.34 (m, 1H), 7.16 – 7.13 (m, 1H), 6.99 – 6.97 (m, 1H), 3.73 (d, *J* = 2.3 Hz, 2H) ppm. **^{13}C{^{1}H} NMR (101 MHz, $CDCl_3$):** δ 198.7, 131.5, 128.4, 126.5, 123.4, 44.8 ppm.

These data matched with the previously reported in literature.[4]

C.2. PREPARATION OF ALDEHYDE S8 AS SYNTHETIC PRECURSOR

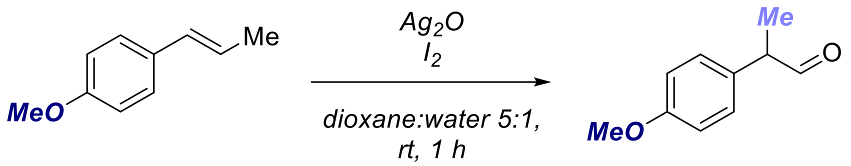

S8

S8 was synthesized following a procedure reported in literature.[7] 1.48 g of *trans*-anethol (10 mmol, 1 eq.) were dissolved in 60 ml mixture of 1,4-dioxane:water 5:1. 3.60 g of Ag_2O (15.5 mmol, 1.55 eq.) was added in one portion. To the vigorously stirred suspension, 3.93 g (15.5 mmol, 1.55 eq.) iodine was added portionwise during 5 min. The first purple and then red-coloured solution was stirred for an hour at room temperature, then it was filtered. The clear red solution was washed with 50 mL of saturated $Na_2S_2O_3$ (aq), with disappearance of the red colour, then extracted with diethyl ether (3 x 50 mL). The combined organic phases were dried over $MgSO_4$, filtered, and concentrated under reduced pressure yielding **S8** in 87% yield (1.43 g, 8.7 mmol) as a pale-yellow oil.

2-(4-methoxyphenyl)propanal (S8)

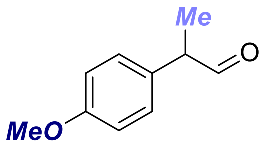

^{1}H NMR (400 MHz, $CDCl_3$): δ 9.65 (d, *J* = 1.5 Hz, 1H), 7.17 – 7.07 (m, 2H), 7.00 – 6.87 (m, 2H), 3.81 (s, 3H), 3.58 (qd, *J* = 7.0, 1.5 Hz, 1H), 1.42 (d, *J* = 7.0 Hz, 3H) ppm. **^{13}C{^{1}H} NMR (101 MHz, $CDCl_3$):** δ 201.3, 159.2, 123.7, 129.5, 114.6, 55.4, 52.3, 14.8 ppm.

These data matched with the previously reported in literature.[8]

C.3. PREPARATION OF ALDEHYDE S9 AS SYNTHETIC PRECURSOR

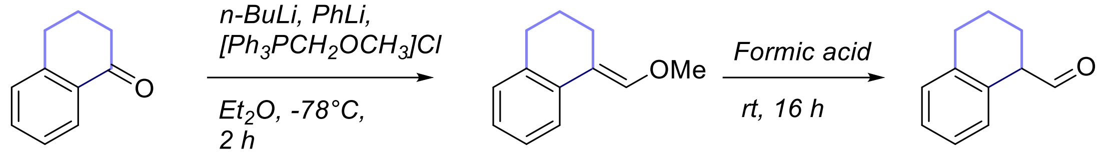

S9

S9 was prepared following a previously reported procedure.[7] To a suspension of 12.9 g of (methoxymethyl)triphenylphosphonium chloride (37.5 mmol, 1.5 eq.) in 50 ml diethyl ether under inert gas were added dropwise at -78 °C 7.75 ml of a 1.6 M solution of *n*-BuLi in hexane (9.9 mmol 0-4 equiv.) and then 16.5 ml of a 1.9 M PhLi solution in dibuthyl ether (31.5 mmol 1.25 equiv.). The yellow suspension was then stirred at 0 °C for another hour and became brown. The suspension was again cooled to -78 °C and a solution of 3.65 g of α-tetralone (25 mmol) in 18.75 ml of ether was added to the suspension. The mixture was stirred for 2 hours more at rt and became an orange suspension. The mixture was poured into 50 mL of saturated ice-cold NH_4Cl (aq) and extracted with ethyl acetate (3 x 50 mL). The reunited organic phases were washed once with water and once with brine. The organic phase was dried over $MgSO_4$, filtered, and concentrated under reduced pressure. The product was isolated by flash chromatography on silica gel (cyclohexane:ethyl acetate 5:1) in 54% yield (2.35 g, 13.5 mmol). Without further purification, this was dissolved in 15.7 ml of formic acid and stirred at room temperature for 16 hours resulting in a brown solution. Saturated $NaHCO_3$(aq) was added until pH = 8.2 was reached, then the mixture was extracted with ethyl acetate (3 x 50 mL). The collected organic phase was washed once with water and once with brine, dried over $MgSO_4$, filtered, and concentrated under reduced pressure. Product **S9** was isolated by silica gel flash chromatography (cyclohexane:ethyl acetate 50:1) in 29% (625 mg, 3.9 mmol) as a colourless oil.

1,2,3,4-tetrahydronaphthalene-1-carbaldehyde (S9)

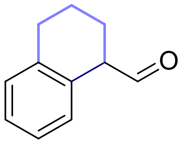

^{1}H NMR (400 MHz, $CDCl_3$): δ δ 9.68 (d, *J* = 2.0 Hz, 1H), 7.23 – 7.20 (m, 2H), 7.18 – 7.13 (m 2H), 3.61 – 3.58 (m, 1H), 2.79 (m, 2H), 2.31 – 2.17 (m, 1H), 1.99 – 1.87 (m, 1H), 1.84 – 1.78 (m, 2H) ppm. **^{13}C{^{1}H} NMR (101 MHz, $CDCl_3$):** δ 202.1, 138.0, 130.8, 129.8, 129.6, 127.1, 126.1, 51.7, 29.1, 23.0, 20.5 ppm.

These data matched with the previously reported in literature.[9]

C.4. PREPARATION OF ALDEHYDE S10 AS SYNTHETIC PRECURSOR

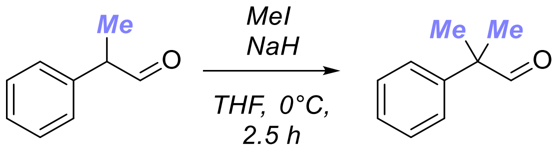

S10

S10 was prepared according to reported literature procedure.[10] 4.4 g of NaH 60% dispersion in paraffine oil (110 mmol, 1.1 equiv.) was suspended in 40 mL of anhydrous THF and the solution was cooled to 0°C. A solution of 13.4 mL of 2-phenylpropanal (100 mmol 1.0 equiv.) in 40 mL of THF was added dropwise over an hour and the suspension was stirred a further 30 minutes before 12.5 mL of methyl iodide (200 mmol, 2.0 equiv.) was carefully added dropwise, maintaining the temperature below 15°C. The reaction was stirred a further hour at 15°C before being brought to room temperature. The reaction was quenched by the addition of 200 mL saturated $NaHCO_3$(aq) and extracted with diethyl ether (3 x 50 mL). The organic phases were combined, dried over $MgSO_4$, filtered, and concentrated under reduced pressure. The crude material was purified by vacuum distillation to give **S10** in 48% yield (7.18 g, 48 mmol) as a yellow oil.

2-methyl-2-phenylpropanal (S10)

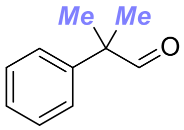

^{1}H NMR (400 MHz, $CDCl_3$): δ 9.50 (s, 1H), 7.28 –7.18 (m, 5H), 1.46 (s, 6H) ppm. **^{13}C{^{1}H} NMR (101 MHz, $CDCl_3$):** δ 202.2, 132.8, 128.9 (x2), 127.2 (x2), 126.7, 50.45, 22.5 (x2) ppm.

These data matched with the previously reported in literature.[11]

C.5. Preparation of aldehyde S11 as synthetic precursor

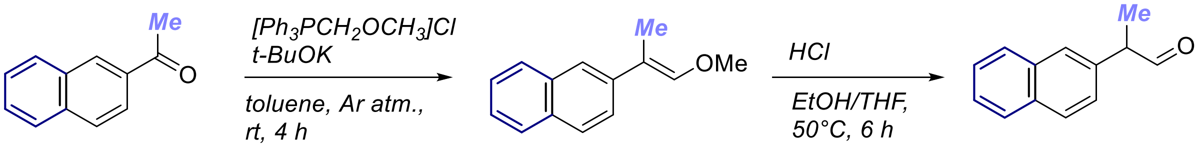

S11

S11 was obtained following a procedure reported in literature.[7] 1.25 g of 1-(naphthalen-2-yl)ethanone (7.4 mmol, 1.0 equiv.) was dissolved in 25 ml of toluene under argon atmosphere. 3.80 g of (methoxymethyl)triphenylphosphonium chloride (11.1 mmol, 1.5 equiv.) were added at room temperature, then 1.33 g of *t*-BuOK (11.8 mmol, 1.6 eq.) were added in four portions every 15 minutes to the suspension. The orange suspension was stirred until the mixture turned deep red and clear, with consumption of starting material, in 3 h. The solution was added dropwise to 100 ml water and stirred for 10 min, then extracted with ethyl acetate (3 x 50 mL). The organic layer was dried over $MgSO_4$, filtered, and concentrated under reduced pressure. 2-(1-methoxyprop-1-en-2-yl)naphthalene is obtained as a white solid by flash chromatography on silica gel (cyclohexane:ethyl acetate 4:1) in 80 % yield (1.17 g, 5.9 mmol). This was dissolved in 33 ml of THF, and 33 ml of 1M HCl solution were added dropwise in 4 portions every 10 min. Finally, 130 ml of ethanol were added to the solution, which was then heated to 50 °C, using an oil bath, under reflux condenser for 6 hours. After cooling to room temperature, solid $NaHCO_3$ was added to the colourless solution (with CO_2 development), until neutral pH value. The aqueous solution was extracted with ethyl acetate (3 x 50 mL) and the collected organic phase was washed once with water. The organic layer was dried over $MgSO_4$, filtered, and concentrated under reduced pressure. Pure **S11** was obtained by flash chromatography on silica gel (cyclohexane:ethyl acetate 4:1) in 52% yield (563 mg, 3.1 mmol) as white solid.

2-(naphthalen-2-yl)propanal (S11)

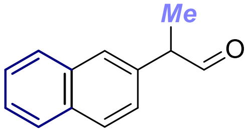

^{1}H NMR (400 MHz, $CDCl_3$): δ 9.76 (d, *J* = 1.0 Hz, 1H), 7.88 – 7.81 (m, 3H), 7.68 (s, 1H), 7.50 – 7.46 (m, 2H), 7.32 (dd, *J* = 6.8, 1.0 Hz, 1H), 3.80 (q, *J* = 6.8 Hz, 1H), 1.54 (d, *J* = 7.2 Hz, 3H) ppm. **^{13}C{^{1}H} NMR (101 MHz, $CDCl_3$):** δ 201.0, 135.1, 133.6, 132.6, 128.8, 127.7 (x2), 127.2, 126.4, 126.2, 126.1, 53.1, 14.6 ppm.

These data matched with the previously reported in literature.[12]

C.6. PREPARATION OF ALDEHYDE S12 AS SYNTHETIC PRECURSOR

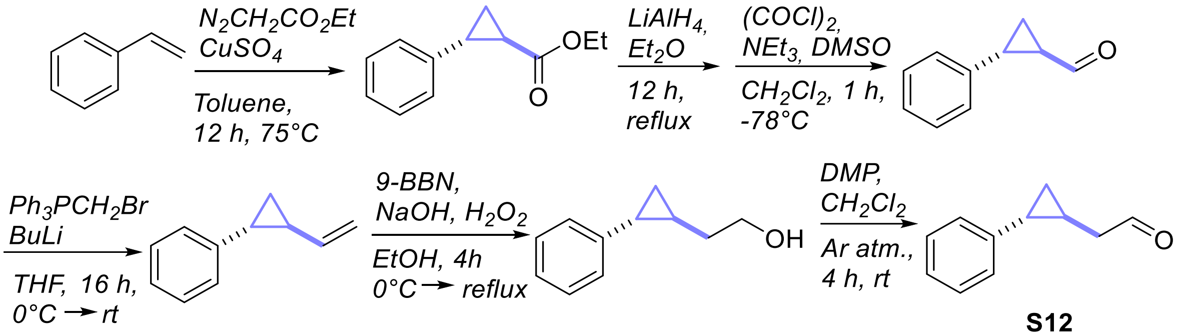

S12

Following a reported procedure,[13] to 355 mg of anhydrous $CuSO_4$ (2.2 mmol, 10 mol%) 10 mL of chloroform and 2.5 mL of styrene (21.7 mmol, 1.0 equiv.) were added. Then a solution of 4.6 mL of ethyl diazoacetate (43.4 mmol, 2.0 equiv.) in 20 mL of chloroform was added dropwise over 1.5 h. After stirring for 14 h at 75°C, using an oil bath, the mixture was concentrated in vacuo to a green oil. This was purified by flash chromatography on silica gel using hexane: ethyl acetate 9:1 as eluent giving a mixture of ethyl 2-phenylcyclopropane-1-carboxylate isomers as a clear oil in 51% yield (2.11 g, 11.1 mmol)

In an oven-dried round-bottom flask 842 mg of $LiAlH_4$ (22.2 mmol, 2.0 equiv.) were dissolved in 25 mL of anhydrous diethyl ether under a nitrogen atmosphere. The reaction mixture was cooled to 0°C and a solution of 2.11 g of the ethyl 2-phenylcyclopropane-1-carboxylate (11.1 mmol, 1.0 equiv.) in 25 mL of anhydrous THF was added dropwise. The reaction mixture was stirred for 12h at reflux, using an oil bath, then the slurry was quenched with 1 mL of NaOH (aq) 15 % w/w and 3 mL of water. The ethereal solution was filtered, and the solid residue was triturated with ether. The combined organic solutions were washed with saturated aqueous NaCl solution and dried (MgSO,). The solvent was removed at reduced pressure yielding 92% of the corresponding alcohol (1.51 g, 10.2 mmol) which was used without further treatment. In an oven-dried round-bottom flask 970 µL of oxalylchloride (11.3 mmol, 1.1 equiv.) were dissolved in 30 mL of anhydrous dichloromethane under a nitrogen atmosphere. The reaction mixture was cooled to -78°C, then 1.8 mL of anhydrous DMSO (24.5 mmol, 2.4 equiv.) and a solution of 1.51 g of the previously synthesized (2-phenylcyclopropyl)methanol (10.2 mmol, 1.0 equiv.) in 15 mL of anhydrous dichloromethane was added dropwise. The reaction mixture was stirred for 15 min, then 7.9 mL of anhydrous triethylamine (51 mmol, 5.0 equiv.) were added and the reaction was stirred for an additional 2h at room temperature. After reaction completion monitored by TLC, the slurry was quenched with the addition of 15 mL of water and extracted with diethylether (2 x 20 mL), dried over $MgSO_4$, filtered, and concentrated under reduced pressure. 2-phenyl-cyclopropane-1-carbaldehyde was obtained in 82% yield (1.23

g, 8.4 mmol) as pale-yellow oil after column chromatography on silica gel (hexane:ethyl acetate 9:1).

In a 250-mL oven-dried round-bottomed flask 3.26 g of methyltriphenylphosphonium bromide (9.1 mmol, 1.1 equiv.) were suspended in 50 mL of anhydrous THF under a nitrogen atmosphere. The mixture was cooled to 0 °C and 3.7 mL of 2.5 M solution of n-butyllithium in hexanes (9.1 mmol, 1.1 equiv.) was added in a dropwise fashion. The cooling bath was removed, and the mixture was stirred at room temperature for 30 min. Next, a solution of 1.22 g of 2- phenylcyclopropane-1-carbaldehyde (8.3 mmol, 1.0 equiv.) in 15 mL of anhydrous THF was added through the septum at room temperature. The mixture was left stirring at room temperature overnight, then it was quenched adding 30 mL of saturated NH_4Cl (aq). The aqueous layer was washed with diethylether (3 x 25 mL). The combined organic layers were dried over $MgSO_4$, filtered, and concentrated under reduced pressure, finally the crude alkene was purified using column chromatography on silica gel (hexane:ethyl acetate 95:5). Pure 2-vinylcyclopropylbenzene was obtained in 64% yield (771 mg, 5.3 mmol) as a pale-yellow oil.

To 765 mg of 2-vinylcyclopropylbenzene (5.3 mmol, 1 equiv.) dissolved in 25 mL of anhydrous THF, a solution of 17 mL of 0.5 M solution of 9-BBN in hexanes (8.5 mmol, 1.6 equiv.) in 48 mL of THF was added dropwise at 0°C. After stirring for 2 h at 0°C and for 1 h at room temperature, excess of the 9-BBN was quenched with 6 mL of ethanol. Then the reaction mixture was oxidized with 4 mL of hydrogen peroxide 30% and 4 mL of a 3 M NaOH (aq) solution stirring the mixture for 1 h at reflux, using an oil bath. After saturating with K_2CO_3, the organic layer was separated and the aqueous phase was extracted with diethylether (3 x 30 mL). The combined diethyl ether and THF solutions were dried over $MgSO_4$, filtered, and concentrated under reduced pressure, pure 2-(2-phenylcyclopropyl)ethan-1-ol was obtained in 58% yield (503 mg, 3.1 mmol) as a colourless oil after column chromatography on silica gel (hexane:ethyl acetate 8:2).

Finally, the alcohol was oxidized to the corresponding aldehyde following the procedure given in chapter C.1 giving **S12** in 86% yield (424 mg, 2.6 mmol) as a colourless oil.

ethyl 2-phenylcyclopropane-1-carboxylate

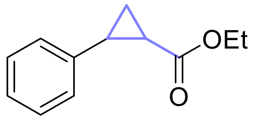

^{1}H NMR (300 MHz, $CDCl_3$): δ 7.35 – 7.00 (m, 5H), 4.17 (q, *J* = 7.1 Hz, 2H), 2.51 (ddd, *J* = 10.2, 6.3, 4.3 Hz, 1H), 1.90 (dt, *J* = 9.3, 4.7 Hz, 1H), 1.59 (dt, *J* = 9.7, 4.9 Hz, 1H), 1.34 – 1.25 (m, 4H) ppm **$^{13}C\{^1H\}$ NMR (75 MHz, $CDCl_3$):** δ 173.4, 140.1, 128.5, 126.5, 126.2, 60.7, 26.2, 24.2, 17.1, 14.3 ppm.

These data matched with the previously reported in literature.[14]

2-phenylcyclopropane-1-carbaldehyde

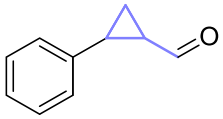

^{1}H NMR (300 MHz, $CDCl_3$): δ 9.33 (d, *J* = 4.5 Hz, 1H), 7.30 (t, *J* = 7.5 Hz, 2H), 7.23 (t, *J* = 7.2 Hz, 1H), 7.12 (d, *J* = 7.7 Hz, 2H), 2.67 – 2.60 (m, 1H), 2.18 (dq, *J* = 8.9, 4.5 Hz, 1H), 1.74 (dt, *J* = 9.6, 5.0 Hz, 1H), 1.53 (dd, *J* = 12.8, 7.5 Hz, 1H) ppm **^{13}C{^{1}H} NMR (75 MHz, $CDCl_3$):** δ 199.8, 139.1, 128.8, 127.0, 126.4, 33.9, 26.7, 16.6 ppm.

These data matched with the previously reported in literature.[15]

(2-vinylcyclopropyl)benzene

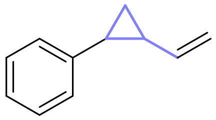

^{1}H NMR (300 MHz, $CDCl_3$): δ 7.30 – 7.23 (m, 2H), 7.16 (t, *J* = 7.4 Hz, 1H), 7.08 (d, *J* = 6.9 Hz, 2H), 5.54 (ddd, *J* = 17.1, 10.3, 8.5 Hz, 1H), 5.11 (ddd, *J* = 17.1, 1.6, 0.7 Hz, 1H), 4.94 (dd, *J* = 10.3, 1.5 Hz, 1H), 1.93 (ddd, *J* = 8.8, 5.7, 4.3 Hz, 1H), 1.71 (ddd, *J* = 8.5, 5.7, 4.2 Hz, 1H), 1.21 (ddd, *J* = 8.5, 5.7, 5.0 Hz, 1H), 1.11 (ddd, *J* = 8.8, 5.3, 5.3 Hz, 1H) ppm **^{13}C{^{1}H} NMR (75 MHz, $CDCl_3$):** δ 142.61, 140.93, 128.61, 125.96, 125.90, 112.81, 27.68, 25.52, 17.02 ppm.

These data matched with the previously reported in literature.[16]

2-(2-phenylcyclopropyl)ethan-1-ol

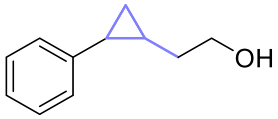

^{1}H NMR (300 MHz, $CDCl_3$): δ 7.30 – 7.25 (m, 2H), 7.19 – 7.15 (m, 1H), 7.10 – 7.06 (m, 2H), 3.80 (t, *J* = 6.5 Hz, 2H), 1.75 – 1.63 (m, 3H), 1.29 (s, 1H), 1.12 (m, 1H), 0.97 (dt, *J* = 8.5, 4.9 Hz, 1H), 0.85 (ddd, *J* = 8.6, 5.6, 4.8 Hz, 1H) ppm **^{13}C{^{1}H} NMR (75 MHz, $CDCl_3$):** δ 143.3, 128.3, 125.6, 125.4, 62.8, 37.3, 22.8, 20.2, 15.6 ppm.

These data matched with the previously reported in literature.[17]

2-(2-phenylcyclopropyl)acetaldehyde (S12)

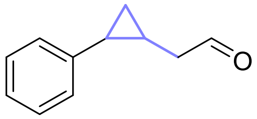

^{1}H NMR (300 MHz, $CDCl_3$): δ 9.88 – 9.82 (m, 2H), 7.35 – 7.23 (m, 2H), 7.23 – 7.17 (m, 1H), 7.16 – 7.07 (m, 2H), 2.69 – 2.31 (m, 1H), 1.84 – 1.74 (m, 1H), 1.43 – 1.30 (m, 1H), 1.15 – 1.04 (m, 1H), 0.96 – 0.84 (m, 1H) ppm **^{13}C{^{1}H} NMR (75 MHz, $CDCl_3$):** δ 201.7, 142.4, 128.5, 126.1, 125.9, 48.3, 22.8, 16.3, 15.4 ppm. **HRMS (ESI-MS)** calculated for $C_{11}H_{13}O^+$ $[M+H]^+$ 161.0961, found 161.0983.

C.7. Preparation of 3-aminobut-2-enenitrile as synthetic precursor

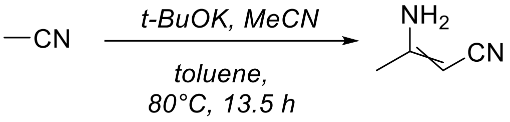

6.970 g (62 mmol, 1.03 equiv,) of *t*-BuOK were added in a 500 mL flame-dried round-bottom flask and stirred under Argon atmosphere at room temperature. After 20 minutes, 130 mL of anhydrous toluene and 3.1 mL (60 mmol, 1.0 equiv.) of anhydrous acetonitrile were added subsequently in anhydrous condition. The resulting mixture was stirred for 1.5 hours refluxing at 80°C, using an oil bath. Other 3.13 mL (60 mmol, 1.0 equiv.) of anhydrous acetonitrile were added to the grainy viscous white mixture which started becoming yellow. The suspension was stirred at 80°C for 12 more hours. The mixture was then added to 100 mL of a water-ice mixture and extracted with ethyl acetate (3 x 100 mL). The collected organic phases were washed once with brine, dried over $MgSO_4$, filtered, and concentrated under reduced pressure giving a clean mixture of *Z* and *E* products in 54% yield (2.67 g, 33 mmol).

3-aminobut-2-enenitrile

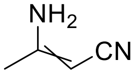

^{1}H NMR (400 MHz, $CDCl_3$): δ 4.69 (2H, s), 3.84 (1H, s), 1.94 (3H, s) ppm.

These data matched with the previously reported in literature.[18]

D. Electrochemical measurements

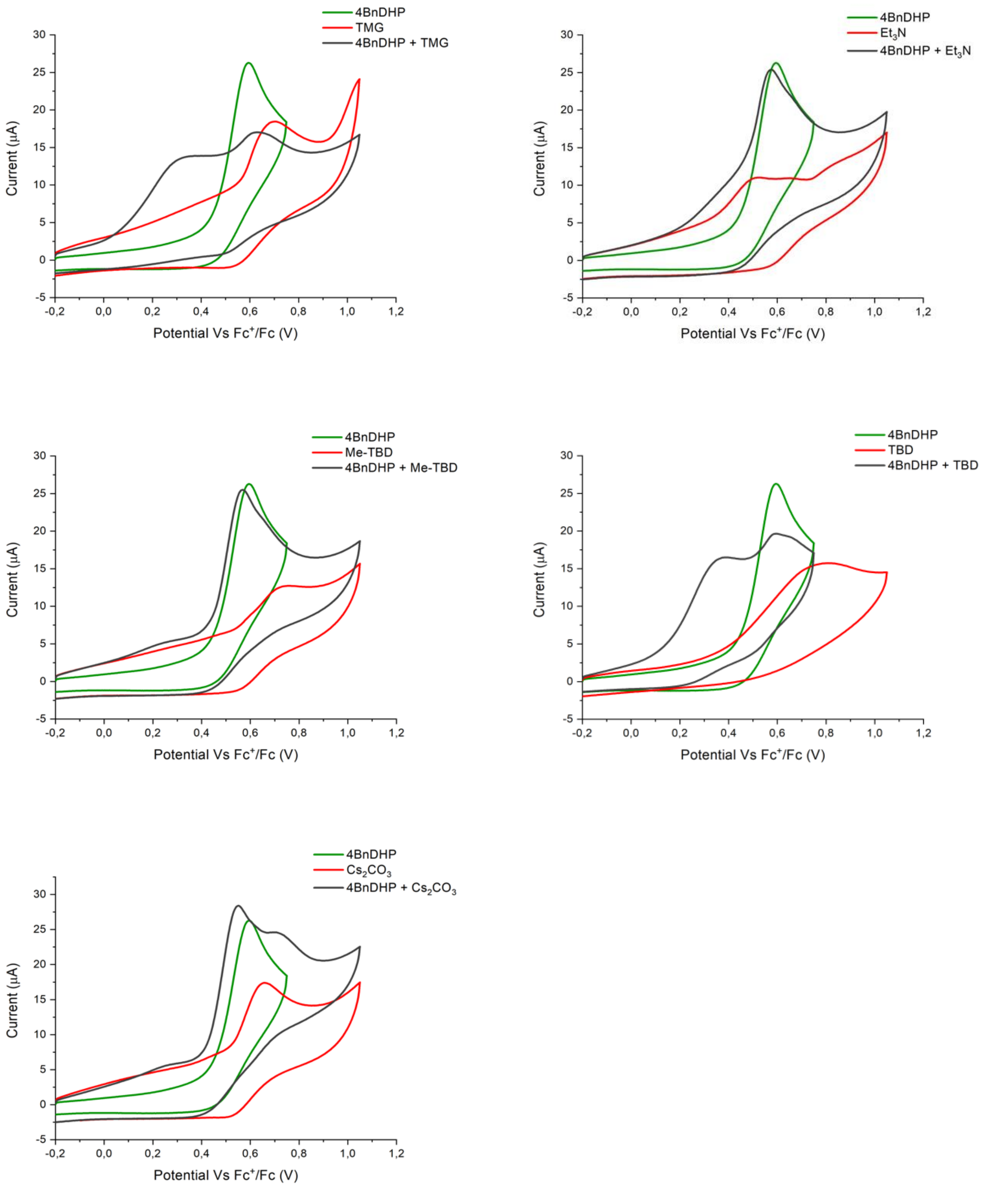

Figure S7. Cyclic voltammetry of **4a** (1mM) with different bases (1.5mM) in DMF/TBAPF$_6$ 0.1 M solution under CO_2 atmosphere, scan rate 100mV/s. TMG: 1,1,3,3-tetramethylguanidine;

Et_3N: triethylamine; Me-TBD: 7-methyl-1,5,7-triazabicyclo[4.4.0]dec-5-ene; TBD: 1,5,7-triazabicyclo[4.4.0]dec-5-ene; Cs_2CO_3: caesium carbonate.

E. SPECTROSCOPIC DATA

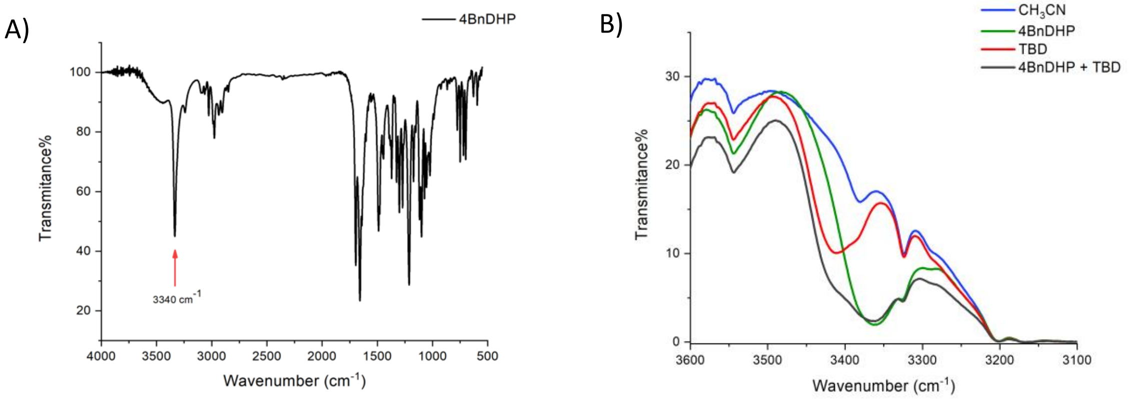

Figure S8. IR spectroscopy of: (A) **4a** on solid state; (B) **4a** 0.05M with TBD 0.075M in MeCN solution. The experiment was carried out through a 1mm quartz cuvette.

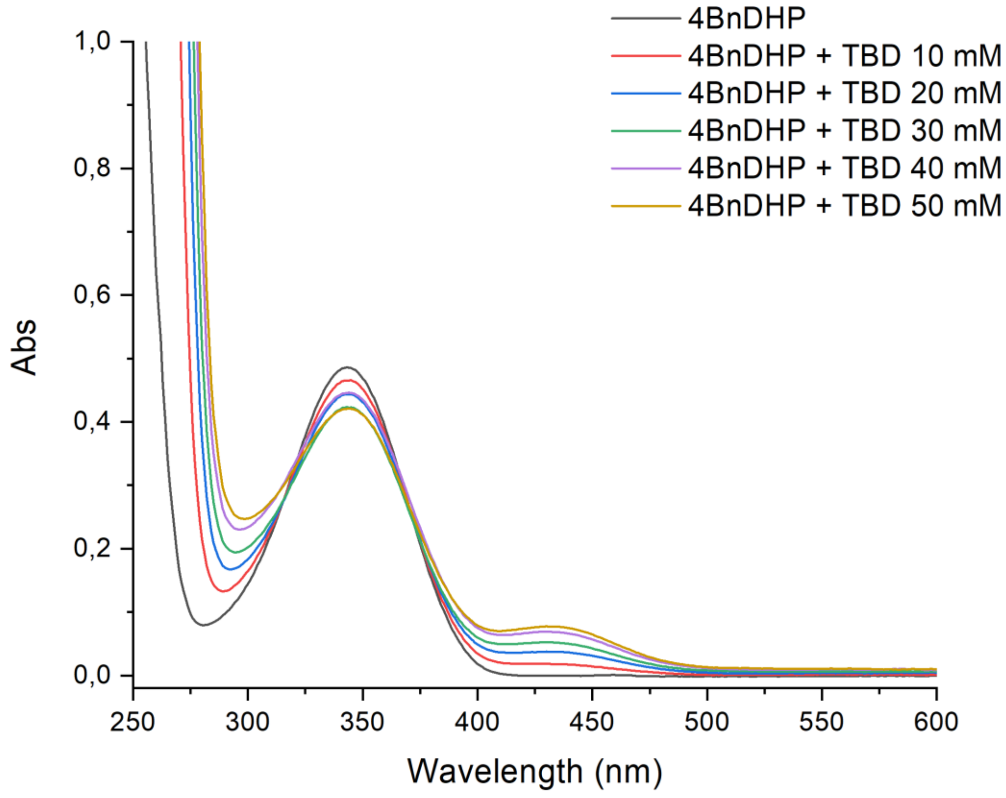

Figure S9. UV-Vis spectra of **4a** 0.1mM with different share of TBD (0.1mM, 1mM, 10mM, 20mM, 30mM, 40mM, 50mM) in MeCN solution (1 cm quartz cuvette).

Table S9. pKa calculation of **4a**. The pKa value of **4a** was estimated from spectroscopic data using the following formulas; in short, the concentration of **4a** upon subsequent TBD additions was estimated by the abatement of the absorption at 343 nm (Abs^{370}, with Abs_0^{370} being the initial absorbance); the concentration of the conjugate base **4a(N⁻)** was then calculated from the difference between the initial concentration of **4a** and the concentration of **4a** after TBD addition. The concentration of $TBDH^+$ was assumed equivalent to the concentration of **4a(N⁻)**. The concentration of TBD was assumed to be equivalent to the , added in large excess with respect to **4a**,

[**4a**]= [**4a**]$_0$ x Abs^{370} / Abs_0^{370}

[**4a(N⁻)**] = [**4a**]$_0$ -[**4a**]

[TBD^+]= [**4a(N⁻)**]

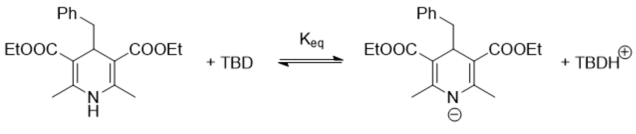

4a **4a(N⁻)**

K_{eq}= {[TBD^+] x [**4a(N⁻)**]}/ {[TBD] x [**4a**]}

$pK_{eq} = \log_{10}(K_{eq})$

pKa(**4a**) = pKa($TBDH^+$/TBD) – pK_{eq} = 26 – pK_{eq}

**[4a(N-H)]$_0$**	**[TBD]**	**[4a]**	**[4a(N⁻)]**	**[$TBDH^+$]**	**K_{eq}**	**pK_{eq}**	**pKa(4a)**
0.1 mM	10 mM	95.9 μM	4.1 μM	4.1 μM	1.6×10^{-5}	-4.8	30.8
0.1 mM	20 mM	91.4 μM	8.6 μM	8.6 μM	3.7×10^{-5}	-4.4	30.4
0.1 mM	30 mM	87.2 μM	12.8 μM	12.8 μM	5.4×10^{-5}	-4.3	30.3
0.1 mM	40 mM	91.9 μM	8.1 μM	8.1 μM	1.6×10^{-5}	-4.8	30.8
0.1 mM	50 mM	86.7 μM	13.3 μM	13.3 μM	3.5×10^{-5}	-4.4	30.4

The pKa(**4a**) was then estimated **30.5±0.3**, from the average of the values reported in table, and with an error bar corresponding to the maximum semidispersion.

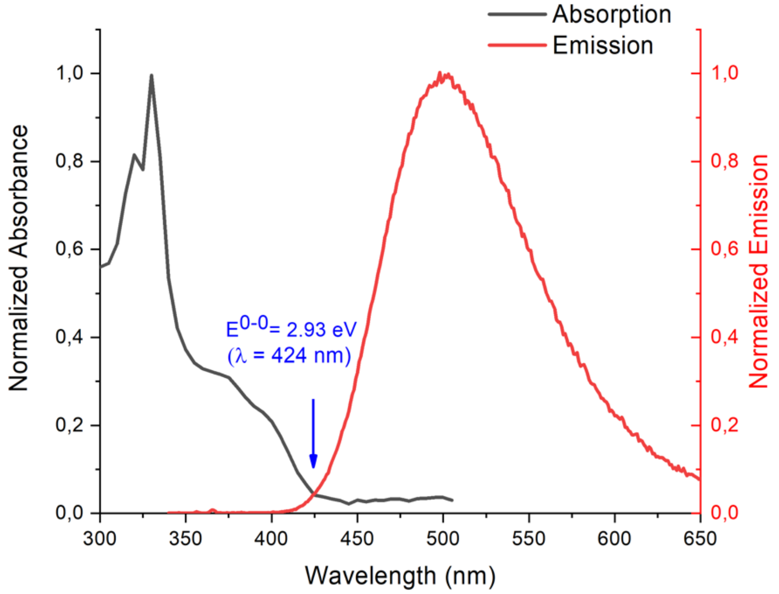

Figure S10. Normalized absorption and emission spectra of **12** in MeCN solution under CO_2 atmosphere.

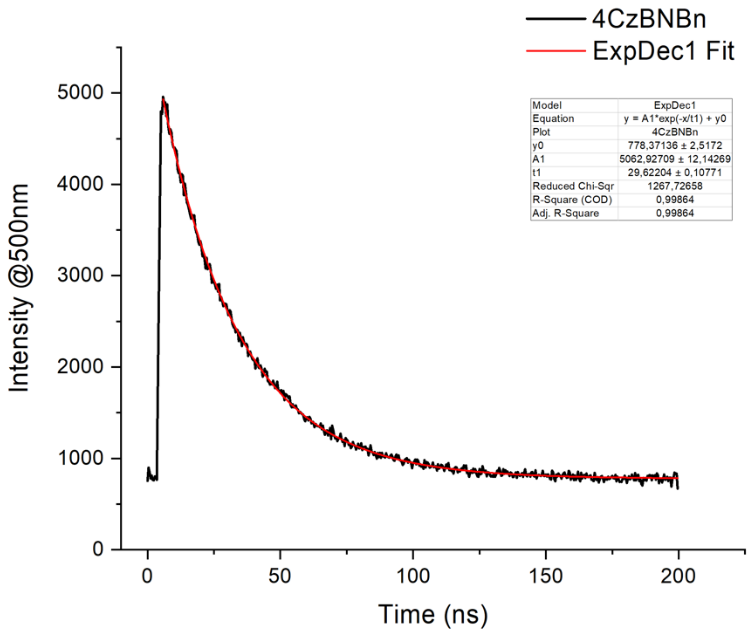

Figure S11. Kinetic analysis at 500 nm of **12** in MeCN solution under CO_2 atmosphere.

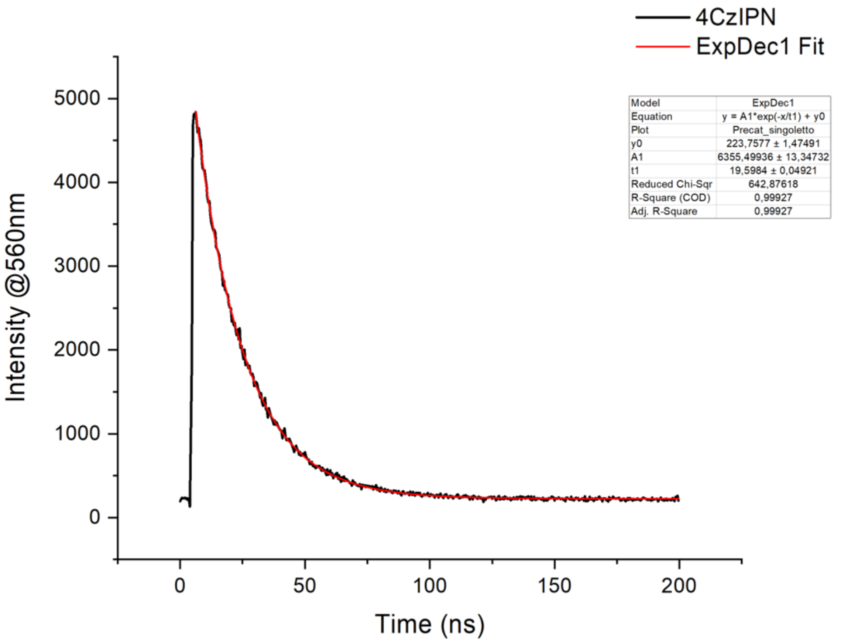

Figure S12. Kinetic analysis at 560 nm of **11** in MeCN solution under N_2 atmosphere.

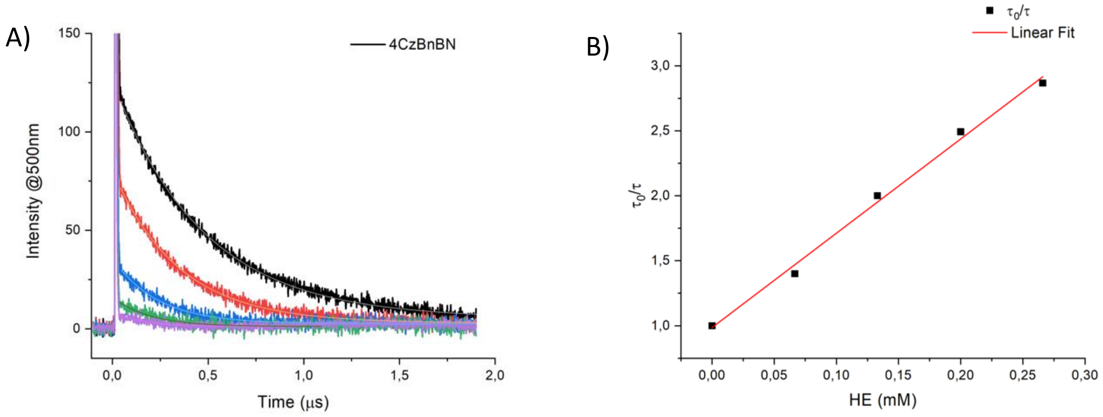

Figure S13. (A) Kinetic analysis of **12** decay at 500 nm in the absence (black trace) and in the presence of 2 μL (red trace), 4 μL (blue trace), 6 μL (green trace), 8 μL (violet trace) of **4a** 0,1M MeCN solution under CO_2 atmosphere. (B) Stern-Volmer plot of quenching of **12** by **4a**.

F. DFT CALCULATIONS

DFT calculations were performed with Gaussian 16,[19] using the density functional theory (DFT) employing the B3LYP method, a 6-311G basis set and (d, p) polarization functions. The self-consistent reaction field (SCRF) was used with DFT energies, optimizations, and frequency calculations to model systems in acetonitrile solution.

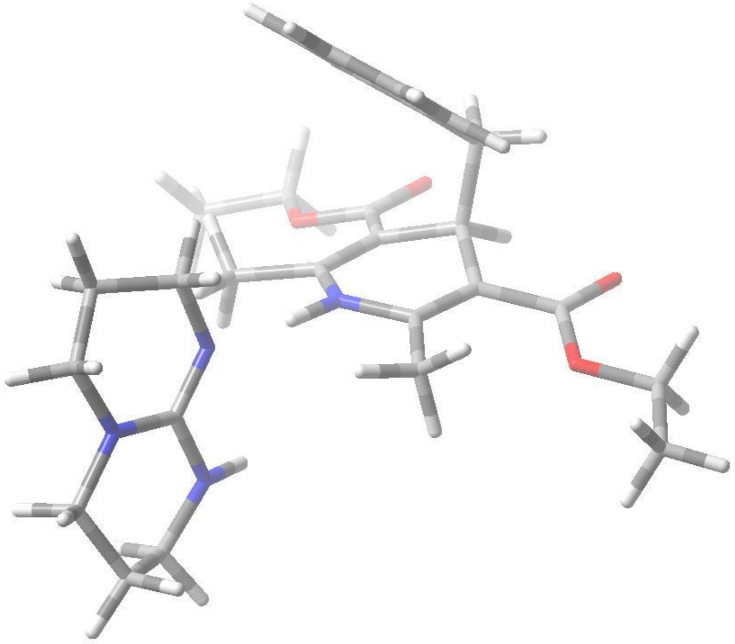

Figure S14. Optimized structure of **4a**-TBD adduct obtained by DFT calculations.

G. ¹H-NMR ANALYSIS

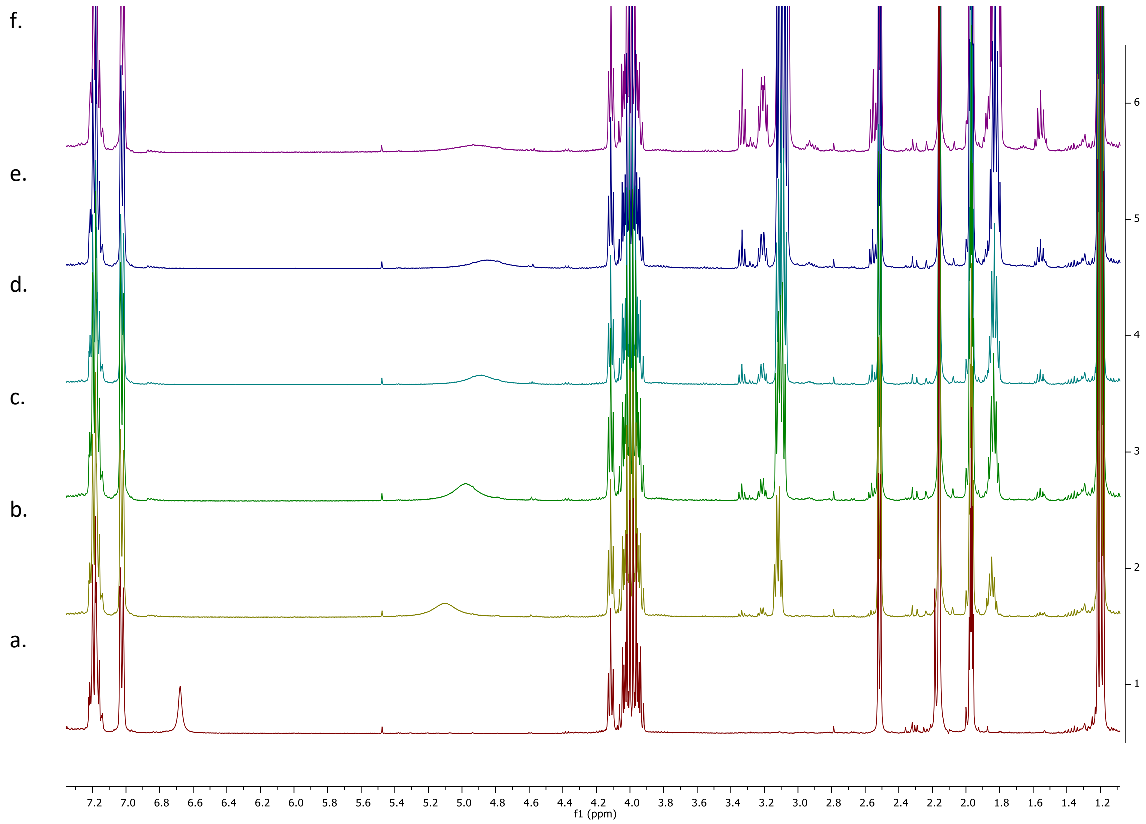

Figure S15. ^{1}H-NMR analysis of **4a** in MeCN-d_3 in the presence of a. 0 equiv., b. 0.1 equiv., c. 0.2 equiv., d. 0.3 equiv., e. 0.5 equiv., f. 1.0 equiv. of TBD.

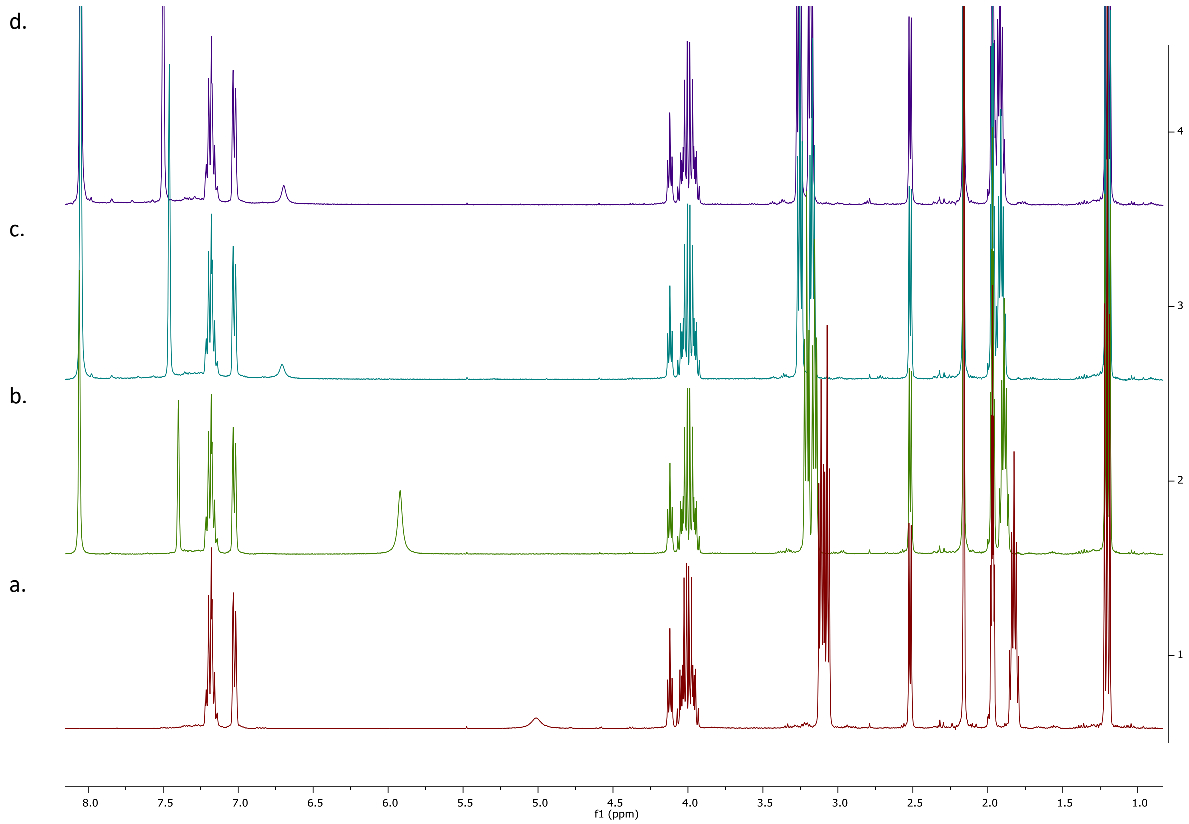

Figure S16. ^{1}H-NMR analysis of **4a** in MeCN-d_3 in the presence of 1.5 equiv. of TBD and a. 0 equiv., b. 0.4 equiv., c. 0.8 equiv., d. 1.0 equiv. of thiourea **9**.

H. REFERENCES

1. a. Donabauer, K.; Maity, M.; Berger, A. L.; Huff, G. S.; Crespi, S.; König, B., Photocatalytic carbanion generation – benzylation of aliphatic aldehydes to secondary alcohols. *Chemical Science* **2019,** *10* (19), 5162-5166; b. Mateos, J.; Rigodanza, F.; Vega-Peñaloza, A.; Sartorel, A.; Natali, M.; Bortolato, T.; Pelosi, G.; Companyó, X.; Bonchio, M.; Dell'Amico, L., Naphthochromenones: Organic Bimodal Photocatalysts Engaging in Both Oxidative and Reductive Quenching Processes. *Angewandte Chemie International Edition* **2020,** *59* (3), 1302-1312; c. McCarthy, B. G.; Pearson, R. M.; Lim, C.-H.; Sartor, S. M.; Damrauer, N. H.; Miyake, G. M., Structure–Property Relationships for Tailoring Phenoxazines as Reducing Photoredox Catalysts. *Journal of the American Chemical Society* **2018,** *140* (15), 5088-5101; d. Speckmeier, E.; Fischer, T. G.; Zeitler, K., A Toolbox Approach To Construct Broadly Applicable Metal-Free Catalysts for Photoredox Chemistry: Deliberate Tuning of Redox Potentials and Importance of Halogens in Donor–Acceptor Cyanoarenes. *Journal of the American Chemical Society* **2018,** *140* (45), 15353-15365.

2. Choo, J. P. S.; Kammerer, R. A.; Li, X.; Li, Z., High-Level Production of Phenylacetaldehyde using Fusion-Tagged Styrene Oxide Isomerase. *Advanced Synthesis & Catalysis* **2021,** *363* (6), 1714-1721.

3. a. Ramaswamy, G. K.; Somasundaram, A.; Kuppuswamy, B. K.; Velayudham, M., Glass Wool Catalysed Regioselective Isomerization of Styrene Oxides. *Journal of the Chinese Chemical Society* **2013,** *60* (1), 97-102; b. Swamy, P.; Reddy, M. M.; Naresh, M.; Kumar, M. A.; Srujana, K.; Durgaiah, C.; Narender, N., Very Important Publication: Hypoiodite-Catalyzed Regioselective Oxidation of Alkenes: An Expeditious Access to Aldehydes in Aqueous Micellar Media. *Advanced Synthesis & Catalysis* **2015,** *357* (6), 1125-1130.

4. Ruff, B. M.; Bräse, S.; O'Connor, S. E., Biocatalytic production of tetrahydroisoquinolines. *Tetrahedron Letters* **2012,** *53* (9), 1071-1074.

5. a. Farid, U.; Aiello, M. L.; Connon, S. J., Highly Enantioselective Catalytic Kinetic Resolution of α-Branched Aldehydes through Formal Cycloaddition with Homophthalic Anhydrides. *Chemistry – A European Journal* **2019,** *25* (43), 10074-10079; b. Shipilovskikh, S. A.; Rubtsov, A. E.; Malkov, A. V., Oxidative Dehomologation of Aldehydes with Oxygen as a Terminal Oxidant. *Organic Letters* **2017,** *19* (24), 6760-6762.

6. Wang, Z.; Xue, F.; Hayashi, T. Synthesis of Arylacetaldehydes by Iridium-Catalyzed Arylation of Vinylene Carbonate with Arylboronic Acids. *Angew. Chem. Int. Ed.* **2019**, *58* (32), 11054-11057.

7. Havare, N.; Plattner, D. A., Oxidative Cleavage of α-Aryl Aldehydes Using Iodosylbenzene. *Organic Letters* **2012,** *14* (19), 5078-5081.

8. Vyas, D. J.; Larionov, E.; Besnard, C.; Guénée, L.; Mazet, C., Isomerization of Terminal Epoxides by a [Pd–H] Catalyst: A Combined Experimental and Theoretical Mechanistic Study. *Journal of the American Chemical Society* **2013,** *135* (16), 6177-6183.

9. Pan, Z.; Li, W.; Zhu, S.; Liu, F.; Wu, H.-H.; Zhang, J., Palladium/TY-Phos-Catalyzed Asymmetric Intermolecular α-Arylation of Aldehydes with Aryl Bromides. *Angewandte Chemie International Edition* **2021,** *60* (34), 18542-18546.

10. Dudnik, A. S.; Schwier, T.; Gevorgyan, V., Gold-Catalyzed Double Migration-Benzannulation Cascade toward Naphthalenes. *Organic Letters* **2008,** *10* (7), 1465-1468.

11. Jean, M.; Renault, J.; van de Weghe, P., Palladium-catalyzed arylation of vinylic acetates. Phosphine ligand influenced regioselectivity. *Tetrahedron Letters* **2009,** *50* (47), 6546-6548.
12. Friest, J. A.; Maezato, Y.; Broussy, S.; Blum, P.; Berkowitz, D. B., Use of a Robust Dehydrogenase from an Archael Hyperthermophile in Asymmetric Catalysis−Dynamic Reductive Kinetic Resolution Entry into (S)-Profens. *Journal of the American Chemical Society* **2010,** *132* (17), 5930-5931.
13. a. Shiraki, R.; Sumino, A.; Tadano, K.-i.; Ogawa, S., Total Synthesis of Natural PI-091, a New Platelet Aggregation Inhibitor of Microbial Origin. *The Journal of Organic Chemistry* **1996,** *61* (8), 2845-2852; b. Le Tadic-Biadatti, M.-H.; Newcomb, M., Picosecond radical kinetics. Rate constants for ring openings of (2-alkoxy-3-phenylcyclopropyl)methyl radicals. *Journal of the Chemical Society, Perkin Transactions 2* **1996,** (7), 1467-1473.
14. Maaskant, R. V.; Polanco, E. A.; van Lier, R. C. W.; Roelfes, G., Cationic iron porphyrins with sodium dodecyl sulphate for micellar catalysis of cyclopropanation reactions. *Organic & Biomolecular Chemistry* **2020,** *18* (4), 638-641.
15. Iosub, A. V.; Moravčík, Š.; Wallentin, C.-J.; Bergman, J., Nickel-Catalyzed Selective Reduction of Carboxylic Acids to Aldehydes. *Organic Letters* **2019,** *21* (19), 7804-7808.
16. Gockel, S. N.; Buchanan, T. L.; Hull, K. L., Cu-Catalyzed Three-Component Carboamination of Alkenes. *Journal of the American Chemical Society* **2018,** *140* (1), 58-61.
17. del Hoyo, A. M.; Herraiz, A. G.; Suero, M. G., A Stereoconvergent Cyclopropanation Reaction of Styrenes. *Angewandte Chemie International Edition* **2017,** *56* (6), 1610-1613.
18. Li, J.; Chen, W.-C.; Liu, H.; Chen, Z.; Chai, D.; Lee, C.-S.; Yang, C., Double-twist pyridine–carbonitrile derivatives yielding excellent thermally activated delayed fluorescence emitters for high-performance OLEDs. *Journal of Materials Chemistry C* **2020,** *8* (2), 602-606.

19. Gaussian 16, Revision C.01, Frisch, M. J.; Trucks, G. W.; Schlegel, H. B.; Scuseria, G. E.; Robb, M. A.; Cheeseman, J. R.; Scalmani, G.; Barone, V.; Petersson, G. A.; Nakatsuji, H.; Li, X.; Caricato, M.; Marenich, A. V.; Bloino, J.; Janesko, B. G.; Gomperts, R.; Mennucci, B.; Hratchian, H. P.; Ortiz, J. V.; Izmaylov, A. F.; Sonnenberg, J. L.; Williams-Young, D.; Ding, F.; Lipparini, F.; Egidi, F.; Goings, J.; Peng, B.; Petrone, A.; Henderson, T.; Ranasinghe, D.; Zakrzewski, V. G.; Gao, J.; Rega, N.; Zheng, G.; Liang, W.; Hada, M.; Ehara, M.; Toyota, K.; Fukuda, R.; Hasegawa, J.; Ishida, M.; Nakajima, T.; Honda, Y.; Kitao, O.; Nakai, H.; Vreven, T.; Throssell, K.; Montgomery, J. A., Jr.; Peralta, J. E.; Ogliaro, F.; Bearpark, M. J.; Heyd, J. J.; Brothers, E. N.; Kudin, K. N.; Staroverov, V. N.; Keith, T. A.; Kobayashi, R.; Normand, J.; Raghavachari, K.; Rendell, A. P.; Burant, J. C.; Iyengar, S. S.; Tomasi, J.; Cossi, M.; Millam, J. M.; Klene, M.; Adamo, C.; Cammi, R.; Ochterski, J. W.; Martin, R. L.; Morokuma, K.; Farkas, O.; Foresman, J. B.; Fox, D. J. Gaussian, Inc., Wallingford CT, 2016.

I. NMR Spectra

S12 - ^{1}H NMR ($CDCl_3$)

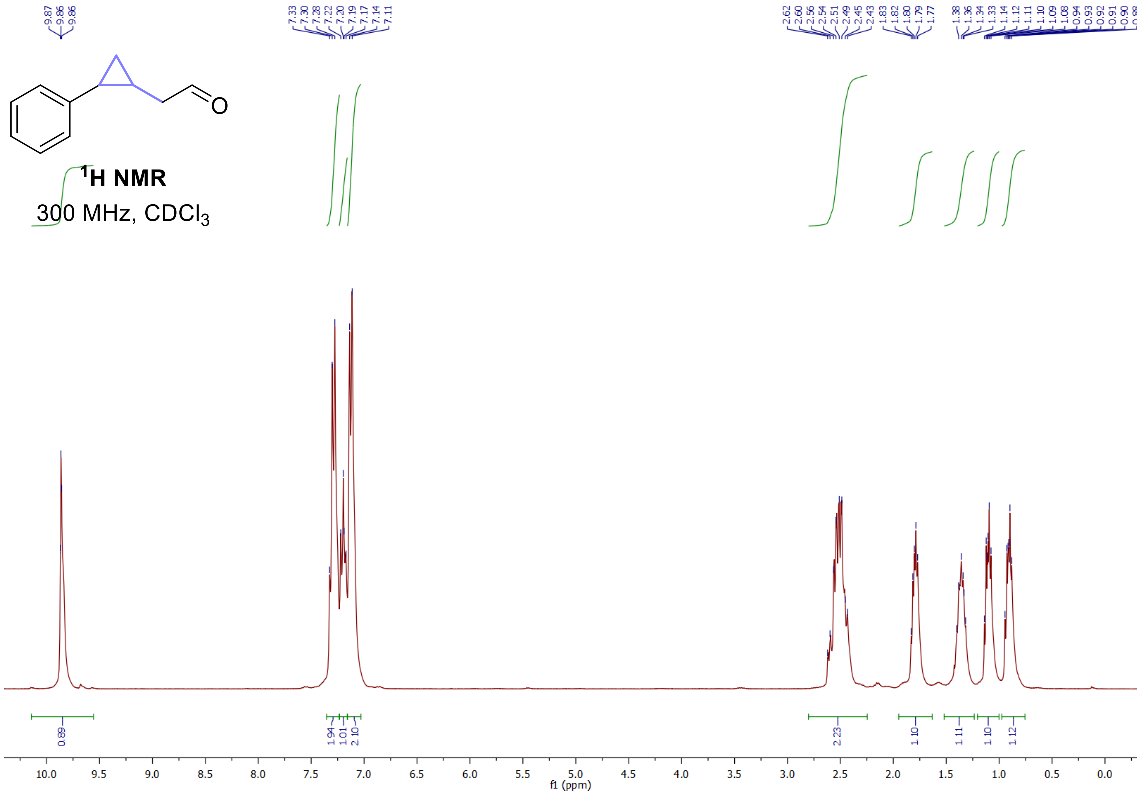

S12- $^{13}C\{^{1}H\}$ NMR ($CDCl_3$)

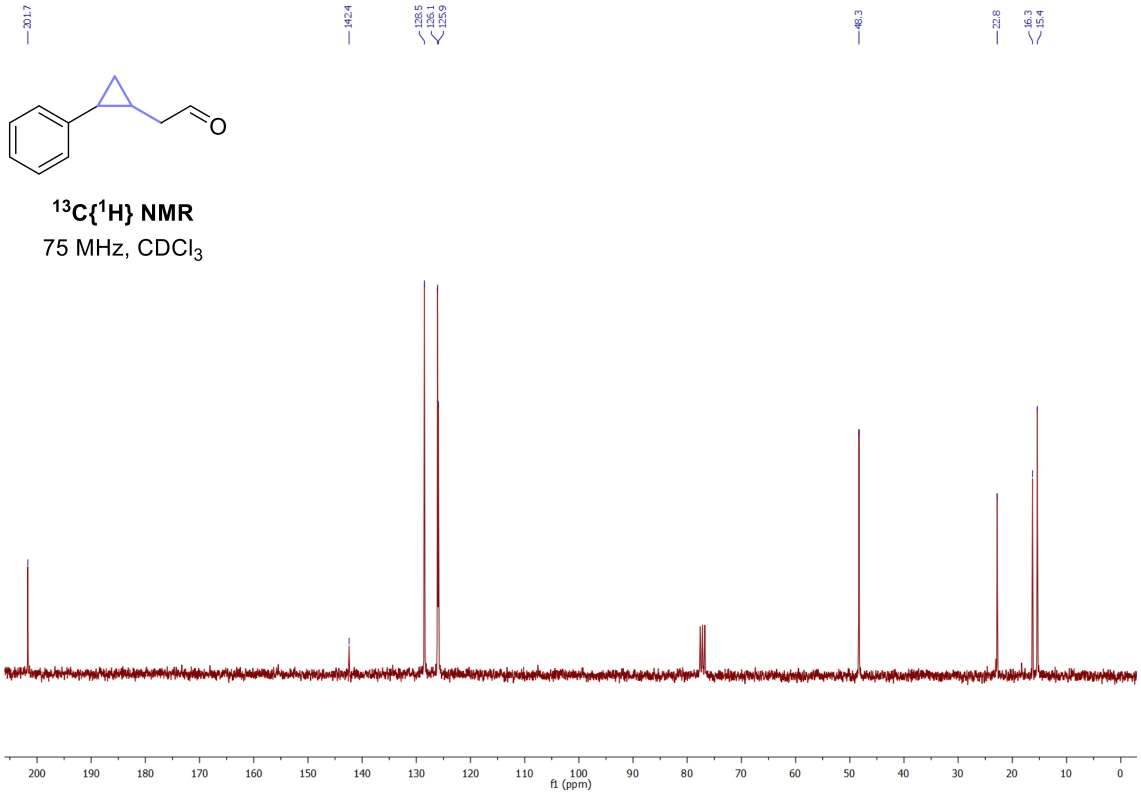

4b - ^{1}H NMR ($CDCl_3$)

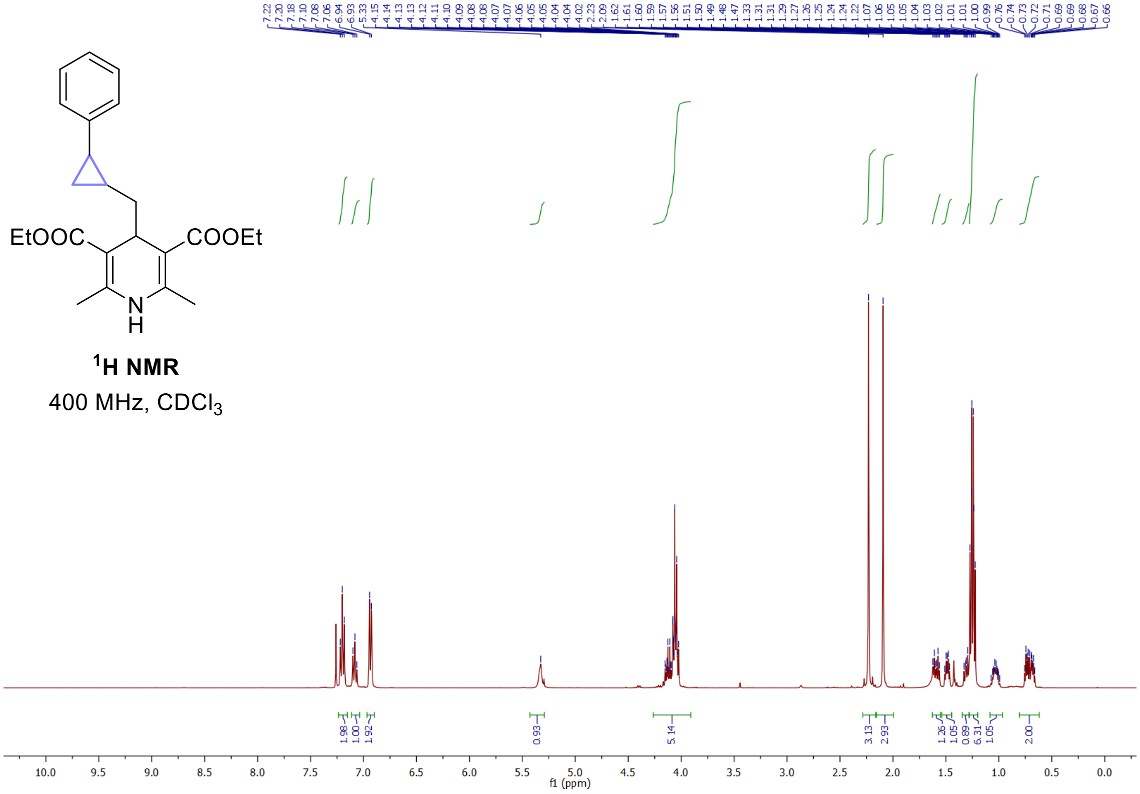

4b - $^{13}C\{^{1}H\}$ NMR ($CDCl_3$)

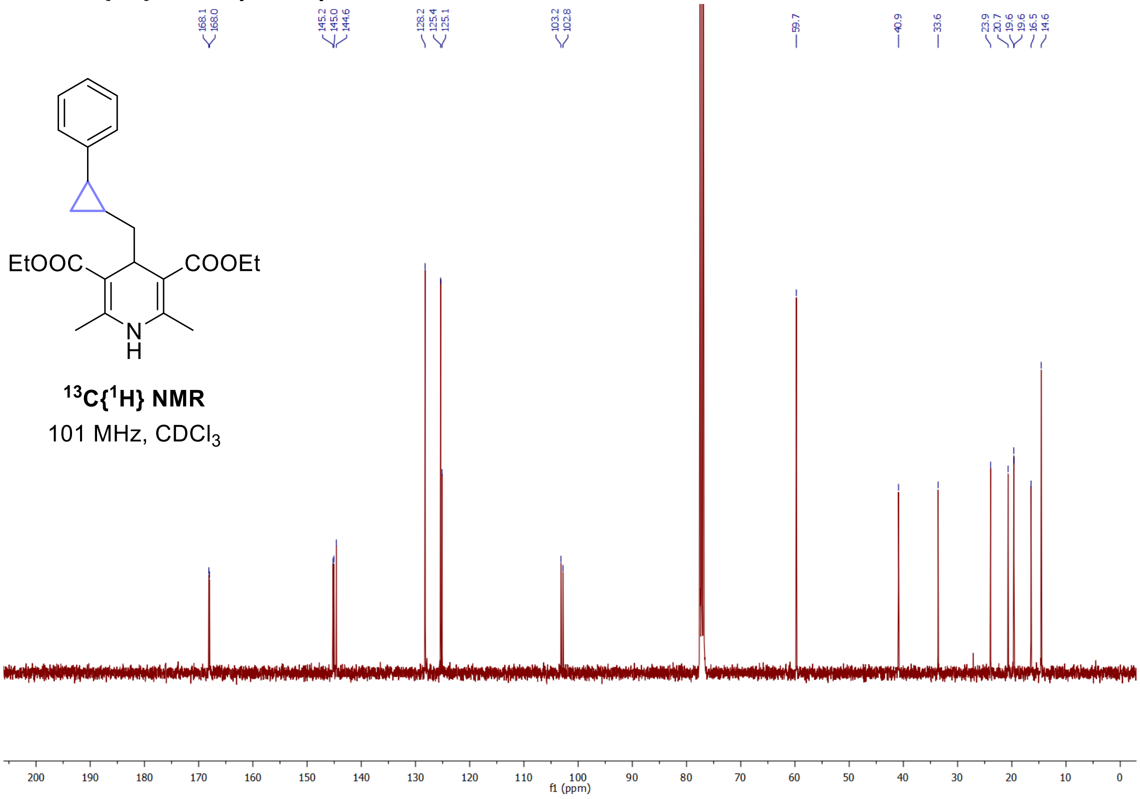

4d - ^{1}H NMR ($CDCl_3$)

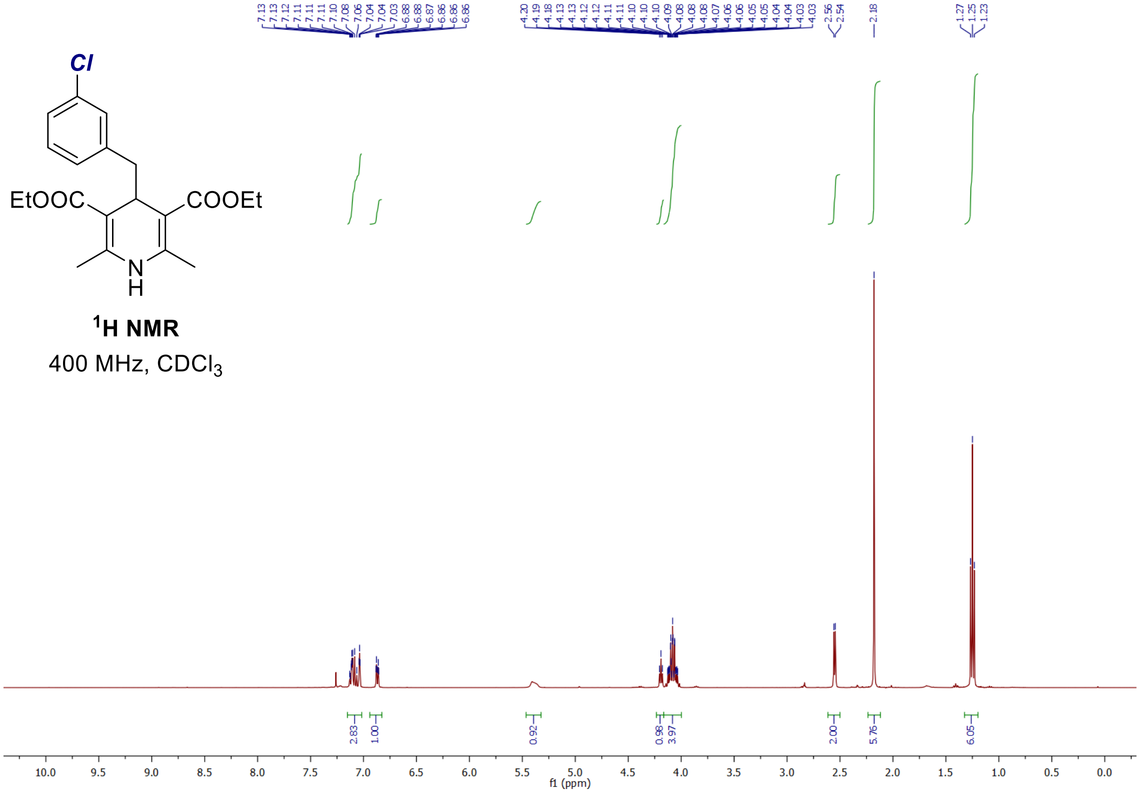

4d - $^{13}C\{^{1}H\}$ NMR ($CDCl_3$)

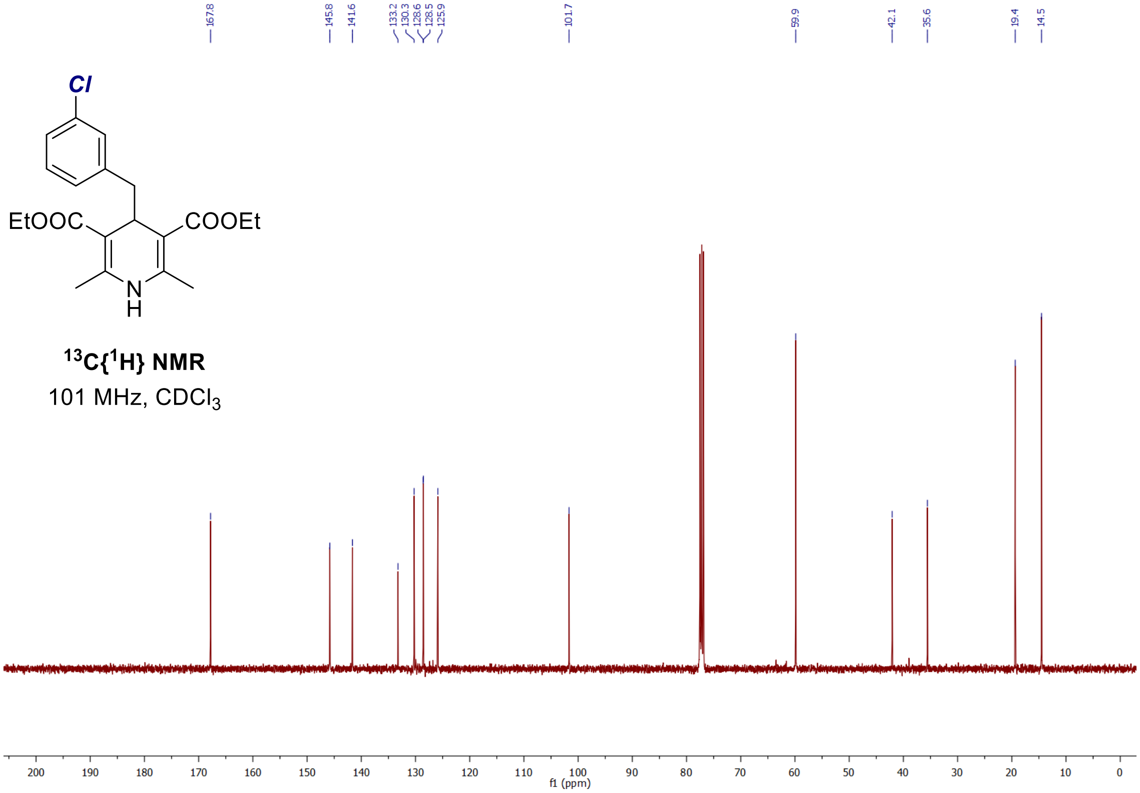

4e - ^1H NMR ($CDCl_3$)

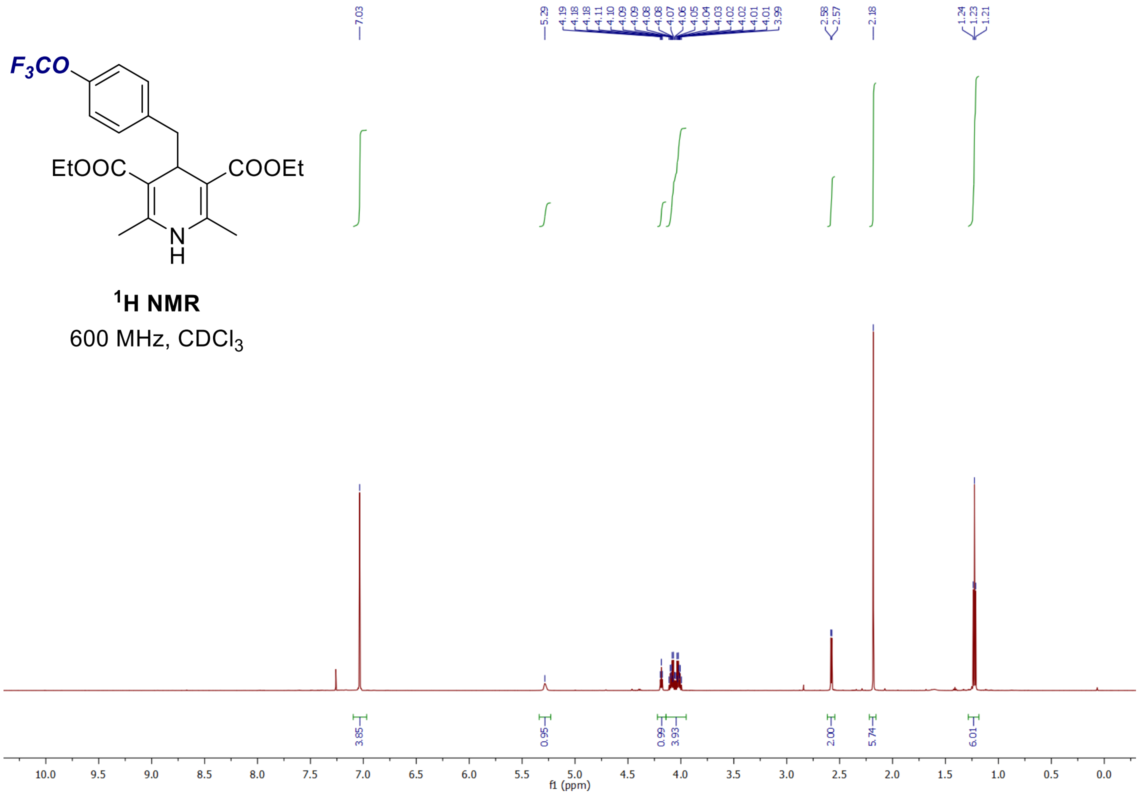

4e - $^{13}C\{^1H\}$ NMR ($CDCl_3$)

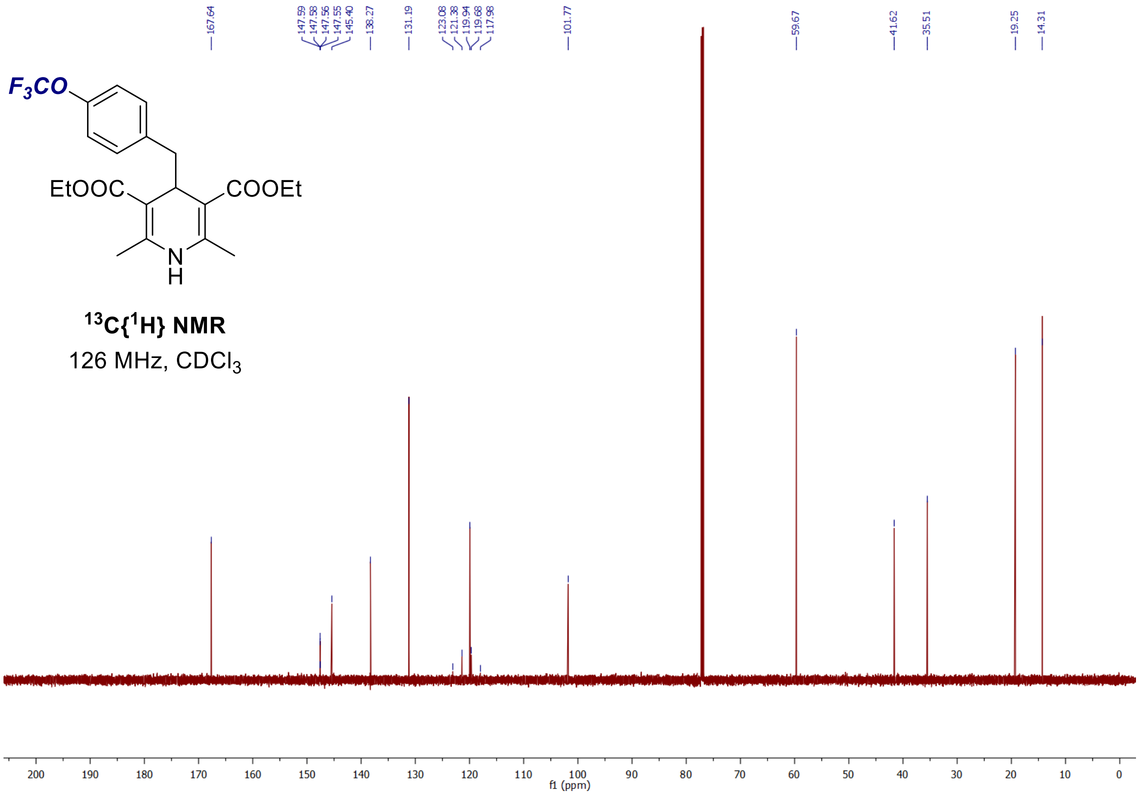

4f - ^{1}H NMR ($CDCl_3$)

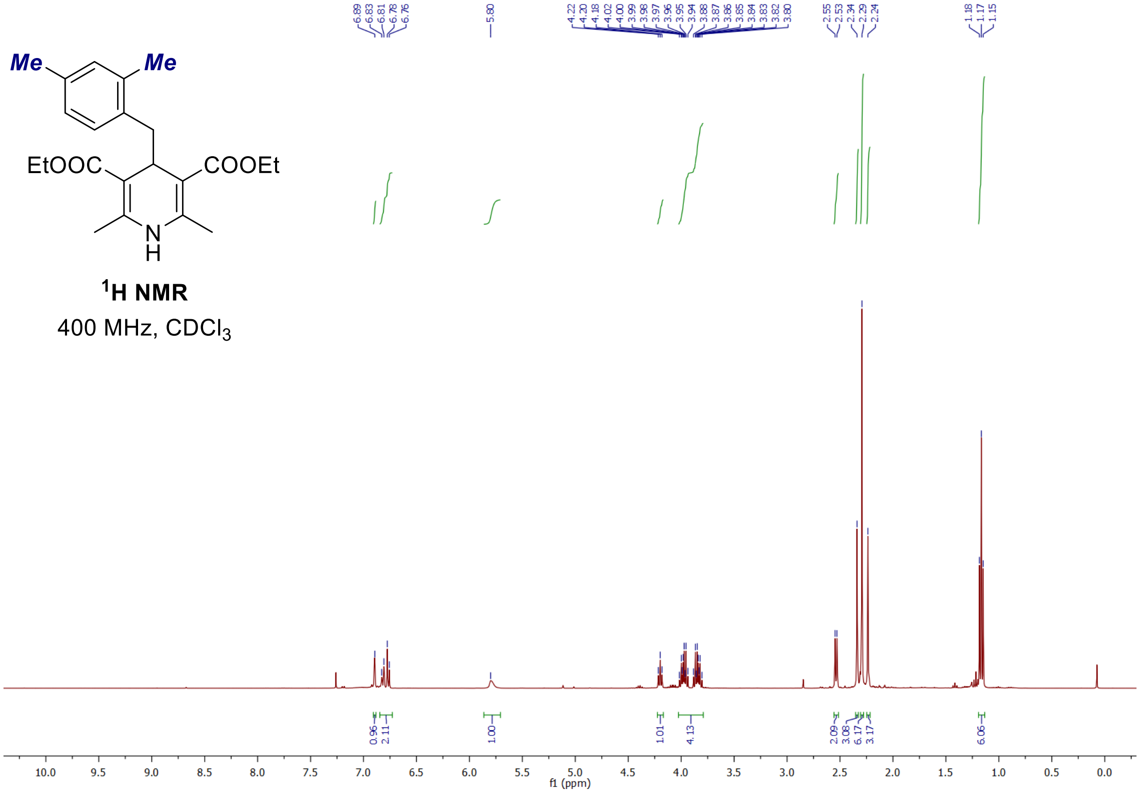

4f - $^{13}C\{^1H\}$ NMR ($CDCl_3$)

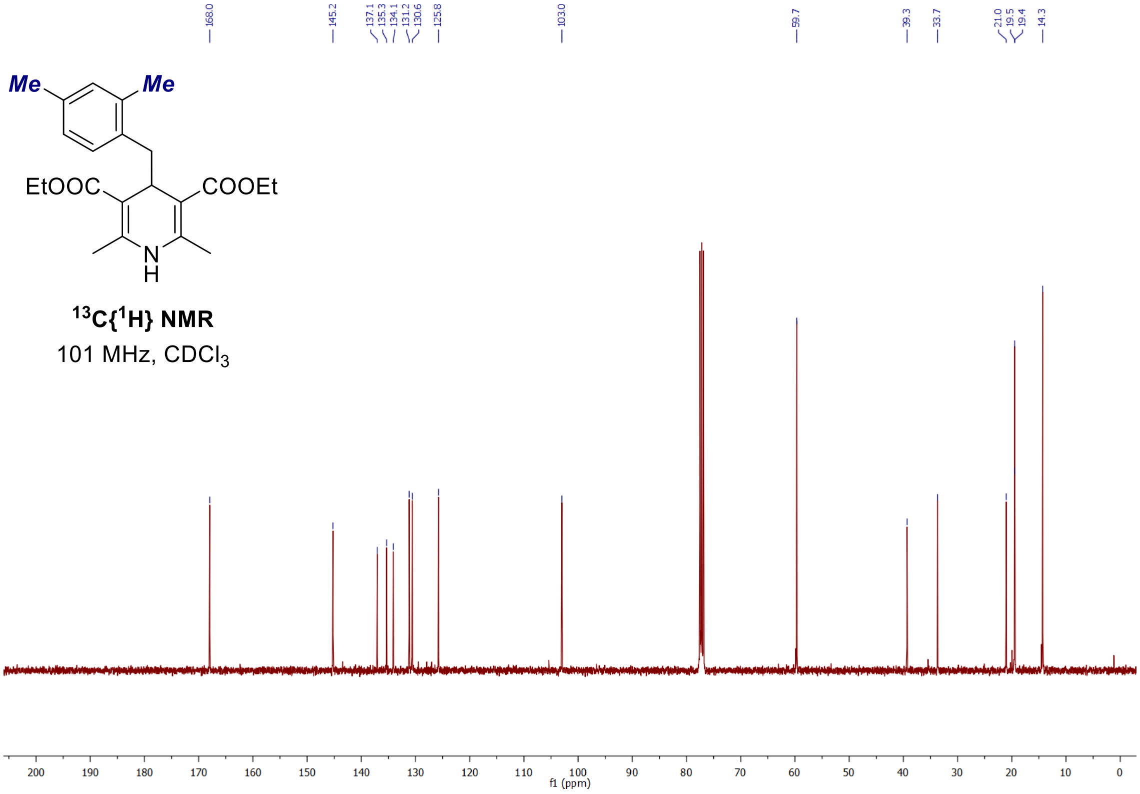

4h - ^{1}H NMR (400MHz) $CDCl_3$

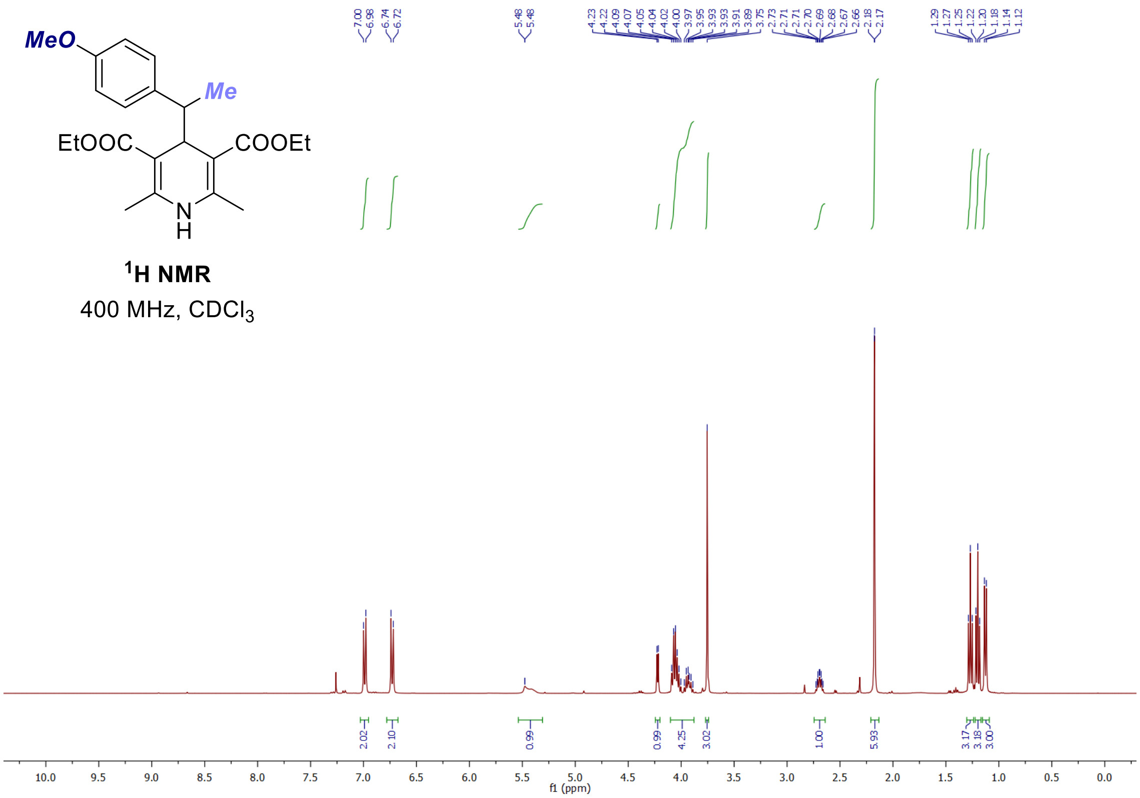

4h - $^{13}C\{^{1}H\}$ NMR (101 MHz) $CDCl_3$

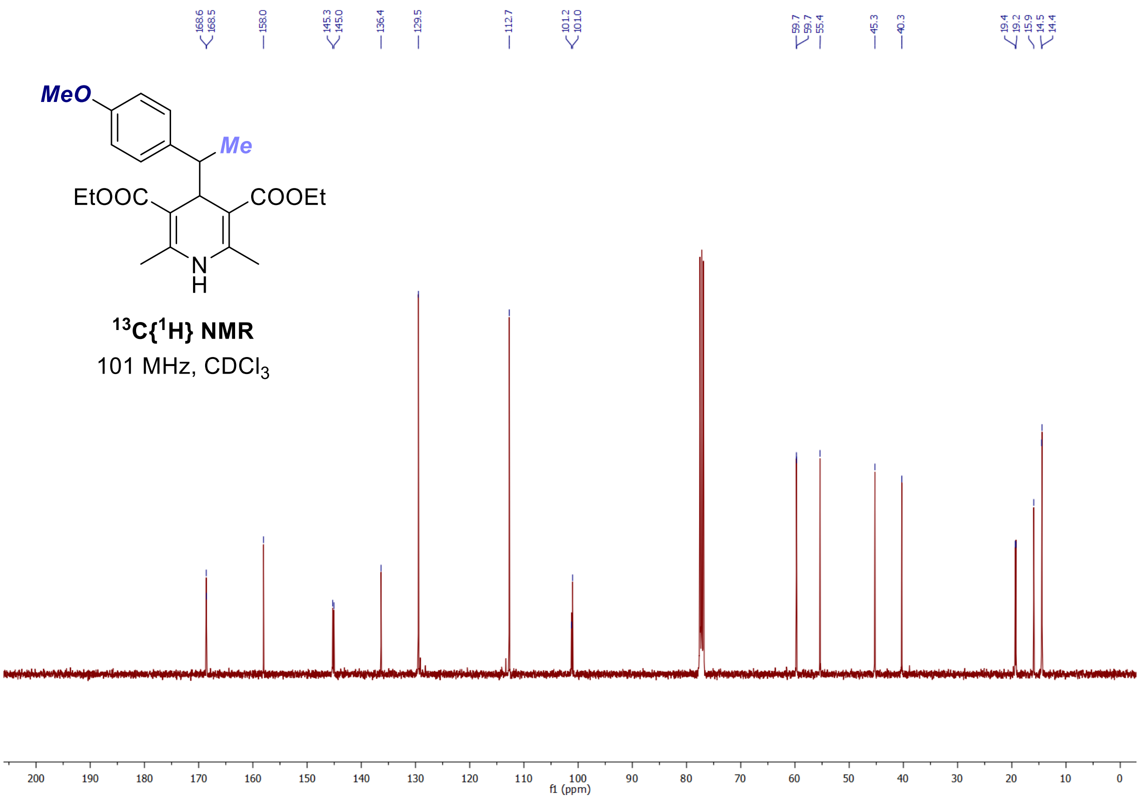

4j - ^{1}H NMR ($CDCl_3$)

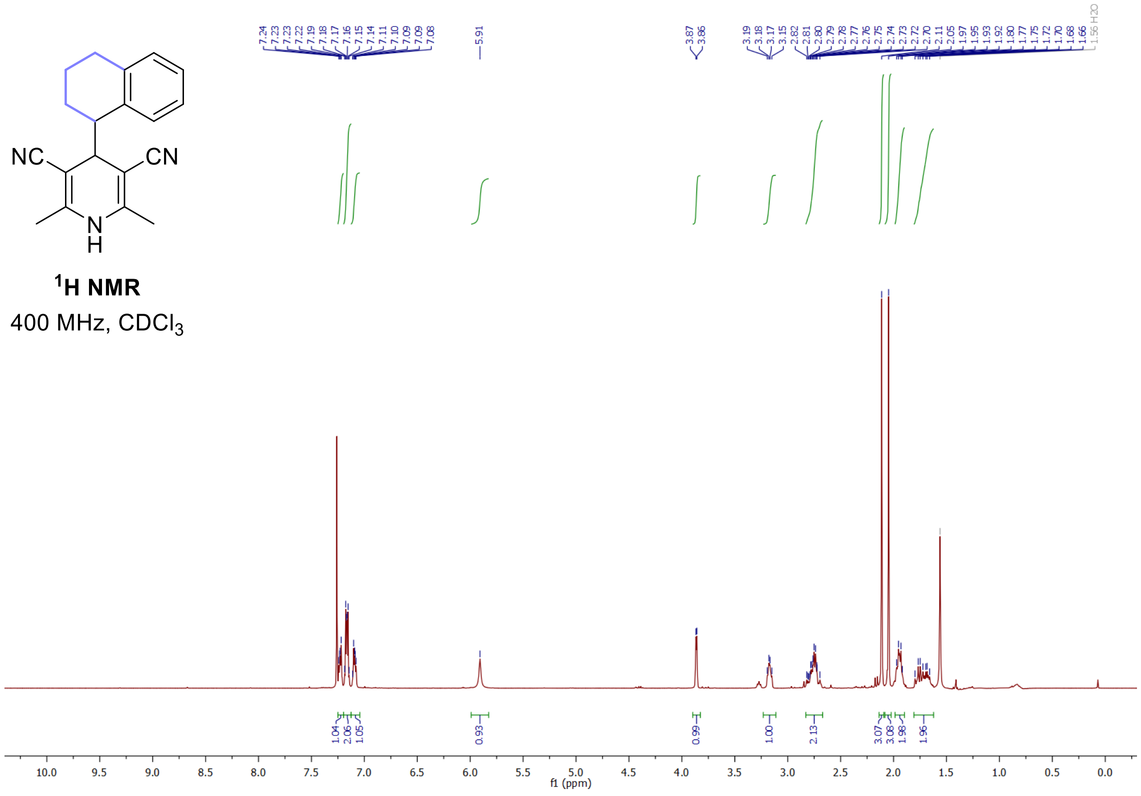

4j - ^{13}C{^{1}H} NMR ($CDCl_3$)

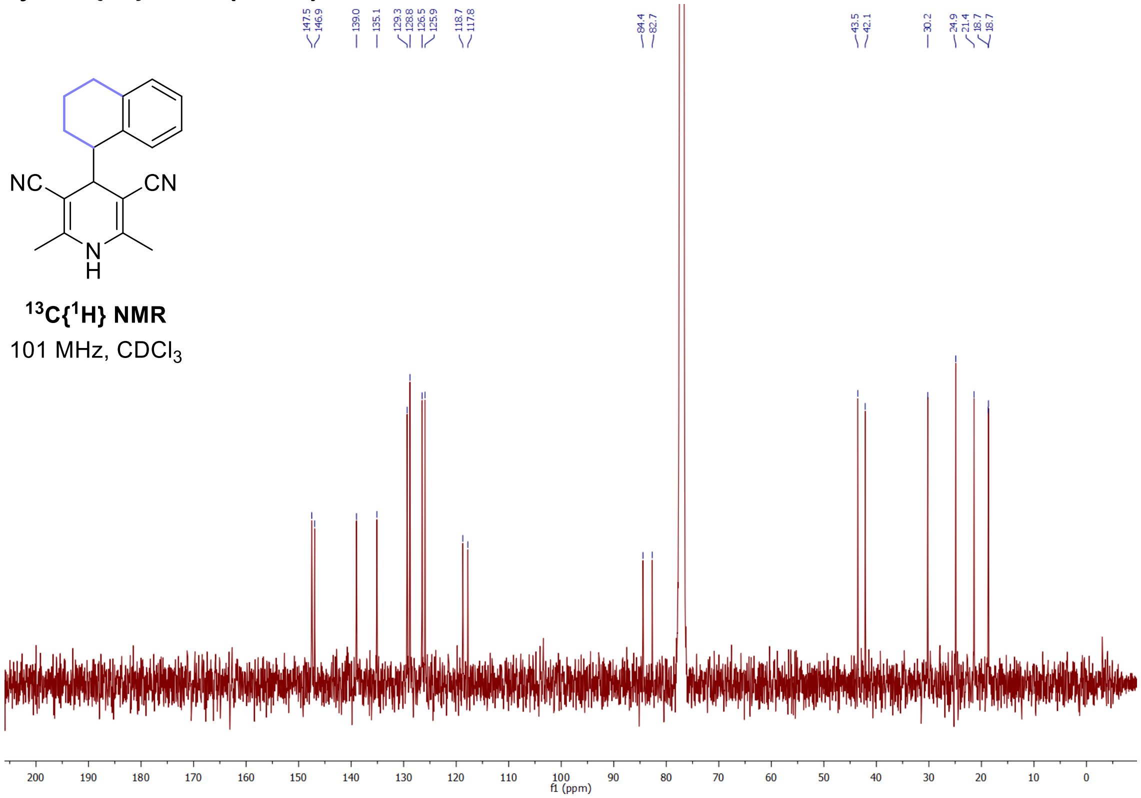

4l - ^{1}H NMR ($CDCl_3$)

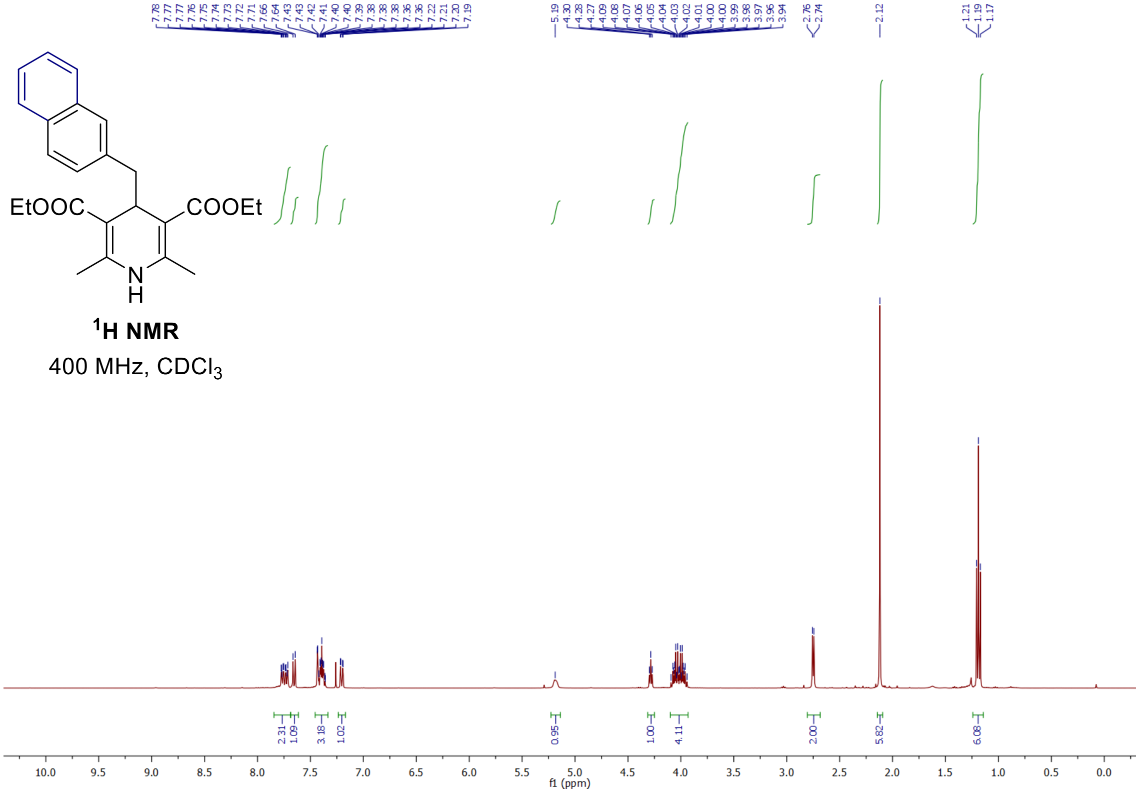

4l - $^{13}C\{^{1}H\}$ NMR ($CDCl_3$)

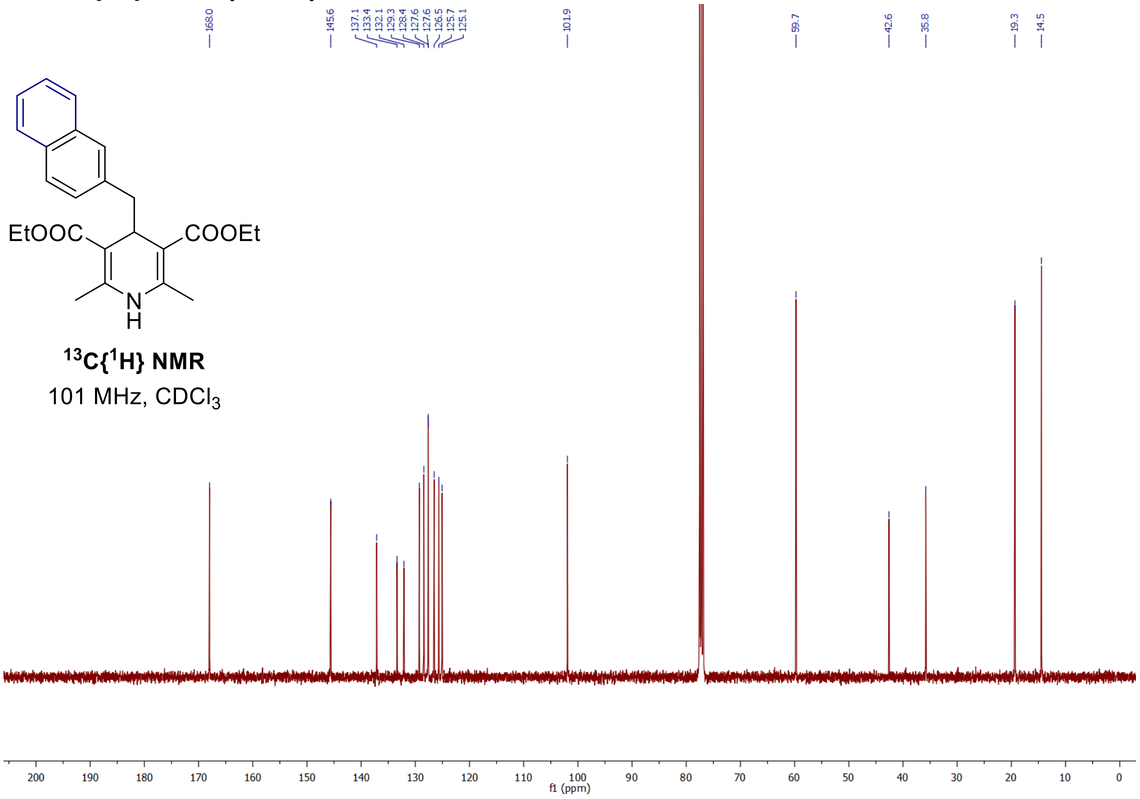

4n - ^{1}H NMR ($CDCl_3$)

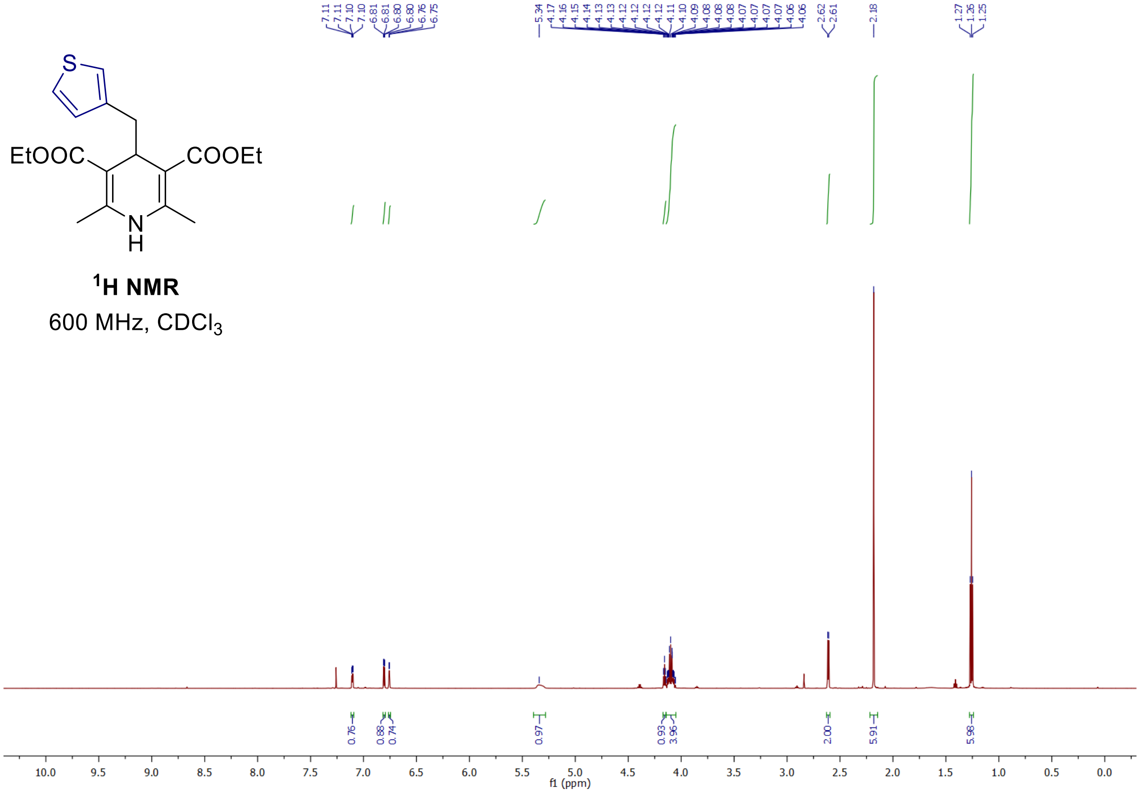

4n - ^{13}C{^{1}H} NMR ($CDCl_3$)

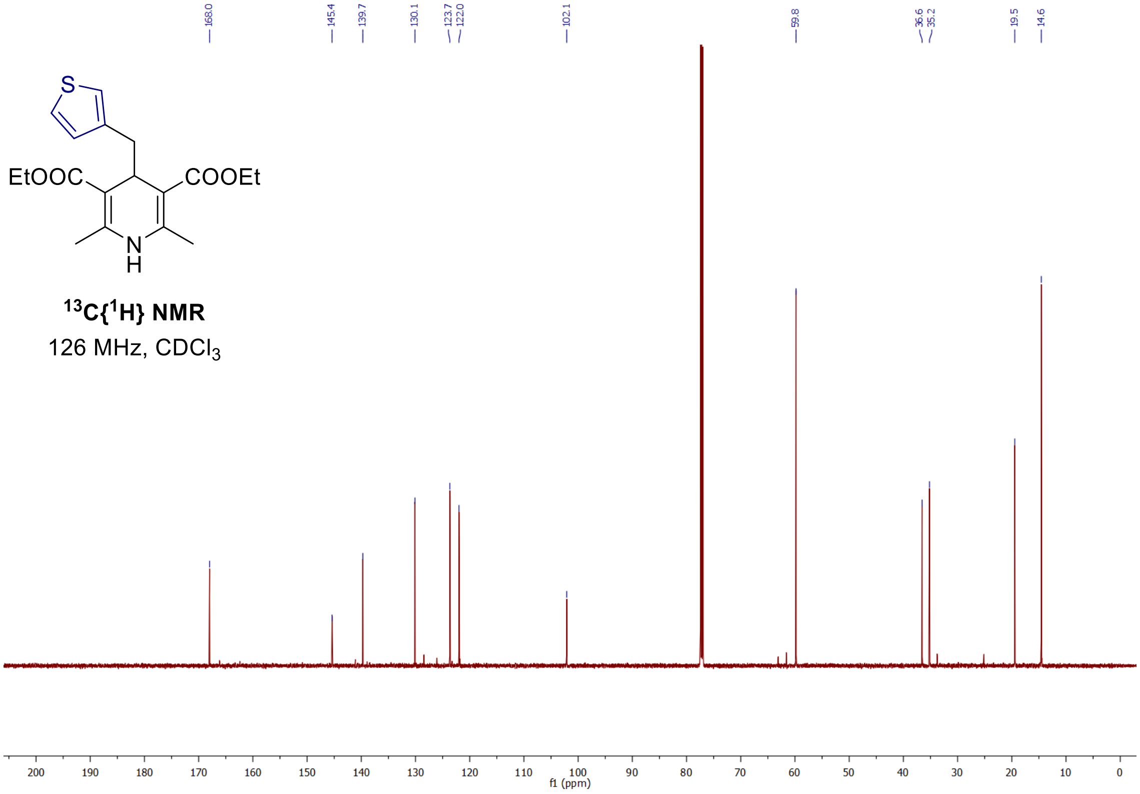

10 - ^{1}H NMR ($CDCl_3$) 0.1 mmol scale reaction

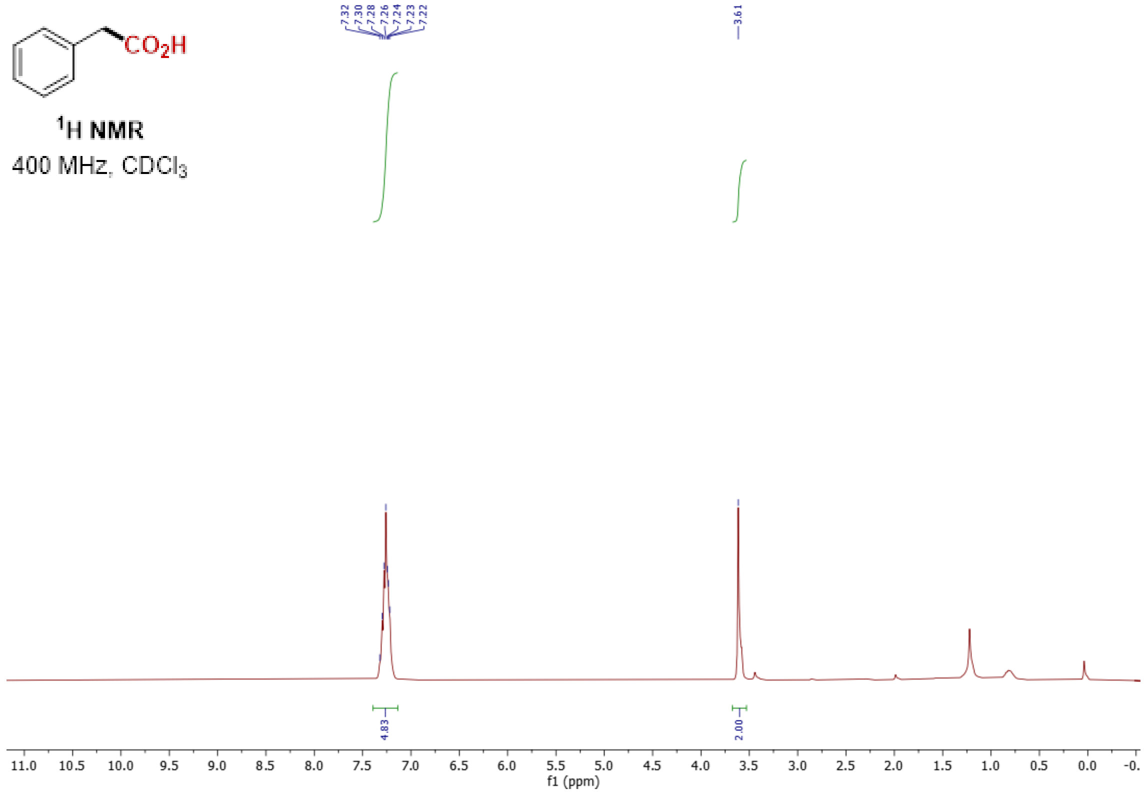

10 - ^{1}H NMR ($CDCl_3$) 1.0 mmol scale reaction

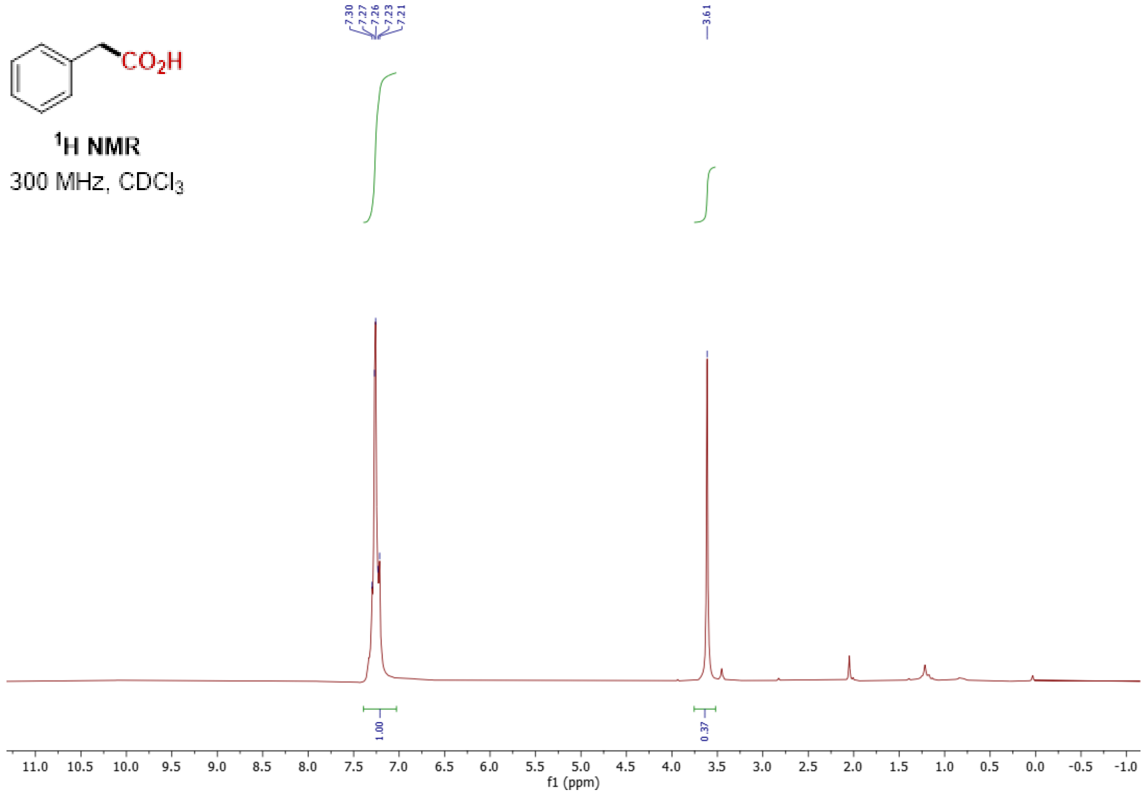

14 - ^{1}H NMR ($CDCl_3$)

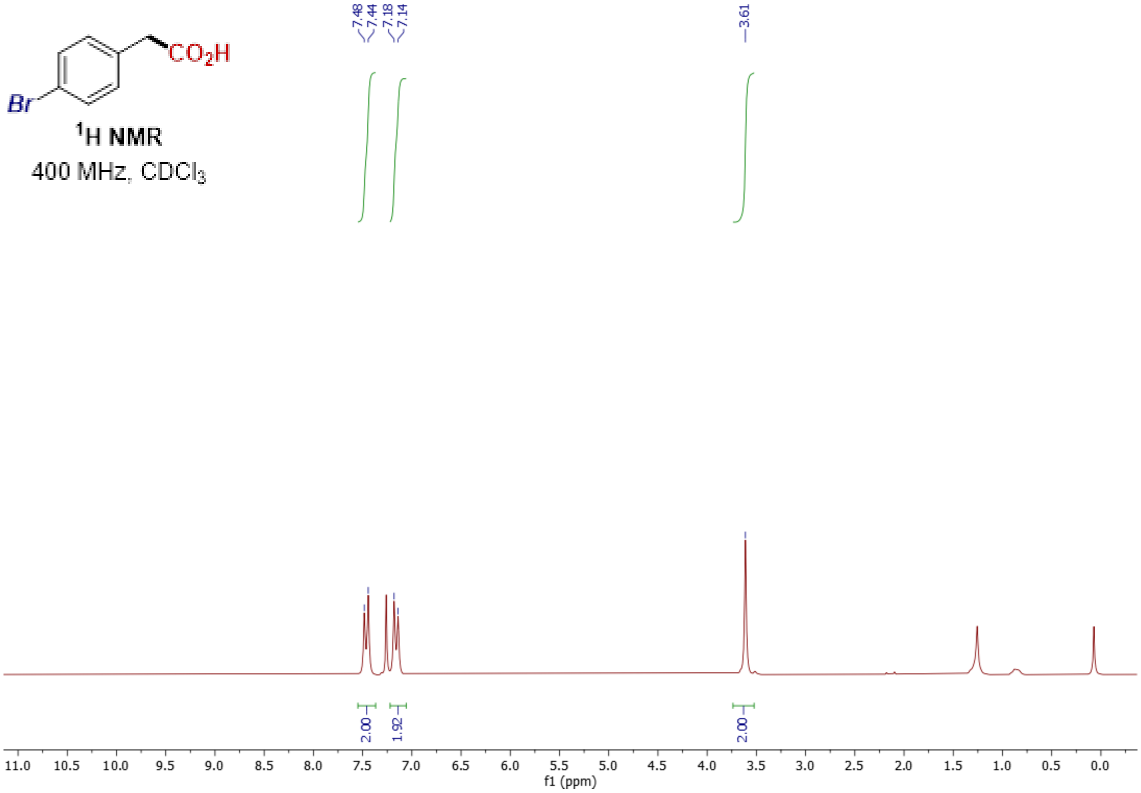

17 - ^{1}H NMR ($CDCl_3$)

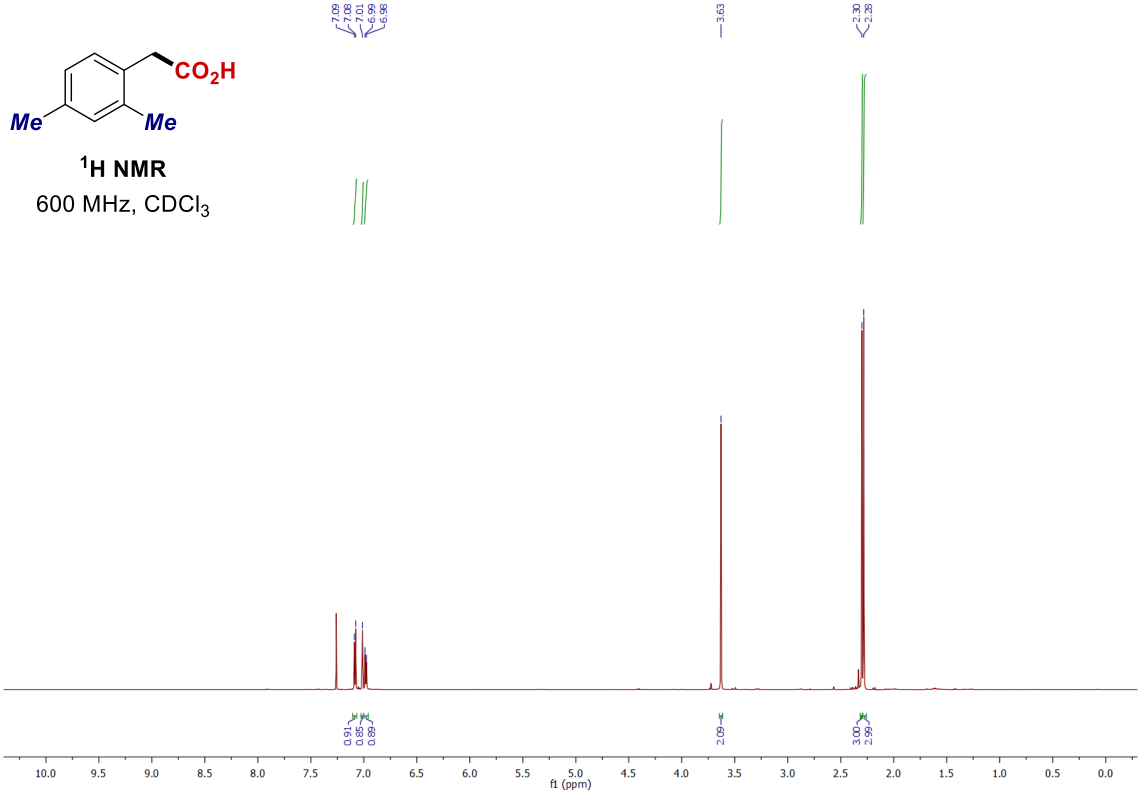

17 - $^{13}C\{^{1}H\}$ NMR ($CDCl_3$)

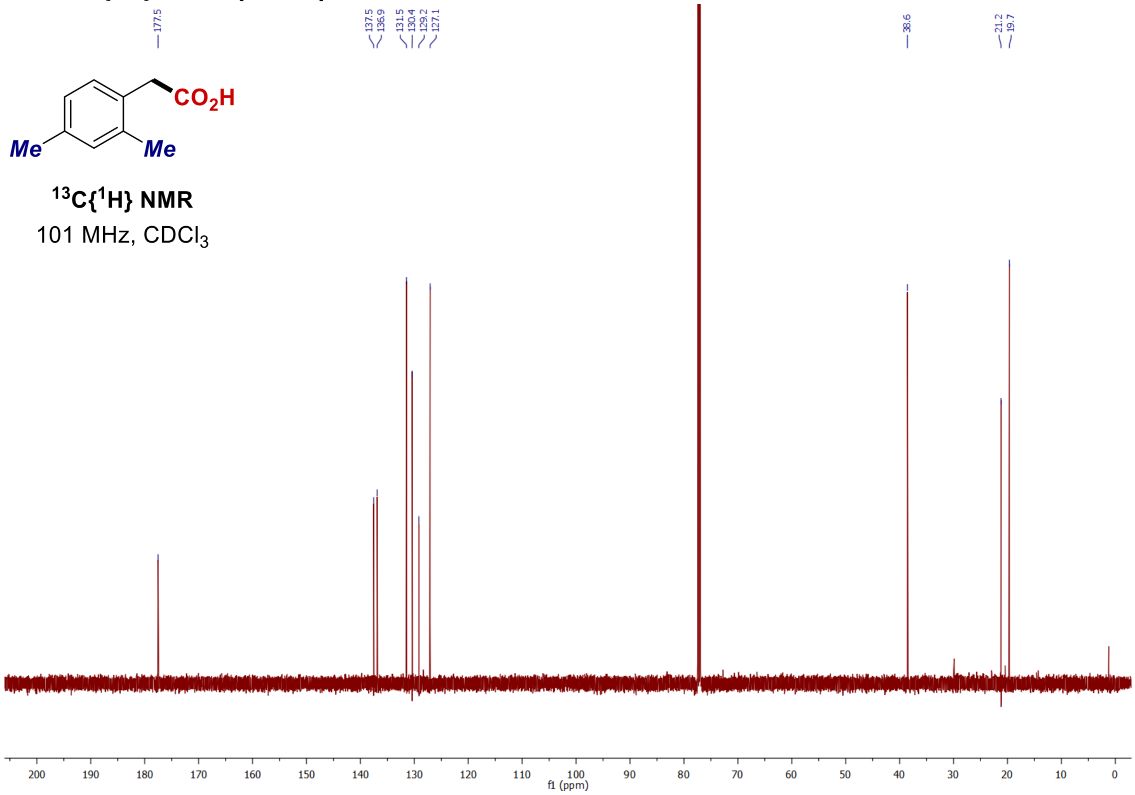

18 - ^{1}H NMR ($CDCl_3$)

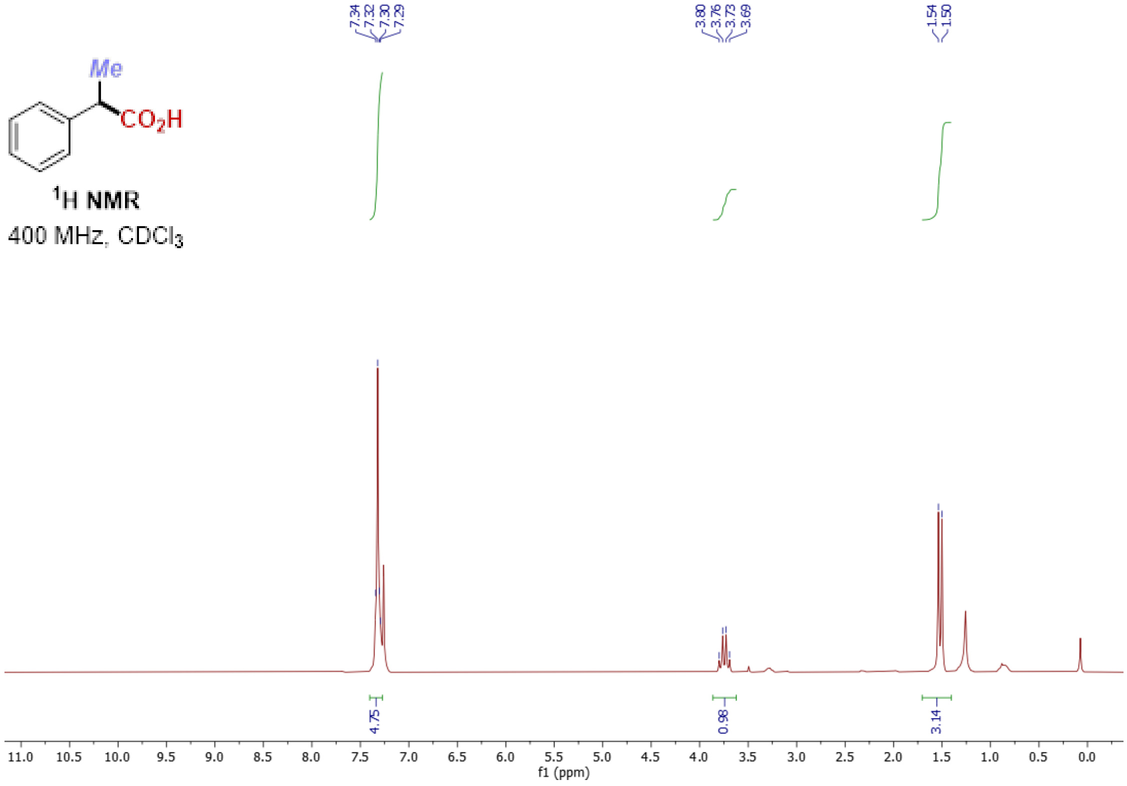

19 - ^{1}H NMR ($CDCl_3$)

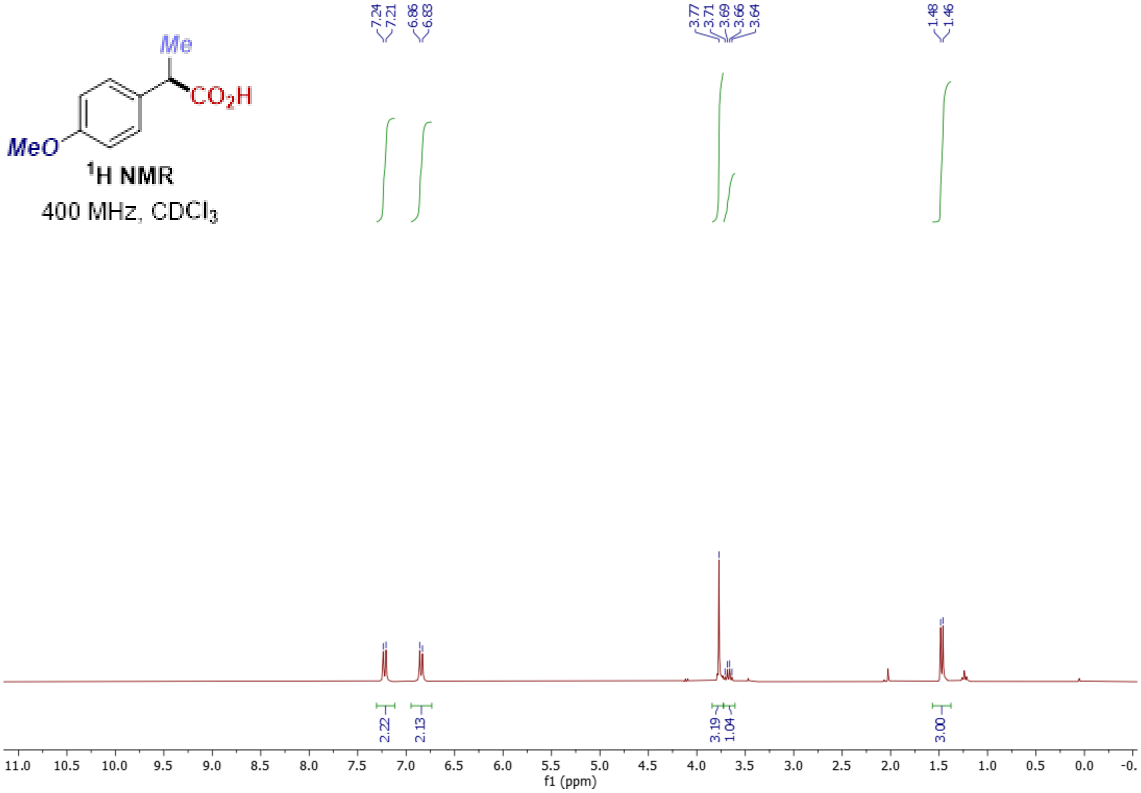

21 - ^{1}H NMR ($CDCl_3$)

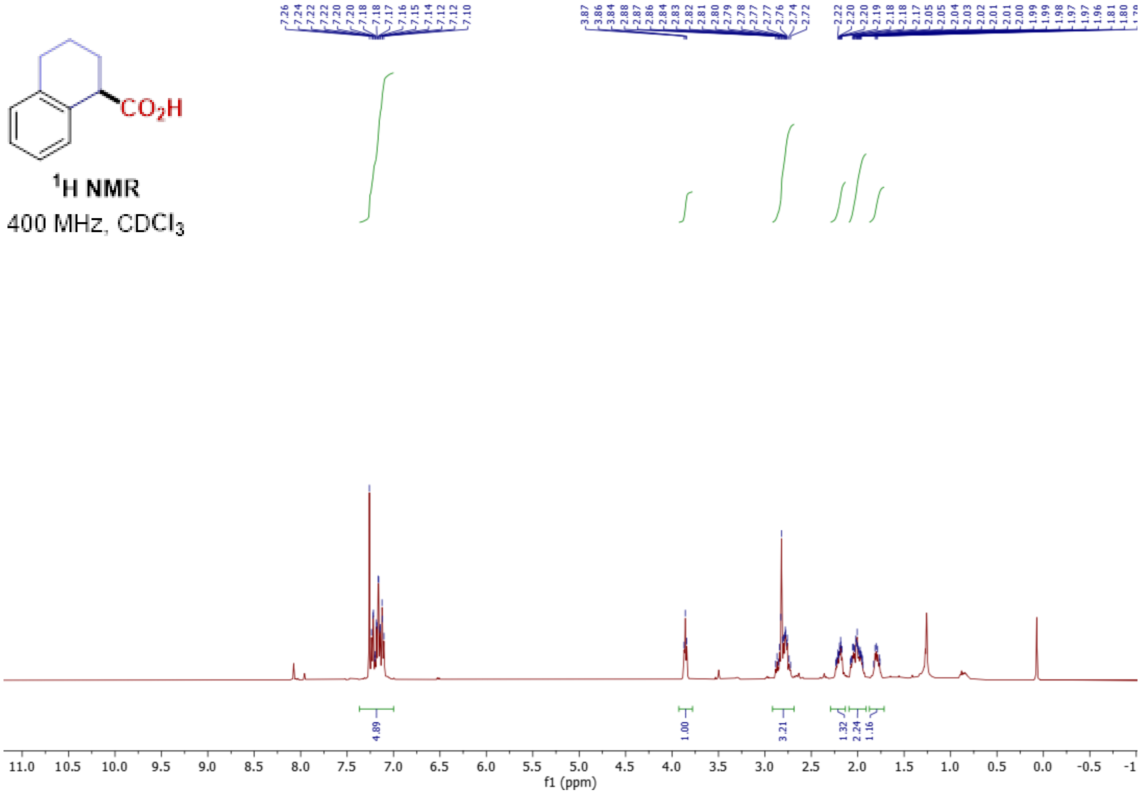

22 - ^{1}H NMR ($CDCl_3$)

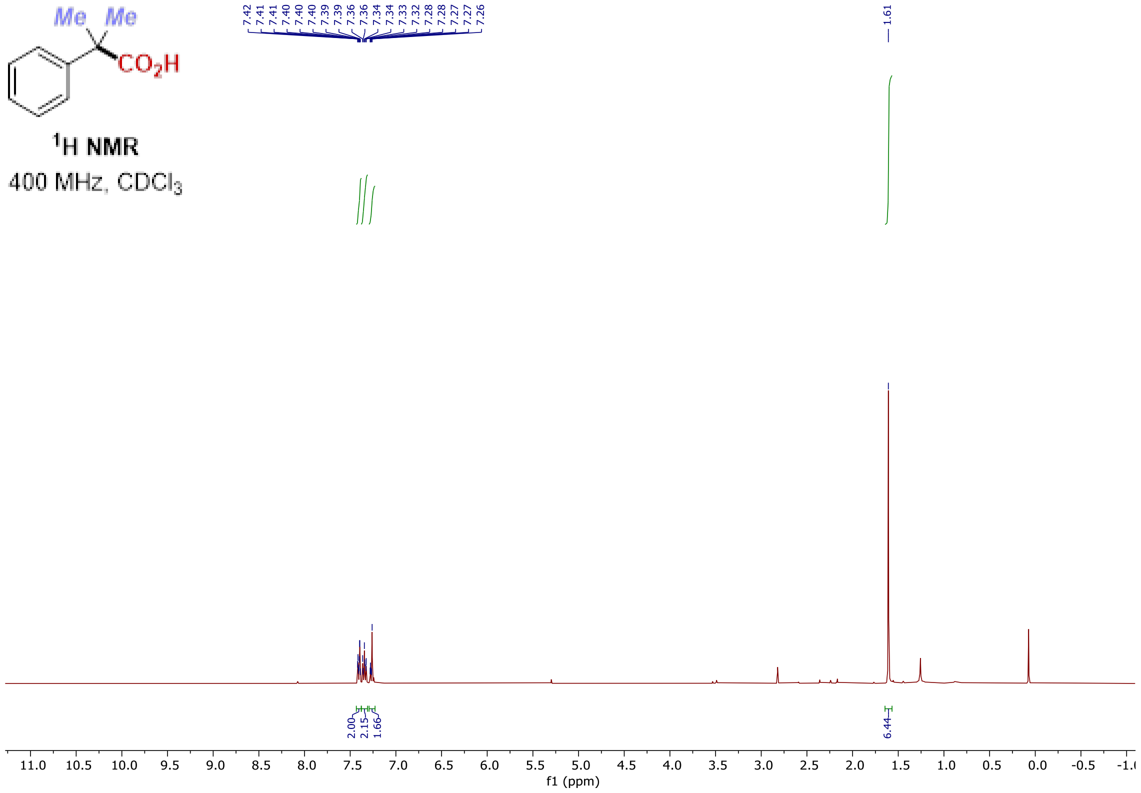

23 - ^{1}H NMR ($CDCl_3$)

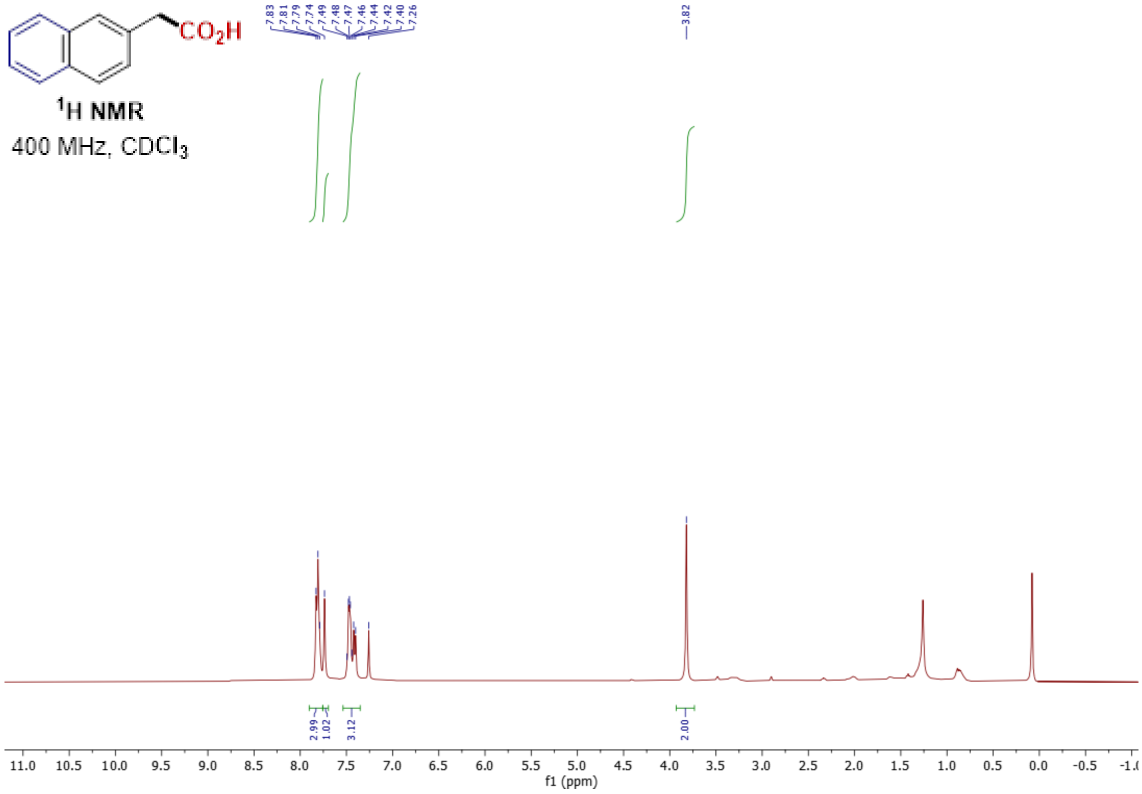

24 - ^{1}H NMR ($CDCl_3$)

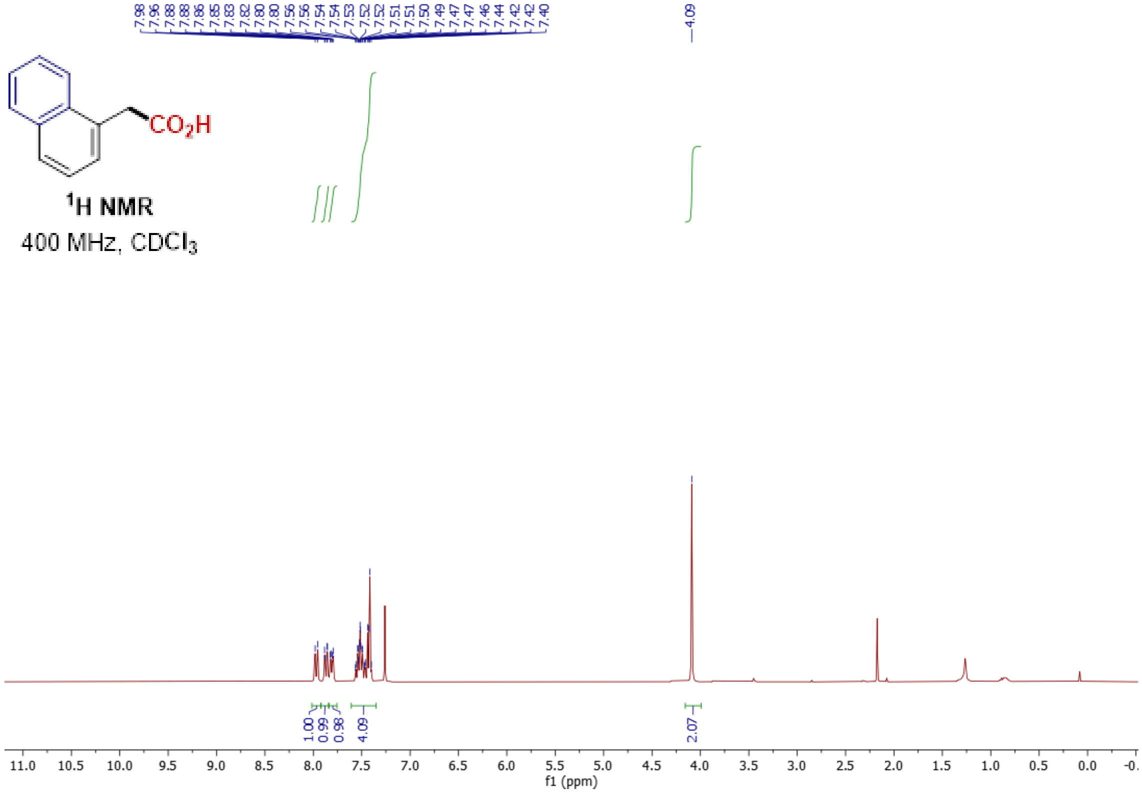

25 - ^{1}H NMR ($CDCl_3$)

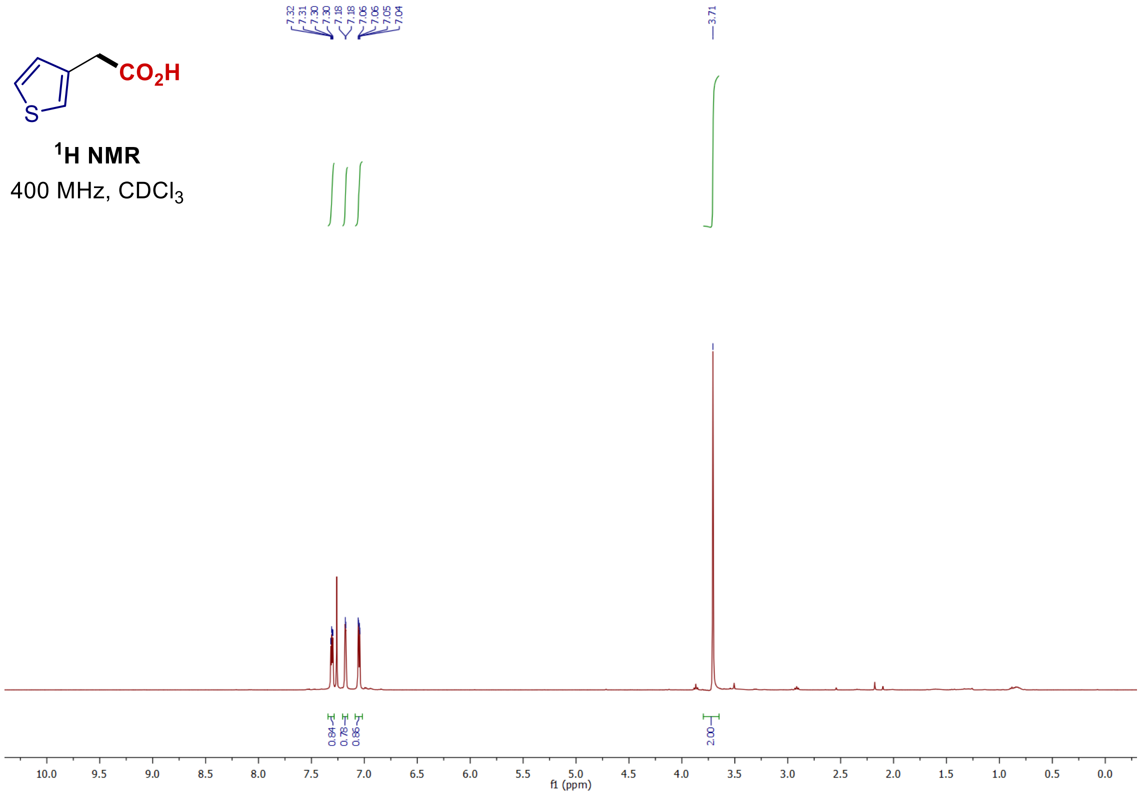

25 - $^{13}C\{^1H\}$ NMR ($CDCl_3$)

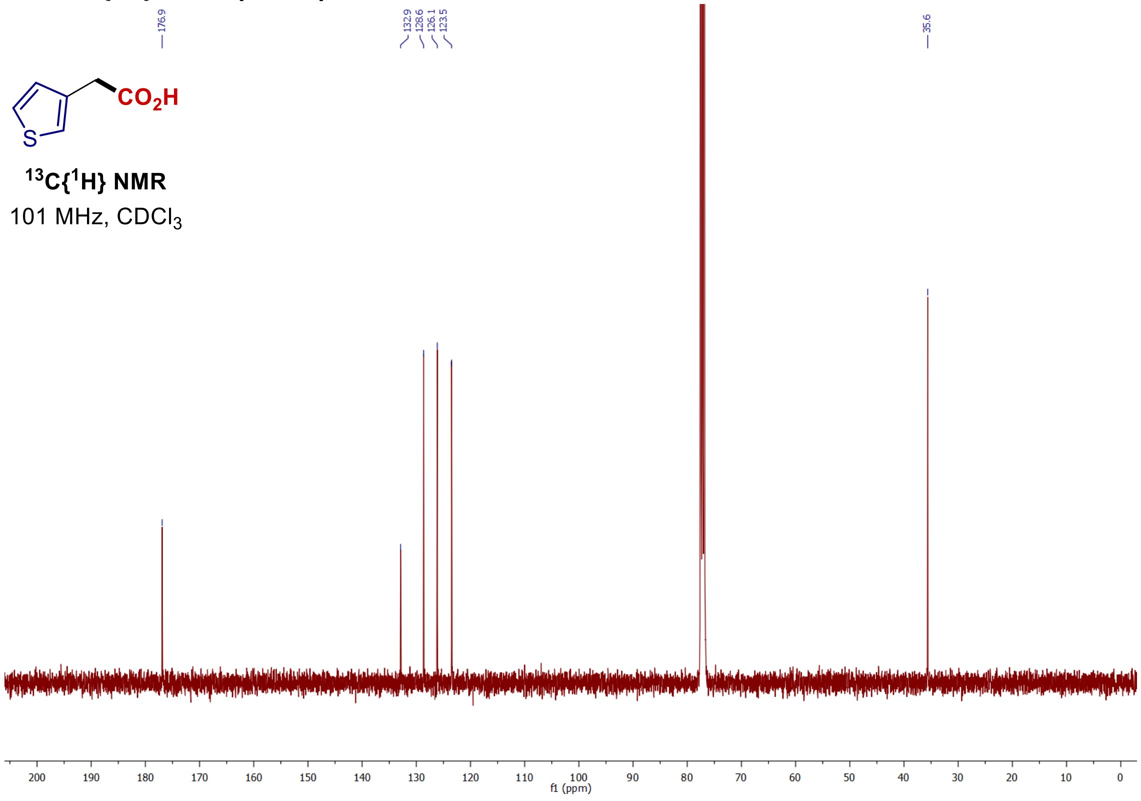

26 - ^1H NMR ($CDCl_3$)

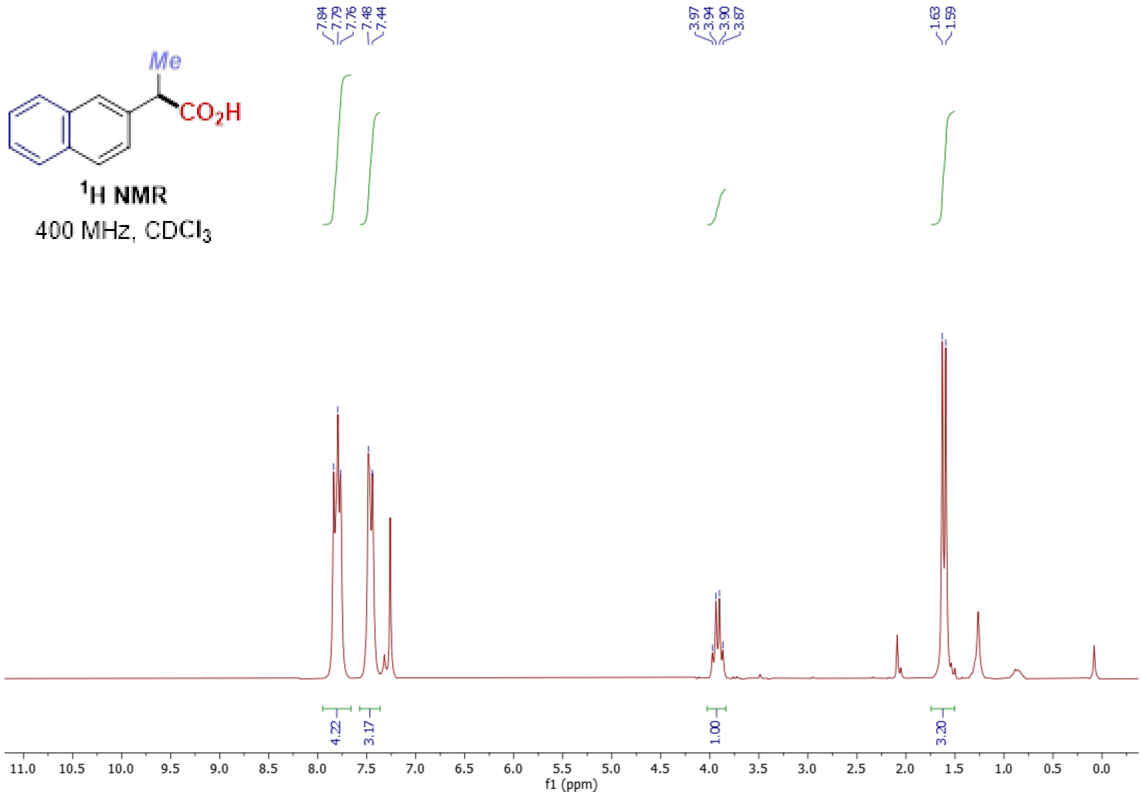

27 - ^{1}H NMR ($CDCl_3$)

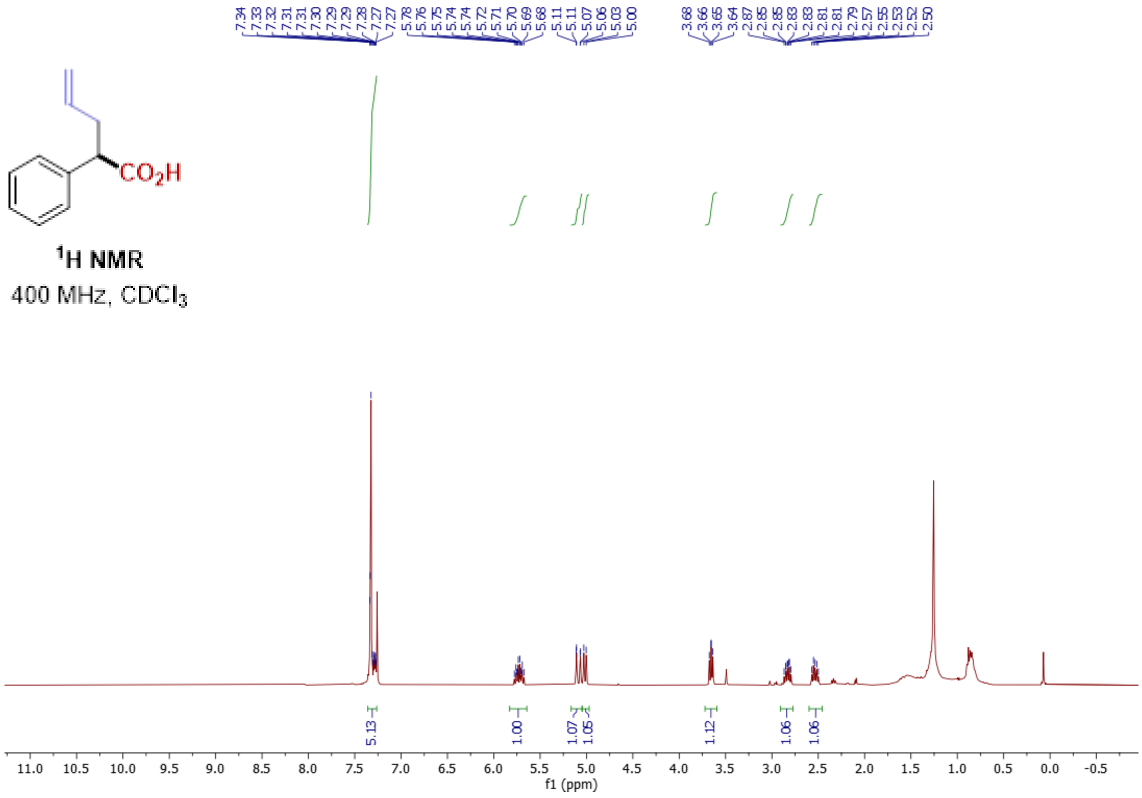

29 - ^{1}H NMR ($CDCl_3$)

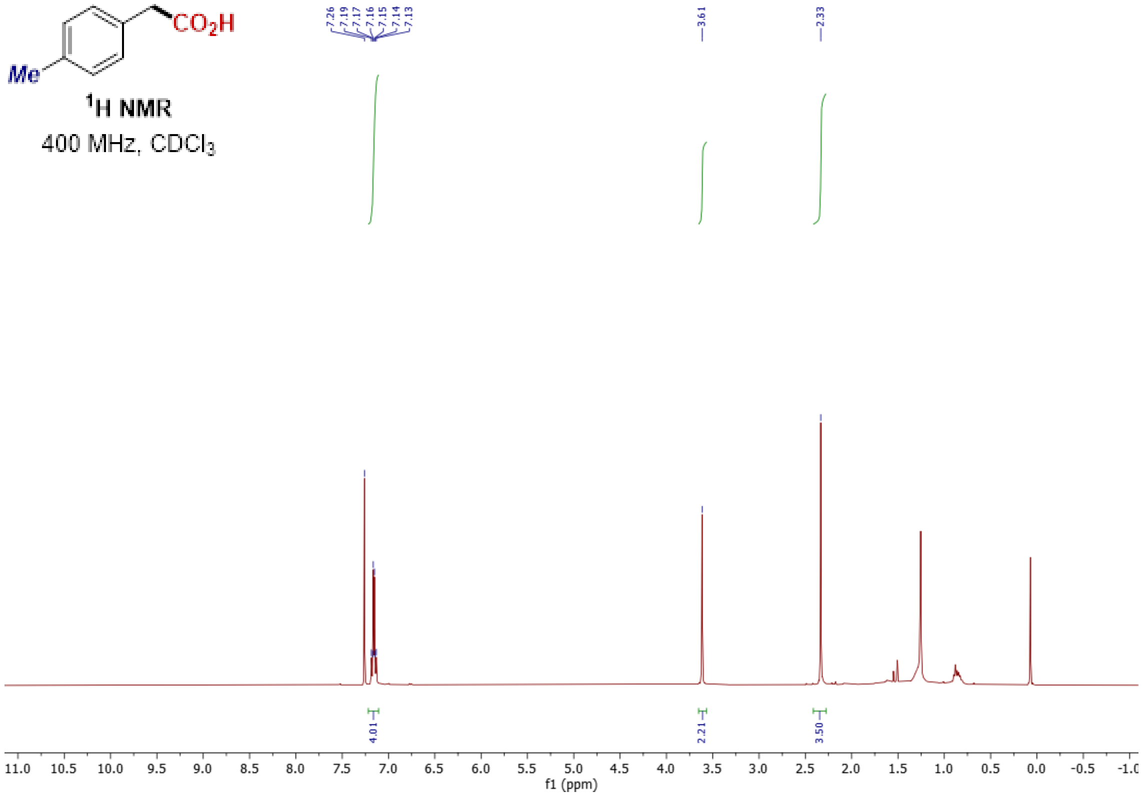

30 - ^{1}H NMR ($CDCl_3$)

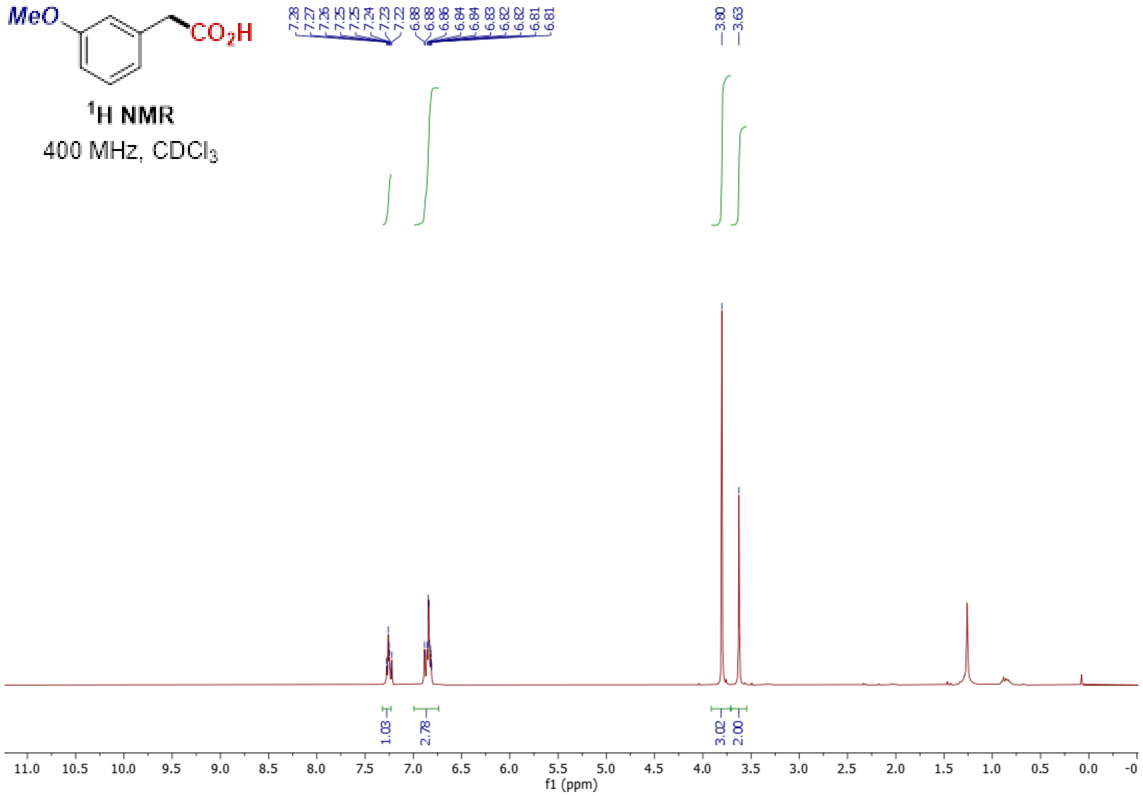

Supplement: Supplementary file 1 — jo2c02952_si_001.pdf [file jo2c02952_si_001.pdf]
